# Catalytic Dioxygen Activation Using a Diketopiperazine and a Manganese Catalyst for Selective C(sp^3^)–H Oxidation

**DOI:** 10.1021/jacsau.5c00759

**Published:** 2025-07-28

**Authors:** Kyriaki Gennaiou, Arnau Call, Nikos Siakavaras, Miquel Costas, Alexandros L. Zografos

**Affiliations:** † Department of Chemistry, 37782 Aristotle University of Thessaloniki, University Campus, 54124 Thessaloniki, Greece; ‡ Institut de Química Computacional i Catàlisi (IQCC) and Departament de Química, 430179 Universitat de Girona, Girona, Catalonia E-17003, Spain

**Keywords:** aerobic oxidation, nonheme metal-oxo catalysis, C−H functionalization, site-selective oxidation, green oxidation, biomimetic oxidation, organocatalyst-metal synergy, O_2_ activation

## Abstract

Activating dioxygen for the selective oxidation of alkanes remains a significant challenge in chemical synthesis. A key limitation lies in identifying efficient electron donors that can partially reduce and thus activate dioxygen while remaining stable in the presence of the resulting reactive oxygen species. Additionally, uncontrolled radical pathways often compromise chemoselectivity in reactions where O_2_ is used as oxidant. In this study, we report a pyrrole-proline diketopiperazine (DKP) catalyst that synergizes with manganese complexes to activate dioxygen for selective C–H oxidation of alkanes. This strategy achieves high chemoselectivity across diverse substrates under aerobic conditions and delivers moderate to good yields, particularly for benzylic and strained cyclic alkanes.

## Graphical abstract

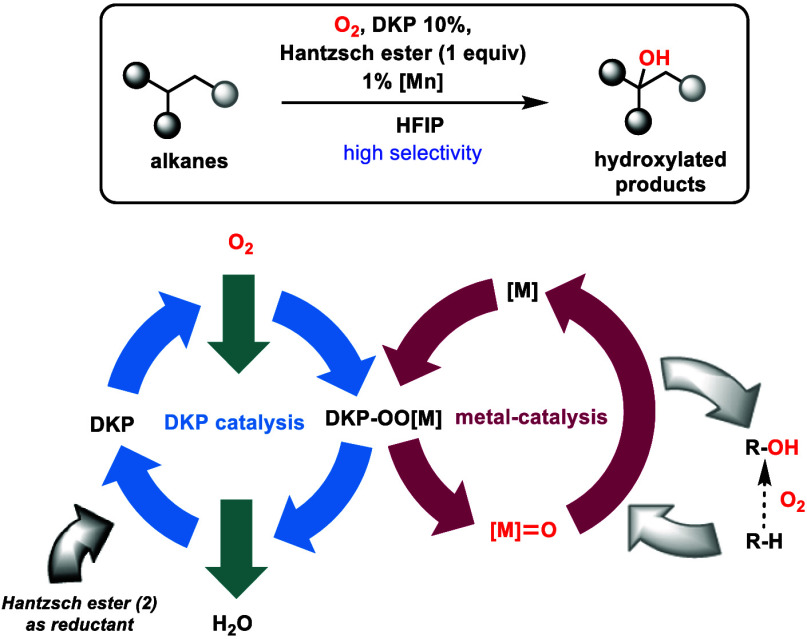

## Introduction

Natural oxidation processes rely on oxygenases to drive a variety of biosynthetic pathways.^1–5^ Oxygenase mechanisms are rooted in electron transfer chains, in which reductase enzymes deliver electrons to oxygenase-bound cofactors. These cofactors, in turn, transfer electrons to dioxygen in a highly controlled manner, generating (per)­oxo intermediates that act as active oxidants for diverse oxidative transformations (Scheme 1A).^6–11^ Due to the spin-forbidden nature of dioxygen, its activation is restricted to reactions involving one-electron donors.^12^ While nature universally employs NAD­(P)­H-dependent reductases for the initial electron transfer, the subsequent activation of dioxygen varies depending on the cofactor involved, as each transformation requires a specific oxidation potential (Scheme 1A).^13,14^ The repertoire of cofactors includes low-redox-potential organocatalysts, such as flavins, as well as metal-based systems–most notably iron and copper complexes, as found in P_450_ monooxygenases–that are capable of oxidizing nonactivated C–H bonds.^6–11^ The remarkable substrate selectivity exhibited by such enzymes arises from the highly structured architecture of the active site and the specificity imparted by their associated reductases.^15–17^

**Scheme 1:**
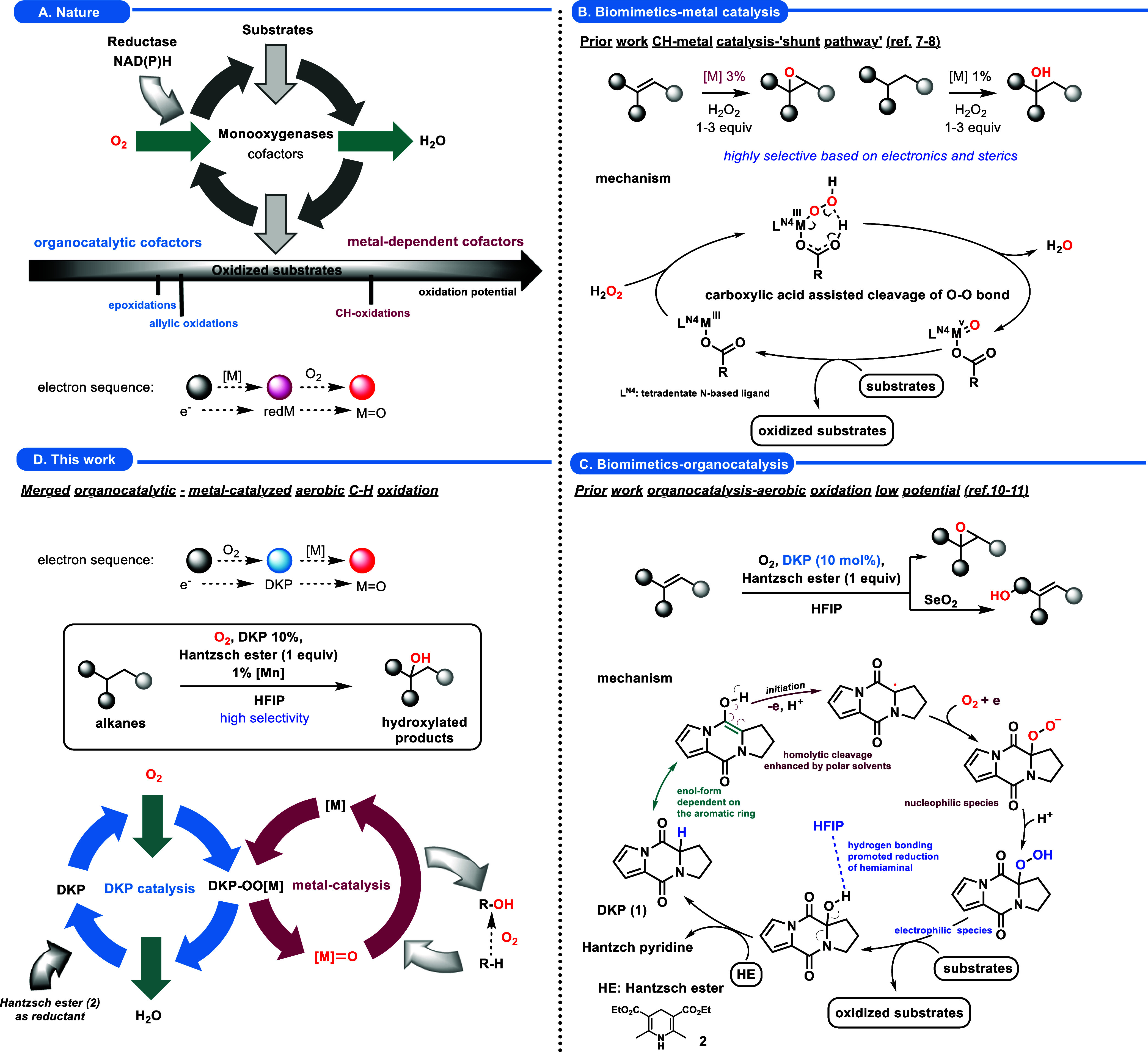
Aerobic Oxidation, under the Lenses of Monooxygenases (A), Prior (B, C) and Current Work (D)

Despite their impressive ability to catalyze selective oxidations, oxygenases remain challenging to implement under standard laboratory practices. Simplified aerobic monooxygenase mimics have proven difficult to utilize, primarily due to the chemical mismatch between the reductants (electron donors) and the reactive oxygen species generated during dioxygen activation. Consequently, only a few examples of such models have been reported,^18–26^ often relying on ‘shunt pathway’ biomimetics that circumvent the need for external reductants in synthetic applications. These shunt pathways typically substitute dioxygen with stoichiometric oxidants such as peroxides, which effectively supply the electrons that would otherwise be supplied by an external reductant. Representative methods include the use of flavins or flavin mimics, as well as heme and nonheme metal catalysts, which mediate a range of oxidative transformations–including heteroatom oxidations, epoxidations, allylic and C–H bond oxidations (e.g., metal catalysis shown in Scheme 1B)^27–39^ –often achieving impressive selectivity even in structurally complex substrates.^40–42^

Other strategies, such as the use of well-defined radical generators, offer alternatives to harness dioxygen’s oxidation potential, but they fail to achieve high selectivity.^43–51^ As a result, the development of a nonenzymatic, selective method for aerobic C–H oxidation remains a significant and unresolved challenge in synthetic chemistry.

To address this challenge, a novel approach is developed in the current work by modifying the electron donation sequence. Whereas Nature typically channels electron to the metal center prior to engagement with dioxygen, we investigated whether dioxygen could instead be introduced earlier in the process—before metal center is involved (Scheme 1A,D). This strategy required identifying a suitable mediator capable of efficiently delivering activated dioxygen to the metal center. Equally critical was the channeling of the reactivity of the resulting dioxygen species to prevent the generation of unselective oxygen-centered radicals, such as superoxide or hydroxyl radicals. Ideally, the activation of dioxygen will result in heterolytic O–O cleavage, forming a high valent metal-oxo oxidant. Another key consideration was the selection of a sacrificial reductant (initial electron donor) that could be gradually consumed in the presence of terminal metal-oxo oxidants. (Scheme 1D). This strategic modification aims to overcome the limitations of conventional aerobic oxidation methods–particularly their challenges with selectivity–while opening new avenues for controlled, selective oxidations through direct dioxygen activation.

Pyrrole-proline diketopiperazine (DKP) (**1**) has recently been demonstrated as an effective catalyst for activating dioxygen in the presence of Hantzsch ester (**2**) (Scheme 1C).^52^ This method has proven successful in the aerobic oxidation of heteroatoms, epoxidation, and allylic oxidation of alkenes,^53^ even when applied to structurally complex substrates.^54^ Furthermore, DKP was later found to synergize with redox-active metals, enabling the coupling of boronic acids to bisphenols and diaryl ethers via radical chain processes.^55^ This intriguing discovery prompted us to investigate DKP’s potential in synergistic metal catalysis, particularly for achieving C–H oxidations.

With attention to recent advancements in manganese and iron catalysis for the oxidation of unactivated C–H bonds using hydrogen peroxide,^28–33,40–42^ we examined their performance in the presence of DKP catalysis. The stability of DKP-derived oxygen species—which effectively prevents the formation of O-centered radicals, as evidenced by its selective epoxidation of complex substrates^53^ makes the synergy between DKP and metal catalysis particularly promising for selective C–H oxidations.

## Results and Discussion

We initiated our investigations using the saturated geraniol acetate derivative (**3**) as a model substrate for aerobic C–H oxidation (Table 1). The DKP-catalyzed protocol, utilizing 10 mol % of the DKP organocatalyst (**1**) and a stoichiometric amount of Hantzsch ester reductant (**2**) in hexafluoroisopropanol (HFIP), was tested under a dioxygen atmosphere (1 atm) in the presence of 1 mol % of various redox-active complexes (see Table 1), which are known to catalyze C–H oxidation using H_2_O_2_.^27–39^ While the iron complex (*R,R*)-Fe­(^TIPS^pdp) in combination with DKP under aerobic conditions did not lead to the formation of oxidation products (Table 1; entry 2), manganese catalysts enabled the formation of the hydroxylated product (**4**), via selective oxidation at the terminal tertiary C–H bond, in yields ranging from 2 to 3%. In addition, traces of the 3-hydroxylated congener (**5**) were detected, arising from oxidation at more sterically hindered tertiary C–H sites (Table 1, entries 3–5). The yield could be maximized up to 4% with the sterically encumbered catalyst (*R*,*R*)-Mn­(^TIPS^pdp) (entry 5). Despite the well-established role of acids in promoting peroxide cleavage and facilitating the formation of metal-oxo complexes (Scheme 1B),^56–58^ no further improvement was observed upon the addition of acetic acid (entry 6). This outcome was unexpected and is attributed to a likely side reaction between acetic acid and the Hantzsch ester, resulting in no effect.

**Table 1:**
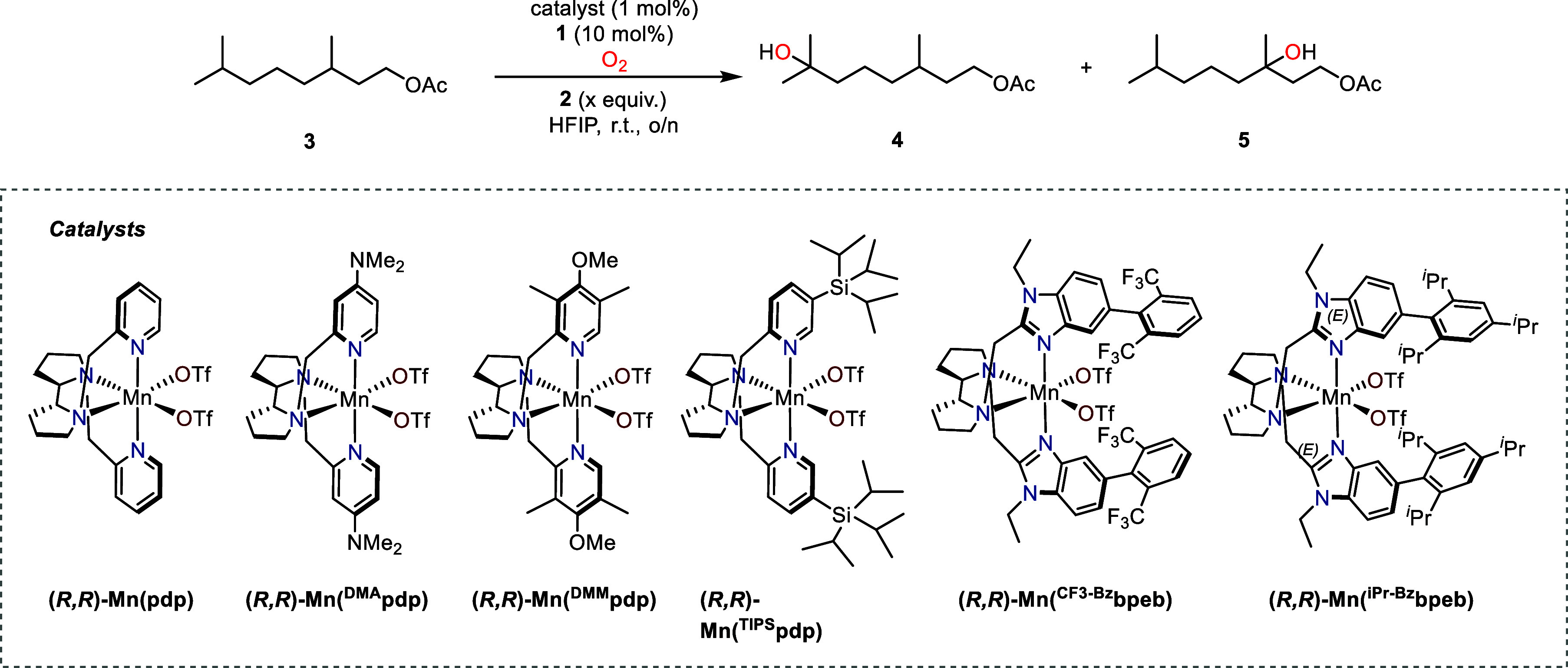

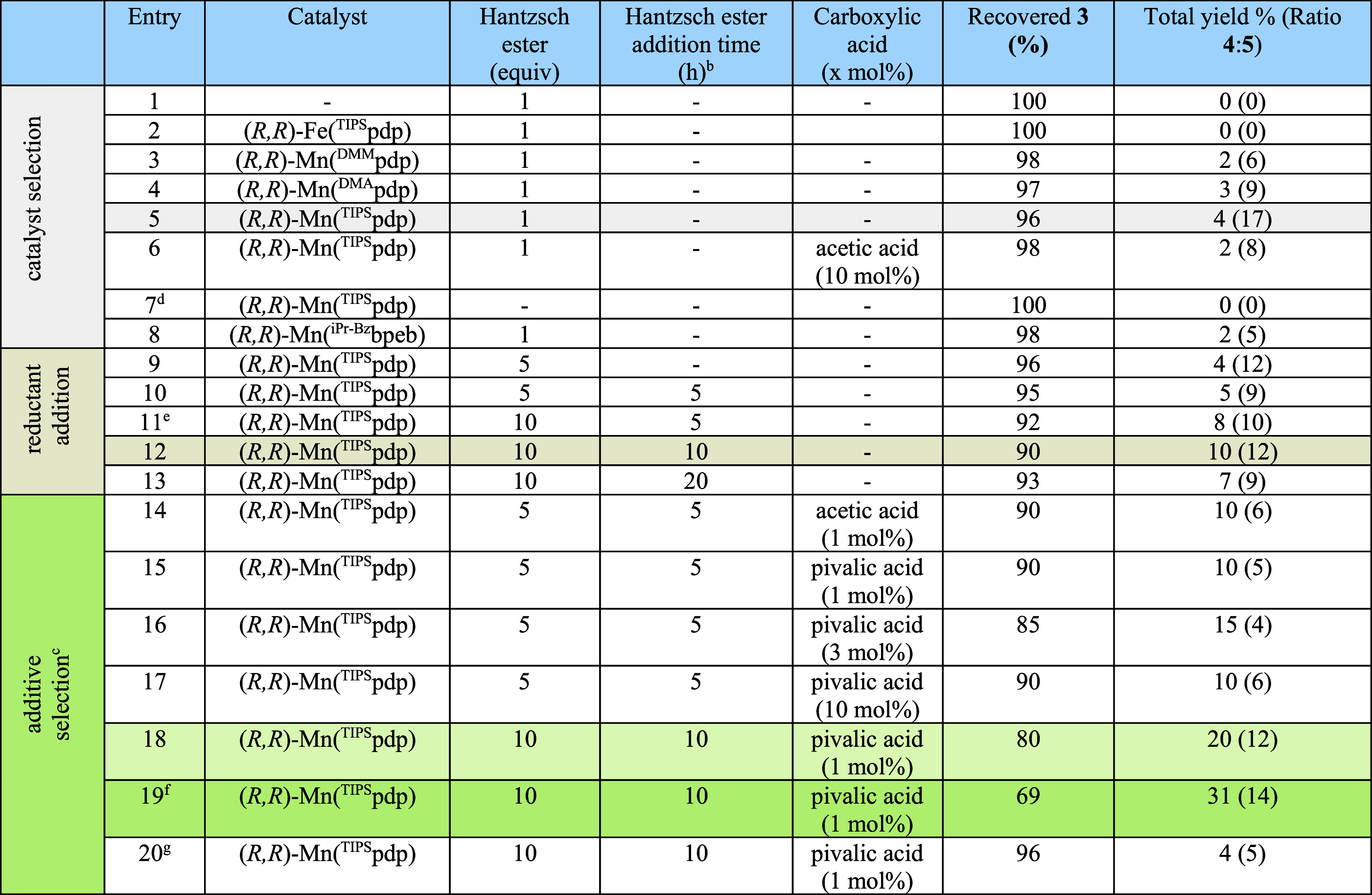
Optimization for Synergistic DKP-Metal Aerobic Oxidation

a Conditions: substrate (0.05 mmol), catalyst (1 mol %), DKP (10 mol %), Hantzsch ester (HE, 1–10 equiv), HFIP (2 mL), stirred overnight at room temperature under dioxygen bubbling.

b For syringe pump additions, an initial portion of 2 equiv of HE was added to the reaction mixture. The remaining equivalents were delivered as a 0.07 M solution in HFIP at a rate corresponding to the time indicated in the table.

c Substrate (0.05 mmol) and (*R,R*)-Mn­(^TIPS^pdp) (1 mol %) catalyst were premixed with the indicated acid for 10 min before its addition to reaction mixture.

d The reaction was performed in the absence of both DKP and HE.

e HE concentration adjusted to 0.14M.

f An additional portion of DKP (10 mol %) and premixed catalyst-pivalic acid (1 mol %) was added after 5 h of reaction.

g Reaction in the absence of DKP. Yields were determined by GC analysis using an internal standard.

Although the initial attempts yielded low overall yield, the high selectivity observed in the product distribution in some cases (**4**:**5** > 17:1) suggests the involvement of a selective oxidant, akin to those generated via metal-oxo pathways.^40–42^

To further optimize this aerobic oxidation, we sought to unravel the underlying factors governing this transformation. To discard a potential reaction between DKP and the (*R*,*R*)-Mn­(^TIPS^pdp) metal center in HFIP, the components were stirred in stoichiometric quantities in the absence of dioxygen for several hours without any observable reactivity (Scheme 2A).

**Scheme 2:**
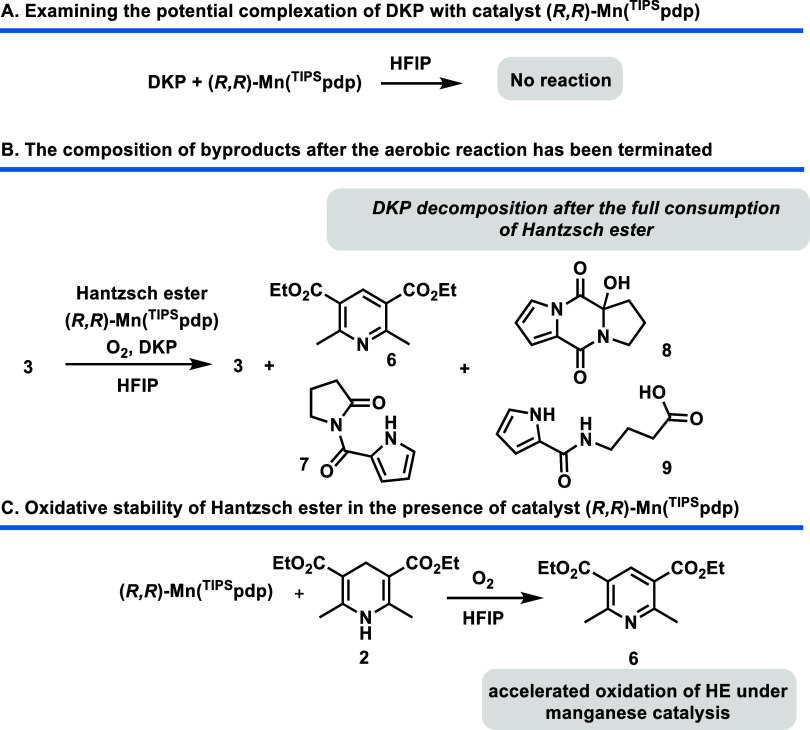
Reaction Profile for Aerobic Oxidations

Further insights were gained from reaction monitoring by NMR, which consistently revealed the absence of Hantzsch ester (**2**) and the exclusive formation of Hantzsch pyridine (**6**), alongside unreacted substrate **3** as the major components in all examined mixtures. Additionally, oxidative products such as amide **7**, hemiaminal **8**, and acid **9** were isolated, indicating complete degradation of DKP under the reaction conditions (Scheme 2B). The Mn-catalyzed oxidative decomposition of DKP was subsequently monitored by ^1^H NMR, using 0.5 equiv of DKP, 10 mol % of (*R,R*)-Mn­(^TIPS^pdp), and 1 equiv of Hantzsch ester, stirred under dioxygen in the absence of substrate. The analysis revealed that no DKP oxidation occurred while Hantzsch ester was still present, suggesting that oxidative cleavage of DKP proceeded via a previously described aerobic decomposition pathway, initiating only after full conversion of the Hantzsch ester to pyridine **6**.^52^

Finally, a kinetic experiment was conducted to evaluate the impact of metal catalysis on the aerobic oxidation of the Hantzsch ester in the absence of the DKP catalyst (Scheme 2C). The results demonstrated a significant acceleration in the oxidation of Hantzsch ester (**2**) to Hantzsch pyridine (**6**), which was quantitatively formed within an hour. In contrast, the same transformation required several hours under dioxygen and HFIP conditions when no metal catalyst was present. This oxidation is tentatively attributed to the electron donation from the Hantzsch ester to a steady-state manganese­(III) species, generated via O_2_ oxidation. This process results in the formation of Hantzsch pyridine and a manganese­(II) complex.^59^ Such a pathway accounts for the rapid depletion of Hantzsch ester ensuring the oxidative decomposition of DKP, ultimately halting the entire process.

Based on these findings, two optimization strategies were pursued to enhance the C–H oxidation process: (i) increasing the bulkiness of the manganese catalyst to potentially slow electron transfer from the Hantzsch ester to the metal center, and (ii) maintaining a steady supply of Hantzsch ester throughout the reaction.

Using a bulkier catalyst, (*R,R*)-Mn­(^iPr‑Bz^bpeb), resulted in diminished yields (Table 1; entry 8). In contrast, increasing the amount of Hantzsch ester and delivering it steadily via syringe pump crucially improved reaction efficiency. A 10 h addition of 10 equiv at 0.07 M proved most effective, affording a 10% combined yield of oxidized products **4** and **5**, with good selectivity (4:5 = 12), (Table 1; entry 12). Interestingly, no additional products arising from methylene oxidation were detected.

Despite the observed ineffectiveness of acetic acid in the presence of Hantzsch ester (Table 1; entry 6), we prioritized incorporating an acidic additive into the process due to its importance in facilitating the rapid formation of oxo-metal intermediates.^56–58^ To prevent the direct reaction between the carboxylic acid and the basic Hantzsch ester, we explored a premixing strategy in which the (*R*,*R*)-Mn­(^TIPS^pdp) catalyst was combined with the carboxylic acid coligand prior to its introduction into the reaction mixture. This protocol significantly impacted the catalytic performance, with pivalic acid (p*K*_a_ 5.01) emerging as the most effective acid coligand among those tested. Using an equivalent amount of pivalic acid relative to the catalyst led to a remarkable improvement in product yields, reaching 20% (Table 1; entry 18) with a 10 h addition of Hantzsch ester. Finally, the addition of a supplemental portion of DKP (10 mol %) alongside the premixed catalyst with pivalic acid (1 mol %) after 10 h of reaction further improved the yield, achieving a satisfactory 31%, which corresponds to 30 catalytic turnovers involving dioxygen activation (Table 1; entry 19).

As anticipated, control experiments excluding DKP (**1**), Hantzsch ester (**2**), or the metal complex resulted in a complete absence of oxidation products or traces under exposure to dioxygen (Table 1; entries 1, 7, and 20).

With optimized reaction conditions established (entries 18 and 19), we expanded the scope of aerobic oxidation for nonactivated C–H bonds and compared the results with a referenced hydrogen peroxide variant (Scheme 3).^38^ Saturated geraniols, functionalized with various electron-donating and electron-withdrawing groups, were tested under aerobic conditions, yielding between 14 and 39% (Scheme 3; compounds 3, 10–12). Although the yields were lower compared to the hydrogen peroxide variant (28–71%), the reactions consistently exhibited superior regioselectivity for the C-7 position.

**Scheme 3:**
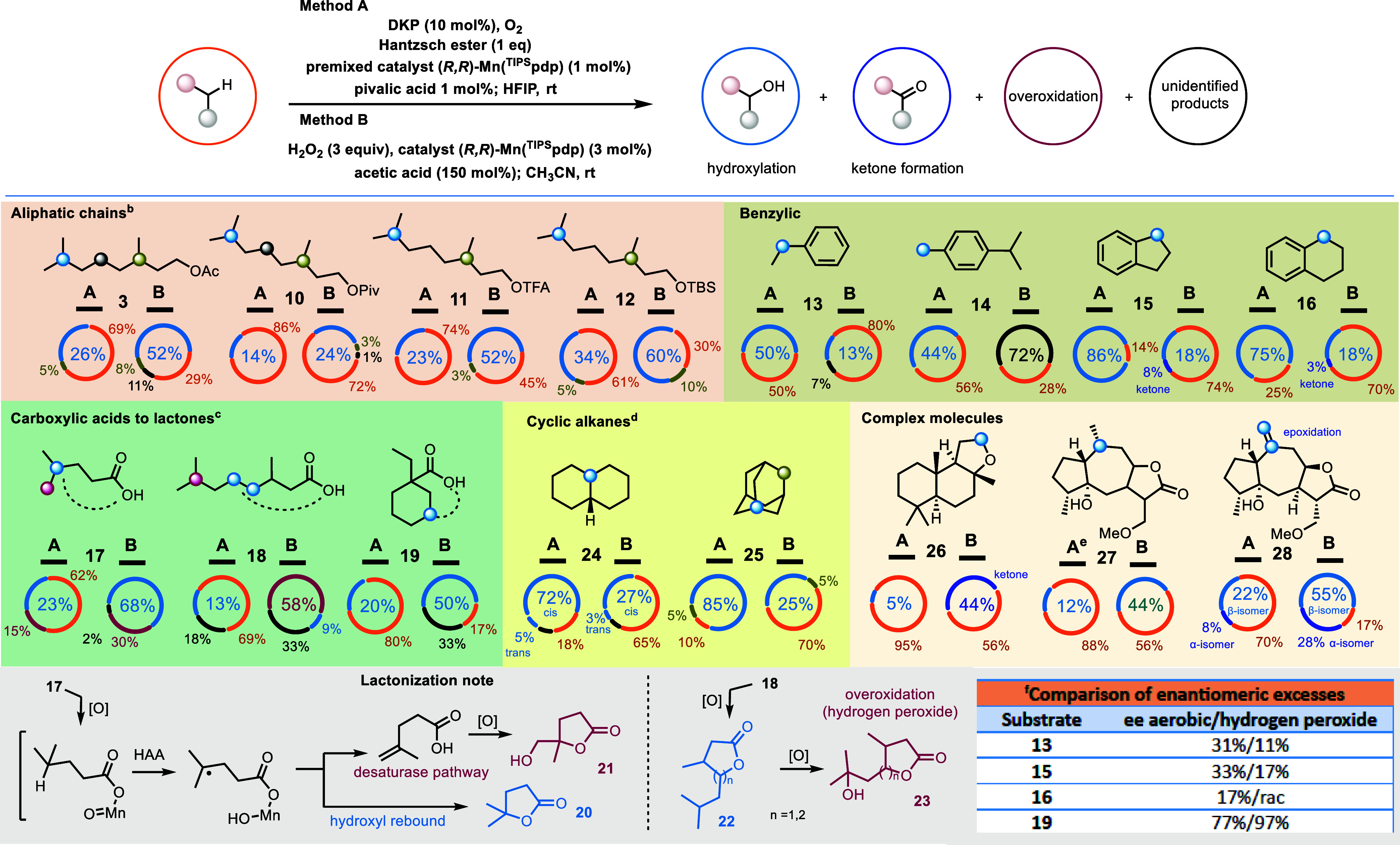
Reaction Scope of Aerobic CH-Oxidation Reaction in Comparison with Hydrogen Peroxide C–H Oxidation

^a^
Conditions: Method A (aerobic oxidation): substrate (0.1–0.15 mmol), according to optimized conditions shown in Table 1 entry 18, unless otherwise noted. Method B (oxidation with H_2_O_2_): H_2_O_2_ (3 equiv), catalyst (3 mol %) with acetic acid (150 mol %) in CH_3_CN, from 0 °C to room temperature (rt). The hydrogen peroxide protocol was performed according to previously reported procedures.^38^

^b^
According to Table 1, entry 19.

^c^
Method B (oxidation with H_2_O_2_): H_2_O_2_ (3 equiv), catalyst (3 mol %) in HFIP, from 0 °C to room temperature (rt).

^d^
Solvent HFIP/DCM (3:1).

^e^
For compound **27**, the (*S,S*)-Mn­(^TIPS^pdp) catalyst was used under premixing conditions.

^f^
For the comparison of enantiomeric excesses, analogous methods were employed. Aerobic method: according to Table 1, entry 18 using (*R,R*)-Mn­(^CF3‑Bz^bpeb). Hydrogen peroxide method: substrate (0.05 mmol), H_2_O_2_ 50% (0.45 M in HFIP, 0.5 equiv, 30 min addition), (*R,R*)-Mn­(^CF3‑Bz^bpeb) (1 mol %), pivalic acid (1 mol %) (see Table 1). Note on colors: orange = starting material; blue = oxidation products; purple = overoxidation; black = unidentified products.

Benzylic substrates consistently outperformed the hydrogen peroxide variant in all examined cases (Scheme 3; compounds **13–16**). Under aerobic conditions, high yields of benzylic alcohols (44–86%) were obtained without any evidence of overoxidation or oxidation of the aromatic ring. In sharp contrast, the hydrogen peroxide method yielded poor results (13–18%), accompanied by overoxidation and unidentified byproducts that likely arise from the well-known competitive oxidation of nondeactivated aromatic rings.^60,61^ Notably, the aerobic oxidation of cymene (**14**) yielded only the monosubstituted hydroxymethyl product (44%), whereas the hydrogen peroxide method produced a complex mixture of unidentified products. Benzylic substrates were successfully scaled up to 0.5 mmol without any erosion on the yields.

When carboxylic acids were used as substrates, directed lactonization at tertiary or secondary positions was observed (Scheme 3; compounds **17–19**). Notably, substrate **17** afforded not only the expected butenolide product but also the overoxidized lactone **21** under both aerobic and hydrogen peroxide conditions. This side product likely arises from an epoxidation-lactonization sequence involving a terminal alkene generated via a desaturation pathway (Scheme 3; lactonization note). Interestingly, the desaturation pathway appeared exclusively in cases involving directed intramolecular hydroxyl rebound, as previously reported.^62^ In the case of compound **18**, where tertiary lactonization is highly strained, nonselective aerobic lactonization at the secondary C–H bonds forming five and six membered heterocycles (compound **22**) was favored, albeit with low yields (13%). Moreover, both oxidation conditions produced high yields of unidentified alkene products, likely stemming from unselective activation of the desaturase pathway.

Under aerobic conditions, cyclic aliphatic compounds **24** and **25** produced yields ranging from 77 to 90%. Notably, *cis*-decalin achieved an 82% yield under optimized conditions, although with modest diastereocontrol (*cis*/*trans* alcohol = 4:1), suggesting the partial involvement of long-lived carbon-centered radicals. This behavior was attributed to the complete insolubility of *cis*-decalin in HFIP, leading to partial uncontrolled oxidation in the dioxygen stream. When DCM was introduced as a cosolvent (HFIP/DCM = 3:1), the yield of *cis*-decalol reached 72%, with a diastereomeric ratio exceeding 14:1. This highlights the critical role of solubility and effective stirring in achieving high selectivity. In these cases, aerobic reactions outperformed hydrogen peroxide, delivering superior yields and cleaner reaction profiles.

On the other hand, complex substrates bearing electron-withdrawing groups or heteroatoms showed diminished yields compared to the hydrogen peroxide variant or yielded only trace amounts of products (Scheme 3; compounds **26**–**27**). The pronounced effect of these substituents on product yields is attributed to their hydrogen-bonding deactivation in HFIP solvation.^63^ This effect is further highlighted by the complete absence of overoxidation products in aerobic reactions. Despite the reduced reactivity of C–H bonds under aerobic conditions, complex substrate **28** successfully reacted at the terminal alkene position to form the corresponding epoxide. This stands in stark contrast to previously reported aerobic oxidations relying solely on DKP, which failed to initiate the reaction.^53^ These findings underscore the formation of a new oxidant in the presence of the manganese complex.

To evaluate whether the synergistic effect of DKP promotes the production of a well-defined manganese-oxo complex under aerobic conditions, the enantioselectivity of selected aerobic reactions was assessed and compared to reactions using hydrogen peroxide as the oxidant (substrates **13**, **15**, **16**, and **19**). Optimization of enantioselectivity, achieved through the screening of various catalysts, identified (*R,R*)-Mn­(^CF3‑Bz^bpeb) as the most effective catalyst for enantioselective aerobic oxidations. Reactions with the hydrogen peroxide variant were conducted under similar catalyst and solvent conditions but were terminated early to prevent overoxidation to ketones.

Interestingly, substrates **13**, **15**, and **16** performed better under aerobic conditions, achieving enantiomeric excesses (ee) of 31, 33, and 17%, respectively, compared to 11%, 17% ee and racemate under hydrogen peroxide conditions (Scheme 3, inset). However, substrate **19** showed a decrease in enantioselectivity. Despite the observed differences in enantioselectivities between the two oxidative protocols—likely due to the large excess of Hantzsch ester in the aerobic reactions—these results confirm the intermediacy of a chiral C–H oxidation species, presumably a high valent metal-oxo species akin these formed in H_2_O_2_ reactions.

## Conclusions

In this study, we addressed the longstanding challenge of selective aerobic C–H oxidation by developing a novel methodology inspired by natural monooxygenase mechanisms. Unlike traditional approaches, which often falter due to the chemically mismatched demands of reductants and activated dioxygen species, our strategy innovatively modifies the electron donation sequence by employing DKP as a dioxygen activator alongside manganese complexes. Comprehensive optimization studies revealed that preventing direct interaction between specific additives and the Hantzsch ester was crucial to minimizing undesired side reactions and ensuring efficient oxidation.

## Supplementary Material



## Notes

Detailed experimental procedures, optimization protocols, GC chromatograms, mechanistic study experiments, compound characterization data, and copies of NMR spectra (PDF)
