# Supplementary material for: Catalytic Dioxygen Activation Using a Diketopiperazine and a Manganese Catalyst for Selective C(sp3)–H Oxidation
Source: JACS Au. 2025 Jul 28;5(8):4106–13. doi: 10.1021/jacsau.5c00759 (PMC12381749; doi:10.1021/jacsau.5c00759)
Supplement: Supplementary file 1 [file au5c00759_si_001.pdf]

Supporting Information

Catalytic Dioxygen Activation Using a Diketopiperazine and a Manganese Catalyst for Selective C(sp^3)-H Oxidation

Kyriaki Gennaiou,[1] Arnau Call,[2] Nikos Siakavaras,[2] Miquel Costas,[2]* and Alexandros L. Zografos[1]*

[1]Department of Chemistry, Aristotle University of Thessaloniki, University Campus, 54124, Thessaloniki, Greece; [2]Institut de Química Computacional i Catàlisi (IQCC) and Departament de Química, Universitat de Girona, Girona, Catalonia E-17003, Spain.

alzograf@chem.auth.gr

miquel.costas@udg.edu

Table of contents

	Page
**1. Materials, Methods and Instrumentation**	SI-2
**2. The pyrrole-proline diketopiperazine (DKP) catalytic system**	SI-3
**2.1 Preparation of the DKP catalyst**	SI-4
**2.2 Preparation of Hantzsch ester**	SI-5
**3. The manganese complexes catalytic system**	SI-5
**3.1 Preparation of the manganese catalysts**	SI-6
**4. Synergistic Catalysis Method**	SI-6
**4.1 Developing the method for CH oxidation: Optimization of reaction conditions**	SI-6
**5. Substrate preparation**	SI-19
**5.1. Aliphatic chains**	SI-19
**5.2. Carboxylic acids**	SI-21
**5.3. Complex molecules**	SI-22
**6. Reaction scope of the DKP-Mn co-catalysed aerobic CH-oxidation reaction**	SI-23
**6.1 General procedures for CH oxidation with optimized catalytic conditions**	SI-23
**6.2 Oxidized products**	SI-24
**7. NMR Spectra**	SI-36
**8. References**	SI-62

1. Materials, Methods and Instrumentation

Materials. Reagents and solvents were purchased at the highest commercial quality and used without further purification unless stated otherwise. Anhydrous solvents were dried accordingly. Dry methylene chloride (CH_2Cl_2) was obtained by refluxing the solvent with CaH_2. All solvents were kept under Ar using molecular sieves 4Å in their bottles. Petroleum ether refers to the 40-60°C boiling fraction. Preparation of catalysts were performed in standard, dry glassware, under a nitrogen atmosphere.

Methods. Reactions were monitored by thin-layer chromatography (TLC) carried out on S-2 0.25 mm E. Merck silica gel plates (60F-254), using UV light as visualizing agent (λ_{max}= 254 nm or 360 nm) and ethanolic *p-anisaldehyde* as developing agent, phosphomolybdic acid or by *Seebach* TLC stain solution, followed by heating. E. Merck silica gel (60, particle size 0.040–0.063 mm) was used for flash column chromatography. Removal of organic solvents under reduced pressure was performed using house vacuum (40 torr) at 40 ºC unless otherwise indicated.

Instrumentation. NMR spectra were recorded at 298 K using an Agilent Technologies DD2 500 spectrometer or a Bruker Avance 300MHz spectrometer and calibrated by residual solvent peaks. ^{1}H NMR spectra were recorded at 500 MHz and 300MHz and residual solvent peaks were used as an internal reference ($CDCl_3$ δ_H 7.26 ppm). Data are reported as follows: chemical shift in ppm, multiplicity (s = singlet, brs = broad singlet, d = doublet, t = triplet, q = quartet, m = multiplet or overlap of non-equivalent resonances, coupling constants are reported in Hz, integration is included. ^{13}C NMR spectra were recorded at 125 MHz or 75MHz and residual solvent peaks were used as an internal reference ($CDCl_3$ δ_C 77.00 ppm). Data are reported as follows: chemical shift in ppm, multiplicity deduced. Optical rotations were recorded on a Krüss Optronic polarimeter at 589 nm and are reported in units of 10–1(deg cm^2 g^{-1}). High-resolution mass spectra (HRMS) were recorded on an Agilent ESI-TOF (time of light) mass spectrometer at a 4000V emitter voltage. Chromatographic analyses were performed on a Shimadzu Nexis GC-2030 Gas Chromatograph (Shimadzu Corporation), with column details: Column max temp.: 250.0 °C; Length: 30.0 m; Inner Diameter: 0.25 mm ID; Film Thickness: 0.25 μm; Column flow: 1.86 mL/min; FID Makeup Gas: He, FID Makeup flow: 24.0 mL/min; FID H_2 flow: 32.0 mL/min, FID Air flow: 200.0 mL/min, using biphenyl 0.05M as internal standard. Gas chromatographic analysis for ee evaluation was performed on an Agilent GC- 7820-A chromatograph using a J&W CYCLOSILB column. Supercritical fluid chromatography (SFC) analysis was performed on an Agilent 1260 Infinity II SFC System using CHIRALPAK IB-3 and CHIRALPAK IG-3 columns.

2. The pyrrole-proline diketopiperazine (DKP) catalytic system

A quick look into the aerobic catalytic system

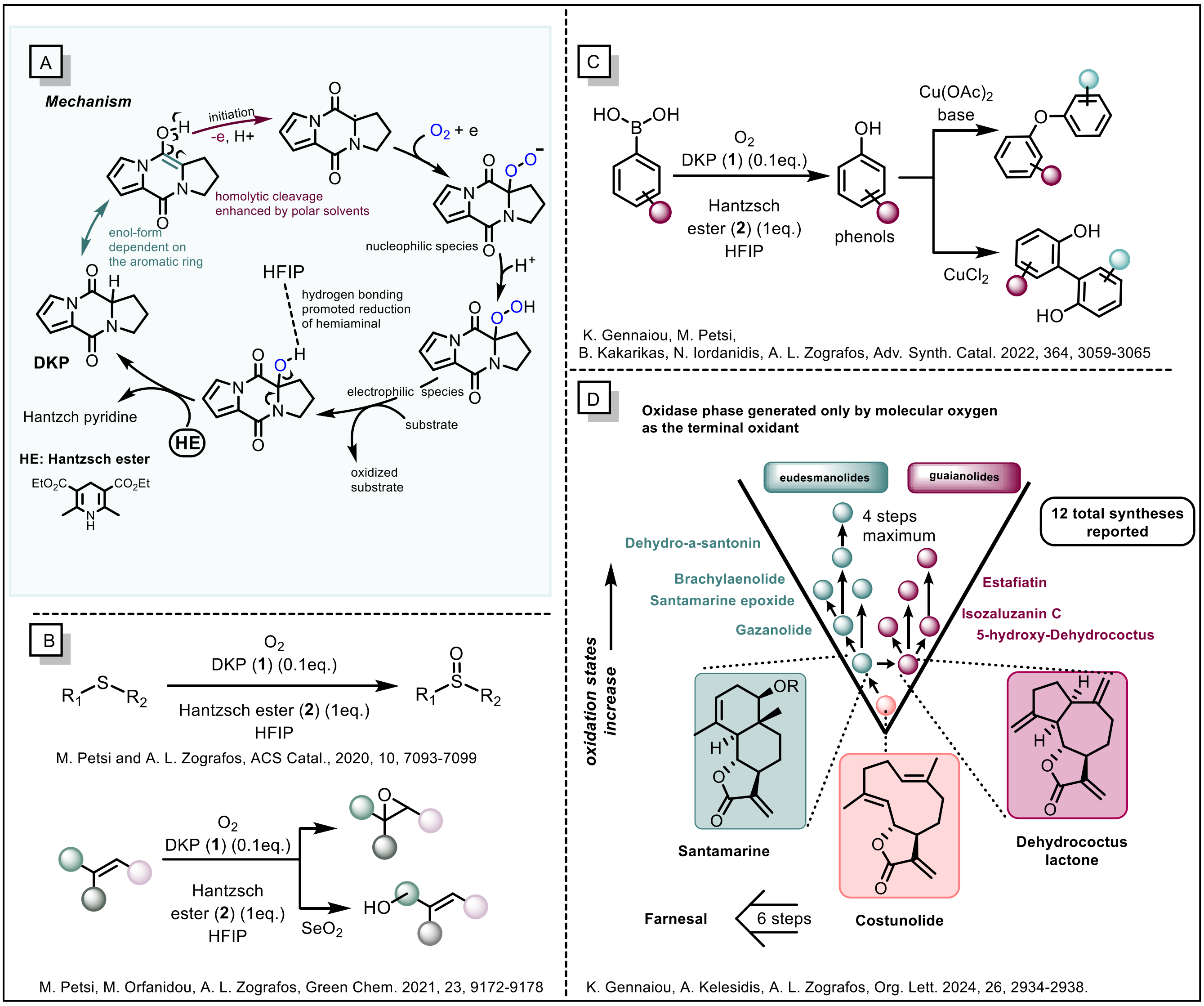

Scheme 1. The pyrrole-proline diketopiperazine catalytic system. A: The catalytic cycle. B, C, D: The application of the aerobic catalytic system in previous works.

Lately, our group reported the development of pyrrole-proline diketopiperazine (DKP) (**1**) as an efficient catalyst to activate dioxygen, allowing the aerobic oxidation of heteroatoms,[1] the epoxidation and the allylic oxidation of alkenes[2] and the oxidative coupling of phenols.[3] This system was also successfully applied in the oxidative decoration of more complex molecules, serving as a selective and mild method to access natural products by divergent synthesis.[4] Its success is relying on the direct formation of peroxy-DKP by mixing DKP organocatalyst with dioxygen, that serves as the active oxidant of the process, while utilization of Hantzsch ester (**2**) ensures the regeneration of the catalyst in a role of a reductase mimic.

2.1 Preparation of the DKP catalyst

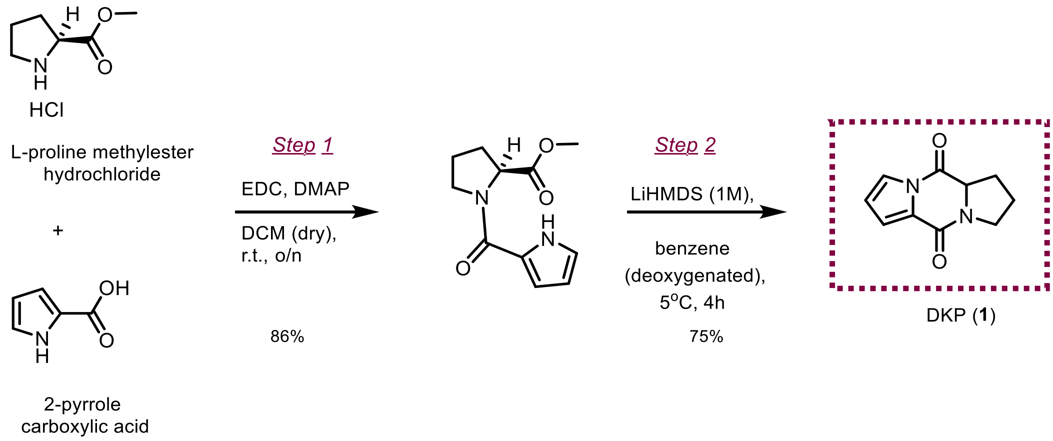

Step 1

To a mixture of 2-pyrrolecarboxylic acid (311.1 mg, 2.80 mmol, 1.0 eq.) and *L*-proline methyl ester (602.9 mg, 3.64 mmol, 1.3 equiv.) in anhydrous DCM (12 mL), 1-(3-dimethylaminopropyl)-3 ethylcarbodiimide (697.8 mg, 3.64 mmol, 1.3 eq.) and DMAP (11.2 mg, 0.092 mmol, 0.033 eq.) were added under argon at 0°C. After 30 min stirring at 0°C and 12hr at room temperature, the reaction mixture was quenched with water and extracted with DCM (2 x 10 mL). The combined organic layers were washed with $NaHCO_3$ (2 x 10 mL) and then dried with $MgSO_4$. After removal of the solvent under reduced pressure and subsequent flash column chromatography of the obtained residue on silica-gel (eluent: hexane/EtOAc 1:1, TLC: *Rf* = 0.3 upon hexane/EtOAc 1:1, UV active on TLC, stains greenish upon *p-anisaldehyde* staining) gave the coupling product in 86% (535.1 mg) yield as a white foam; Recorded NMR was found identical to the reported values.[5] ^{1}H NMR (500 MHz, $CDCl_3$): δ_H 9.67 (brs, 1H), 6.95 (m, 1H), 6.27 (m, 1H), 6.66 (m, 1H), 4.68 (s, 1H), 4.01 – 3.93 (m, 1H), 3.88 – 3.84 (m, 1H), 2.27 – 2.18 (m, 2H), 3.74 (s, 3H), 2.10 – 2.00 (m, 2H) ppm; ^{13}C NMR (125 MHz, $CDCl_3$): δ_C 172.9, 160.4, 125.3, 121.4, 112.6, 110.1, 60.0, 52.2, 48.2, 28.7, 25.3 ppm.

Step 2

Methyl ester (200 mg, 0.90 mmol, 1.0 eq.) was washed with benzene twice, and then dissolved in benzene (6 mL) in a Schlenk tube under argon, which was connected to a vacuum pump and immersed in a dry ice bath. There, the substrate was deoxygenated 3 times and then lithium bis(trimethylsilyl)amide 1M (1.1 ml, 1.08 mmol, 1.2 eq.) was added and the mixture was stirred for 4hr at 5°C. The mixture was quenched with CH_3COOH/CH_3COONa buffer (pH 3.7, 10 mL) and the products were extracted with EtOAc (5 x 10). After removal of the solvents under reduced pressure, the desirable DKP (**1**) product was received as a white solid in 75% (128.3 mg) yield and was used without further purification. TLC: *Rf* = 0.35 upon DCM/EtOAc (1:2), UV active on TLC, stains blue upon *Seebach* staining. Recorded NMR was found identical to the reported values.[1] ^{1}H NMR (500 MHz, $CDCl_3$): δ_H 7.46 (dd, *J* = 3.3, 1.8 Hz,1H), 7.07 (dd, *J* = 3.3, 1.8 Hz,1H), 6.48 (dd, *J* = 3.3, 3.3 Hz, 1H), 4.49 (dd, *J* = 9.5, 6.2 Hz, 1H), 3.88 – 3.80 (m, 1H), 3.70 – 3.61 (m, 1H), 2.60 – 2.54 (m, 1H), 2.19 – 2.13 (m, 1H), 2.10 – 1.96 (m, 2H) ppm; ^{13}C NMR (125 MHz, $CDCl_3$): δ_C 164.5, 155.2, 127.4, 118.8, 117.9, 115.6, 61.5, 44.8, 29.0, 22.3 ppm.

<u>*Notes*</u>

- The reaction for preparation of 2,5-diketopiperazine (DKP) (**1**) was conducted in a Schlenk tube under positive argon pressure. The solvent and materials were carefully degassed with the use of dry ice under freeze-pump-thaw technique. DKP was kept in sealed tubes under positive pressure of dry argon upon preparation and was used in caution of atmospheric air when tubes were opened. In general, DKP can be weighted without the use of a glovebox, but it needs to be protected from air as soon as it comes in contact with polar solvents or base.

2.2 Preparation of Hantzsch ester

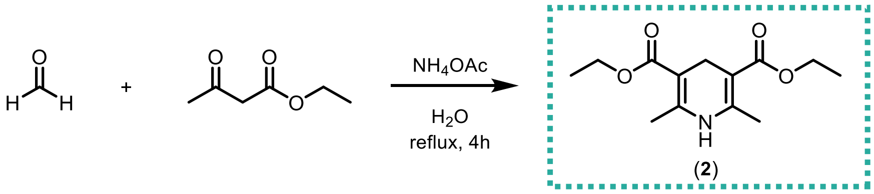

According to literature, formaldehyde solution (1.4 ml, 0.05 mol, 1.0 eq.), ethyl acetoacetate (10.1 ml, 0.08 mol, 1.5 eq.) and ammonium acetate (3.08 g, 0.04 mmol, 0.8 eq.) were dissolved in H_2O (40 ml) in a round bottom flask and stirred at 80°C (using an oil bath) for 4 hours. After the completion of time yellow balls were formed, and the reaction mixture was cooled to room temperature. The resulting solid was filtered, washed with icy water (30 ml) and cold acetone (30 ml) to afford diethyl 2,6-dimethyl-1,4-dihydropyridine-3,5-dicarboxylate or Hantzsch ester (2) as a yellow fine solid in 83% (10.5 g) yield. NMR spectra were fully consistent with reported literature values.[6] ^{1}H NMR (500 MHz, $CDCl_3$): δ_H 5.17 (brs, 1H), 4.16 (q, J = 7.1 Hz, 4H), 3.26 (s, 2H), 2.19 (s, 6H), 1.28 (t, J = 7.1 Hz, 6H) ppm; ^{13}C NMR (125 MHz, $CDCl_3$): δ_C 168.0, 144.7, 99.5, 59.6, 24.8, 19.2, 14.5 ppm.

Notes

- Hantzsch ester is also labile to air and light, so it should be stored in dark-coloured sealed tubes.

3. The manganese complexes catalytic system

A quick look into the manganese catalytic system

The manganese catalytic system has been proposed and thoroughly studied in the past years by Costas and coworkers. Briefly, this catalytic system has successfully been applied among others in the oxidation of aliphatic C-H bonds,[7] the epoxidation of alkenes[8] and the lactonization of nonactivated primary and secondary γ-C–H bonds.[9] In these reactions, H_2O_2 is used as an environmentally benign oxidant to generate electrophilic high-valent metal-oxo species, which react by a mechanism akin to the generally accepted hydrogen atom transfer (HAT)/hydroxyl rebound one displayed by natural metalloenzymes.

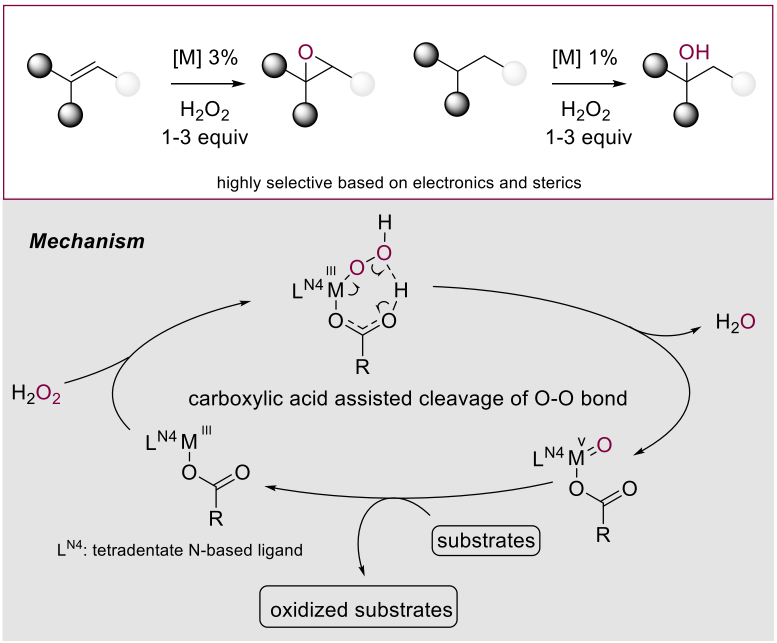

Scheme 2. The manganese catalytic system. Selected examples of C–H Bond Oxidation Promoted by Bioinspired Catalysts. A. Oxidation of aliphatic C-H bonds. B. Epoxidation. C. Lactonization.

3.1 Preparation of the manganese catalysts

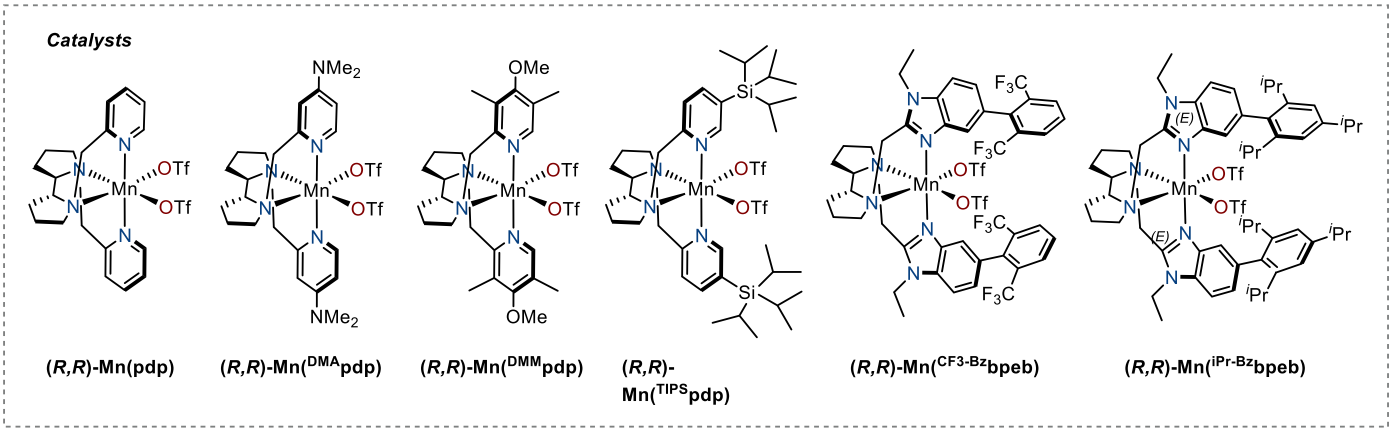

Scheme 3. Structures of the manganese catalysts employed in this work.

All the metal complexes employed in this work were prepared by Costas' group, according to reported procedures. For more details please see: Fe(pdp), Mn(^{TIPS}pdp) & Fe(^{TIPS}pdp),[10] Mn(pdp),[11] Mn(^{DMM}pdp) & Mn(^{DMA}pdp),[12] Mn($^{iPr-Bz}$bpeb) & Mn($^{CF3-Bz}$bpeb).[9]

4. Synergistic Catalysis Method

The thought behind the method

Considering all the achievements of manganese catalysis with the aid of hydrogen peroxide, as well as the DKP catalysis' applications, and encouraged by the ability of DKP (**1**) to coexist with other metal catalysts, we imagined combining the best of both worlds, to present a new synergistic catalysis method for C-H activation. The in situ formed peroxy species, deriving from DKP and manganese catalyst in presence of dioxygen (Scheme 4), would replace hydrogen peroxide leading to a new oxidation protocol, where dioxygen would serve as the sole oxidant of the process.

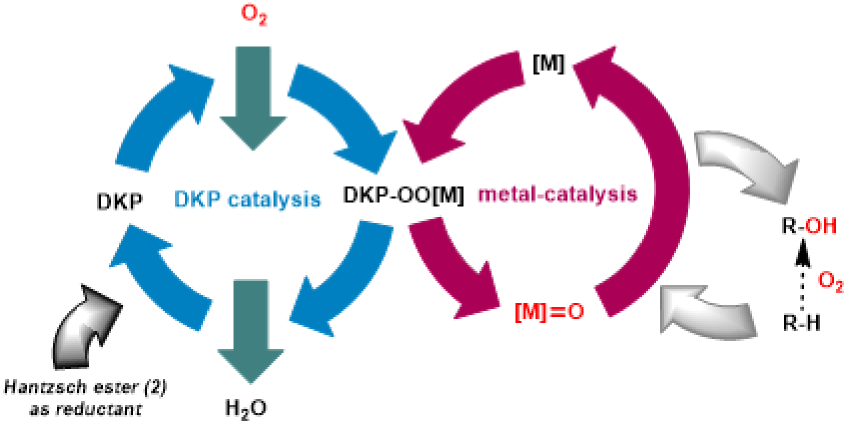

Scheme 4. The thought behind the synergistic method presented.

4.1 Developing the method for CH oxidation: Optimization of reaction conditions

As a starting point, we selected saturated geraniol acetate (**3**) as the model substrate to investigate its aerobic C-H oxidation (Table 1). This molecule has limited and differentiated positions for C-H activation (the proximal and the remote tertiary C-H bonds, as shown in Scheme 5), as well as a relatively low molecular weight, making it suitable for GC analysis but not prone to volatility under the reaction conditions.

After choosing the model substrate and synthesizing it (two steps from the commercially available geraniol, the procedure is described in section 5 of SI), we further proceeded to scanning the reaction profile using manganese catalysis with the aid of hydrogen peroxide. This would help us characterize the oxidation products we should expect and prepare a suitable method for the GC analysis.

*Oxidation of saturated geraniol acetate (**3**) with the aid of hydrogen peroxide*

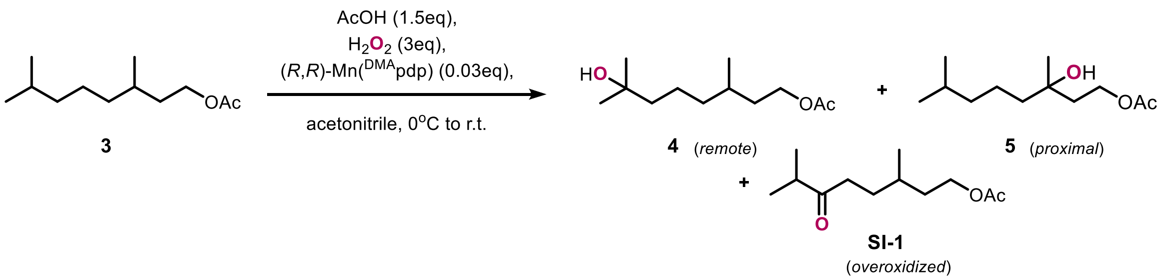

Scheme 5. Screening the oxidation profile of (3) with the aid of hydrogen peroxide. The reaction set up was in accordance with previously reported procedures. [13]

Procedure

A 4ml vial was charged with **3** (23.4 mg, 0.1168 mmol, 1 equiv.) and a magnetic stirring bar. The substrate was diluted to acetonitrile (2 mL). Subsequently, acetic acid (10 μL, 0.1752 mmol, 1.5 equiv.) and (*R,R*)-Mn(^{DMA}pdp) (2.7 mg, 0.0036 mmol, 0.03 equiv.) were added, and the reaction mixture was cooled at 0°C, using an ice bath. Slow addition of H_2O_2 30% (27 μL, 0.2336 mmol, 2 equiv.) followed, via a syringe, over 2h. After syringe addition, the solution was stirred for 10 min at 0°C and at room temperature overnight.

For GC analysis: 30 μL of the crude reaction mixture were filtered through a thin pad of celite, which was further washed with acetonitrile (up to a final volume of 1 mL), and selected to a GC vial.

For isolation of products: The reaction mixture was diluted with EtOAc (5 mL) and extracted with water (5 mL). The aqueous layer was further washed with EtOAc (3x 5 mL). The combined organic layers were dried with Na_2SO_4, filtered and the solvent was removed under reduced pressure. The obtained residue was separated with flash column chromatography on silica-gel, to afford **SI-1**, **4**, **5** and unreacted **3** (fully characterized by NMR analysis, see section 5 of SI).

GC Analysis Report

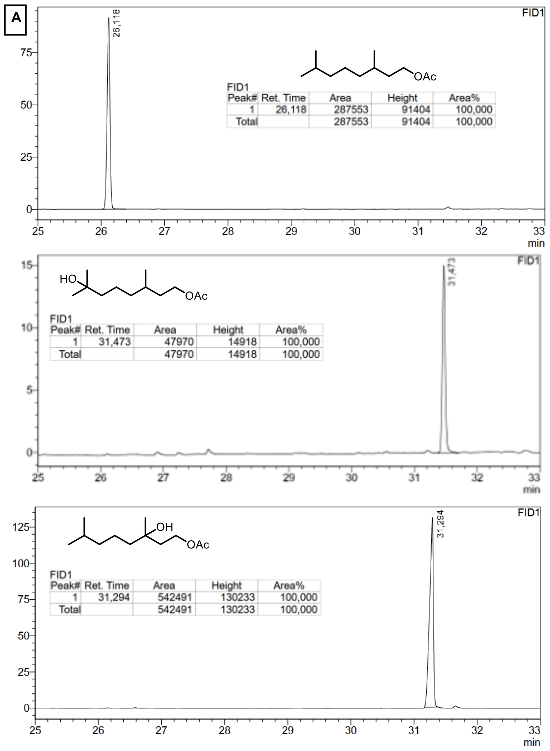

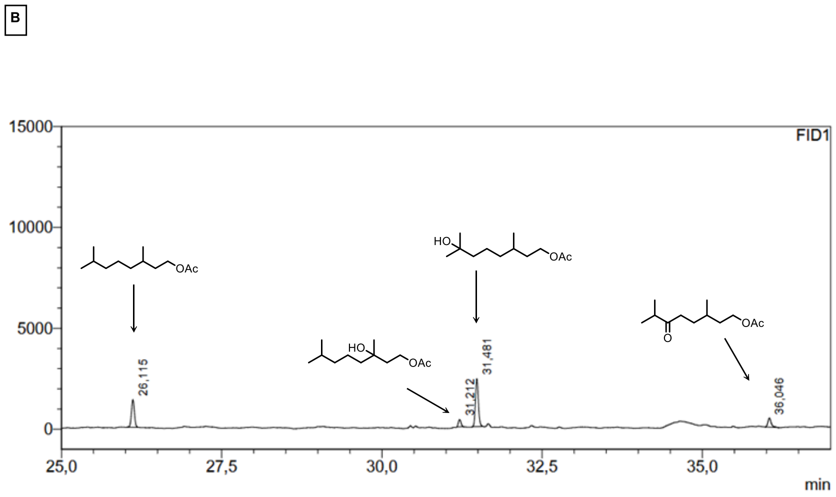

<Peak Table>

FID1

Peak#	Ret. Time	Area	Height	Area%
1	26,115	4404	1360	29,918
2	31,212	1019	365	6,926
3	31,481	7625	2370	51,805
4	36,046	1671	451	11,352
Total		14720	4546	100,000

Scheme 6. GC analysis of the oxidation of model substrate (3). A. Chromatographs of pure starting material and products isolated with column chromatography. B. Crude mixture of the reaction with the aid of hydrogen peroxide.

Results

As expected, two main products were isolated, alcohols **4** and **5**, along with traces of the overoxidized product **SI-1**. Oxidation at the remote C-7 position was detected in a greater extend, probably due to the presence of the electron withdrawing acetyl group. A GC method was prepared and used for monitoring all the optimization reactions: the column temperature was set at 60 °C (equilibration time: 3 min), held isothermally for 4 min, raised to 250 °C with a rate of 6°C/min, and finally held at 250 °C for 25 min. The detector temperature was 250 °C and the temperature at the injector was 250 °C.

Screening of various metal catalysts and blank experiments

Having established the products we should expect, the next step included the introduction of DKP (**1**) catalysis in the picture, which would take the place of hydrogen peroxide. Since the catalytic cycle of this organocatalyst has been thoroughly studied before,[1] we sought to exploit these optimized conditions for our first attempts. That was employing 10 mol% of DKP organocatalyst (**1**) and a stoichiometric amount of Hantzsch ester reductant (**2**) in HFIP, in the presence of dioxygen, along with 1 mol% of various redox-active complexes, all in one portion.

Procedure

A 4ml vial was charged with **3** (10 mg, 0.05 mmol, 1equiv.) and a magnetic stirring bar. The substrate was diluted to HFIP (2 mL). Subsequently, HE (12.7 mg, 0.05 mmol, 1equiv.), DKP (0.95 mg, 0.005 mmol, 0.1eq) and the redox active metal (0.0005 mmol, 0.01 equiv.) were added. The vial was tightly capped by a rubber septum. An exit needle and a pipette were introduced in the septum, through which dioxygen was bubbled continuously by a balloon. Care was taken to adjust slow bubbling in the reaction vial to avoid solvent's evaporation. The reaction mixture was stirred at room temperature, under dioxygen atmosphere, overnight.
For GC analysis: 60-90 μL of the crude reaction mixture were filtered through a thin pad of celite, which was further washed with acetonitrile (up to a final volume of 1 mL), and selected to a GC vial.
For isolation of products: The reaction mixture was evaporated under reduced pressure and the residue was diluted with EtOAc (3 mL) and extracted with water (3 mL). The aqueous layer was further washed with EtOAc (3x3mL). The combined organic layers were dried with Na_2SO_4, filtered and the solvent was removed under reduced pressure. ^{1}HNMR analysis and column chromatography followed for qualitative analysis, while quantitative analysis was performed with GC.

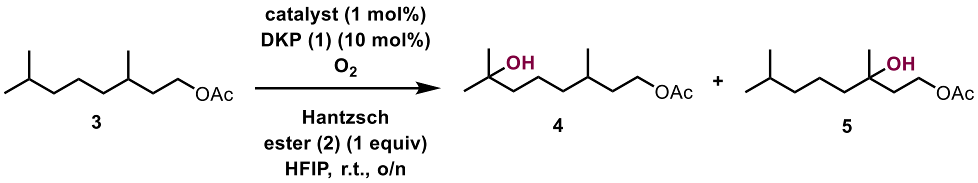

Entry[a]	Catalyst	4; 5 (yields%)
1[b]	-	0%; 0%
2	*(R,R)*-Fe(pdp)	0%; 0%
3	*(R,R)*-Fe(^{TIPS}pdp)	0.6%; 0%
4	(S,*S*)-Mn(^{DMM}pdp)	1.9%; 0.2%
5	*(R,R)*-Mn(^{DMA}pdp)	2.8%; 0.3%
6	*(R,R)*-Mn(^{TIPS}pdp)	3.9%; 0.2%
7	*(S,S)*-Mn(^{TIPS}pdp)	3.1%; 0.1%
8[c]	*(R,R)*-Mn(^{TIPS}pdp)	0%; 0%
9[d]	*(R,R)*-Mn(^{TIPS}pdp)	1.6%; 0.2%
10	*(R,R)*-Mn($^{iPr-Bz}$bpeb)	2.2%; 0.5%
11	*(S,S)*-Mn(pdp)	1.0%; 0.1%

Table 1. Screening of various catalysts for aerobic oxidation of **3** in the presence of DKP catalysis. Conditions used: substrate (0.05 mmoles), catalyst (1 mol%), DKP (10 mol%), Hantzsch ester (0.05 mmoles), HFIP (2 ml), rt stirring overnight with dioxygen bubbling; [b]Reaction was performed in the same conditions without addition of a redox-active catalyst. [c]The reaction was performed in the absence of DKP and Hantzsch ester. [d]AcOH (10 mol%) was added in the reaction mixture.

GC Analysis Reports

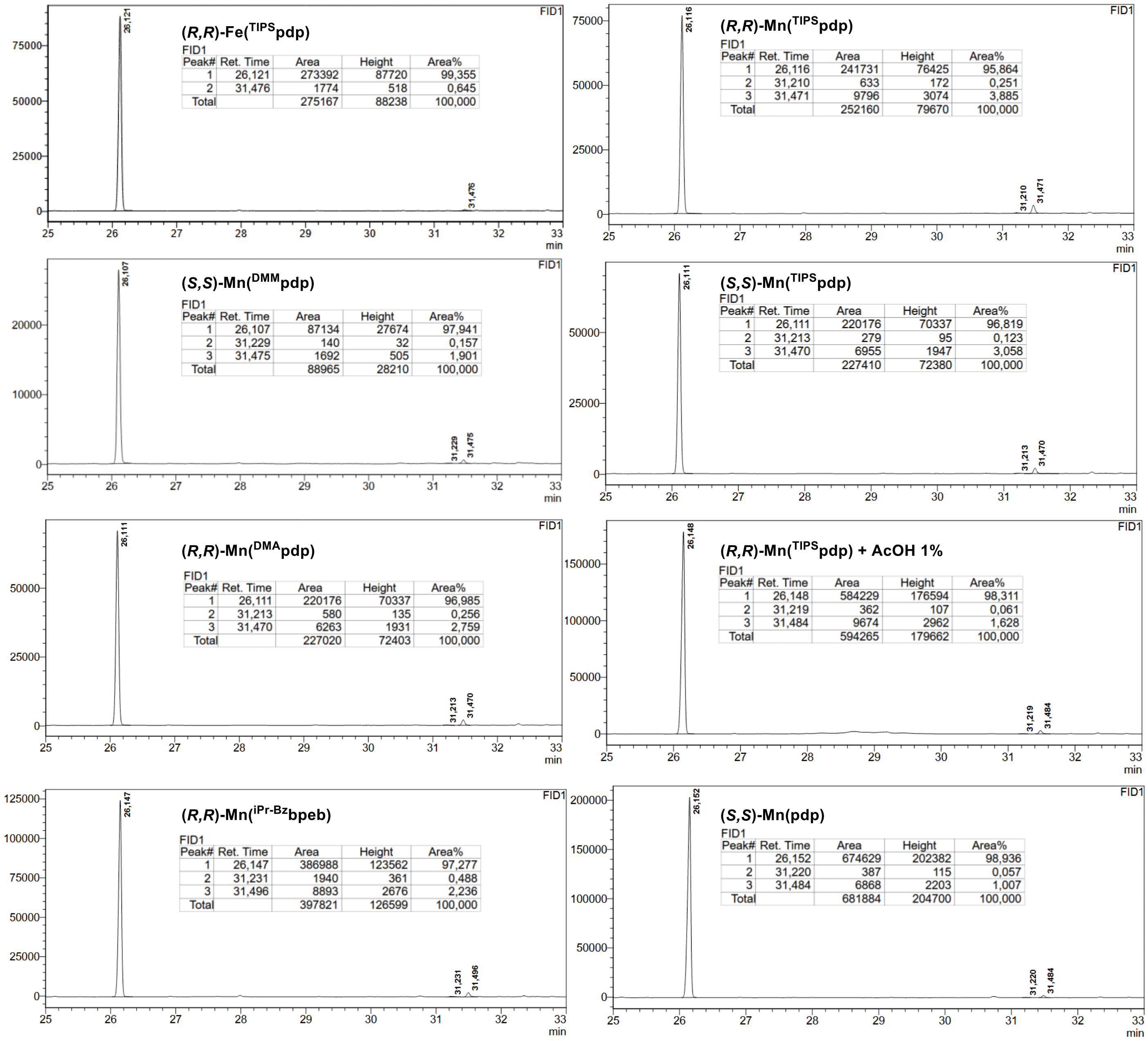

Scheme 7. GC chromatographs of crude reactions for screening of various catalysts. For conditions used please advise table 1. Retention times: for starting material **3** t = 26.11 min, product **4** t = 31.47 min, product **5** t = 31.29 min. Product **SI-1** was not observed.

Results

Regarding the blank experiments, lacking either DKP (**1**), Hantzsch ester (**2**), or redox-active metal complexes, as expected, no activation was observed (Table 1; entries 1 and 8). Similarly, no oxidation or traces of products were observed when iron catalysts were combined with DKP catalysis (Table 1; entries 2-3). However, when manganese catalysts were tested in combination with aerobic DKP catalysis, GC analysis revealed yields ranging from 1.0-3.9% for the 7-hydroxylated product **4** along with traces of the 3-hydroxylated congener **5** (Table 1). The best yield was delivered with catalyst (*R*,*R*)-Mn(^{TIPS}pdp) (entry 6)**,** while utilizing its enantiomer (entry 7) led to a slight decrease of the yield detected. In all cases, overoxidized product **SI-1** was not observed. Its absence was attributed to the use of HFIP as solvent (in contrast with acetonitrile which was used in the reaction with the aid of hydrogen peroxide). Even though the overall yields were extremely low, (*R*,*R*)-Mn (^{TIPS}pdp) showed the most prominent results.

Screening of different solvents

Having established the superiority of (*R,R*)-Mn (^{TIPS}pdp) among the metal catalysts tested, we further proceeded in testing different solvents for the reaction. Considering the sensitivity of both the metal and the DKP catalysts, our experimentations were limited to solvents already tested in each method, like DMF and DCM (previously used in DKP catalysis) as well as TFE and acetonitrile (broadly used in manganese catalysis). Unfortunately, neither of these modifications improved the yield of product **3** detected. DMF totally halted the oxidation, probably due to the rapid oxidation of the DKP catalyst (**2**), while 1:3 DCM/HFIP mixture, acetonitrile and TFE dramatically reduced the formation of **3**.

Screening of different reductants

Taking into consideration the competitive oxidation of Hantzsch ester, to its corresponding pyridine, by the manganese complexes, we sought to try replacing (**2**) with a different reductant. Once again, finding a reagent compatible with both the DKP cycle and the manganese one proved tricky. Inspired by monooxygenases in Nature, we tried to mimic the reductase enzyme, utilizing NADH as a reductant, unfortunately with no success. Ascorbic acid and $Na[BH_3(CN)]$ were also tested, leading to nearly zero activation, while reactions with Zn^0 as a reductant met the same fate. Thus, we concluded that there was no obvious replacement for the Hantzsch ester, so we decided to continue our experimentation leaving this part untacked.

Reaction profile analysis and characterization of reaction intermediates

To improve the process, we sought to understand the underlying factors governing this aerobic transformation. The first thing we should rule out was a possible complexation between the manganese catalyst and the DKP organocatalyst under the reaction conditions, which would prematurely terminate the oxidation process. To do so, the components (DKP and (*R*,*R*)-Mn(^{TIPS} pdp)) were allowed to stir in stoichiometric quantities in HFIP, for several hours without observable reactivity (neither the colour of the solution nor the TLC changed or ^{1}HNMR).

In the meantime, we observed that in every examined case (as described in *Screening of various metal catalysts and blank experiments s*ection), proton NMR analysis at the end of the reaction revealed the absence of Hantzsch ester (**2**) and the exclusive formation of Hantzsch pyridine **6**, alongside with unreacted **3** as well as species related to DKP decomposition. For further insights, we isolated and identified them to be the hemiaminal **8**, amide **7** and acid **9** (Scheme 8), which indicates the complete oxidative decomposition of DKP under the reaction conditions.

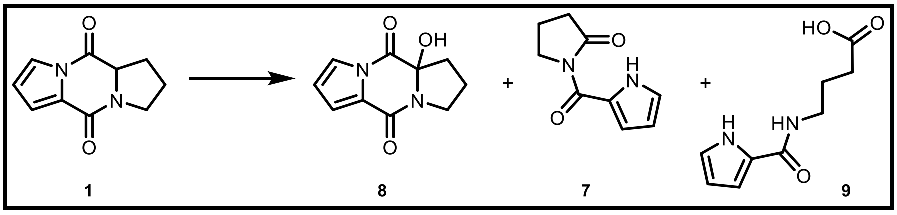

Scheme 8. DKP decomposition under the reaction conditions, after full consumption of HE (**2**).

To further study the metal-induced oxidative decomposition of DKP, a kinetic experiment was conducted, using 0.5 equivalents of DKP, 10 mol% of (*R*,*R*)-Mn(^{TIPS}pdp), and 1 equivalent of Hantzsch ester and stirring under dioxygen in the absence of substrate. The ^{1}HNMR analysis revealed that throughout the oxidation of Hantzsch ester to the corresponding pyridine, no oxidation or decomposition of DKP-H **1** is observed (Spectra 1). When Hantzsch ester was fully consumed, a rapid decomposition of DKP-H is apparent indicating the synergistic effect of Hantzsch pyridine in the acceleration of DKP-H oxidation in HFIP (Spectra 3).

Finally, a kinetic experiment was conducted to evaluate the effect of metal catalysis on Hantzsch ester aerobic oxidation in the absence of the DKP catalyst (Scheme 9). The results demonstrated a significant acceleration in the oxidation of Hantzsch ester (**2**) to Hantzsch pyridine (**6**), which was quantitatively formed in under an hour, compared to several hours without metal catalysis under dioxygen and HFIP conditions. This oxidation is attributed to electron donation from Hantzsch ester to a steady-state manganese (III) complex, resulting in the formation of Hantzsch pyridine and manganese (II) complex. This pathway is postulated to drive the rapid depletion of Hantzsch ester and, consequently, the oxidative decomposition of DKP, ultimately shutting down the entire process.

Spectra 1

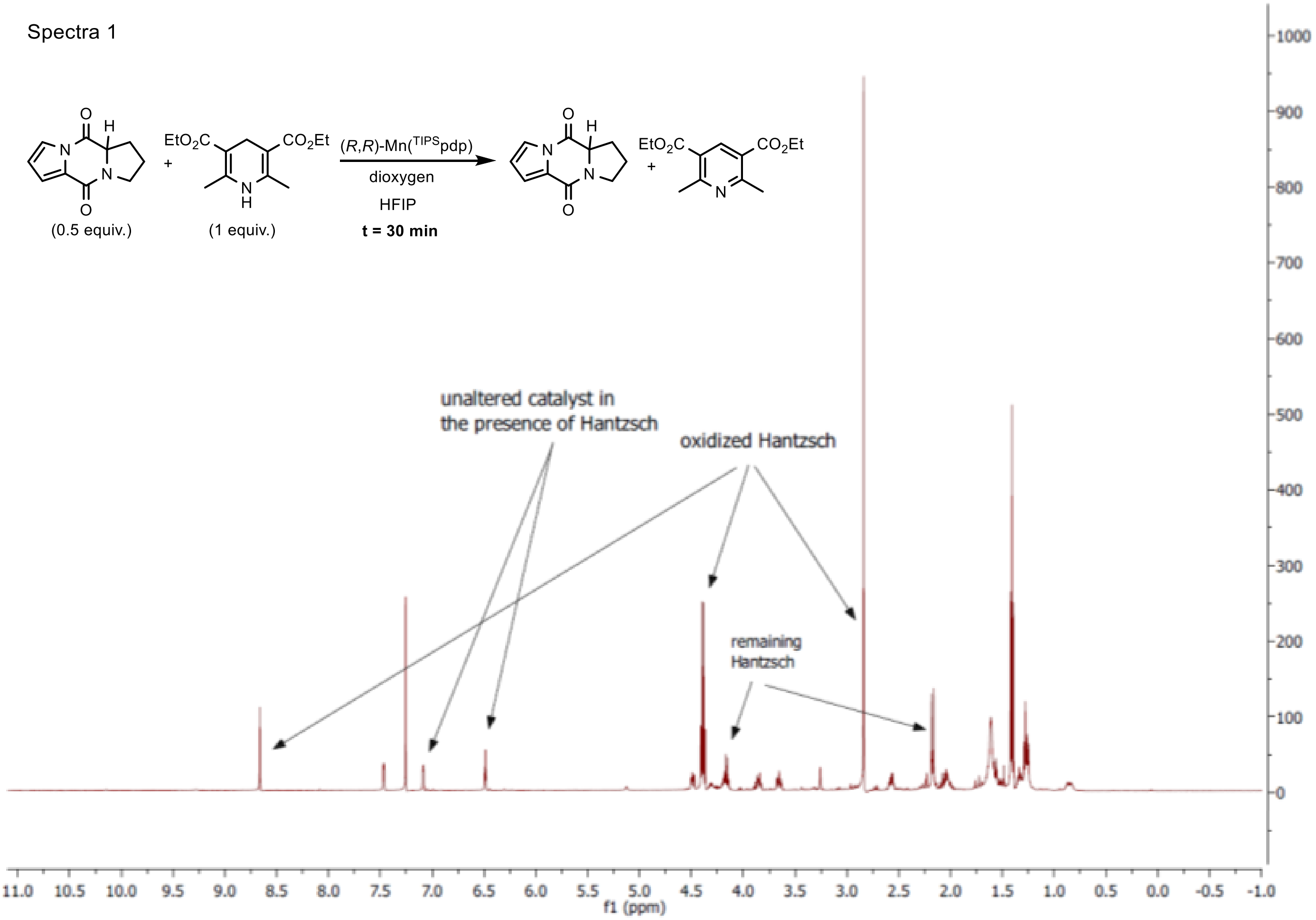

Spectra 2

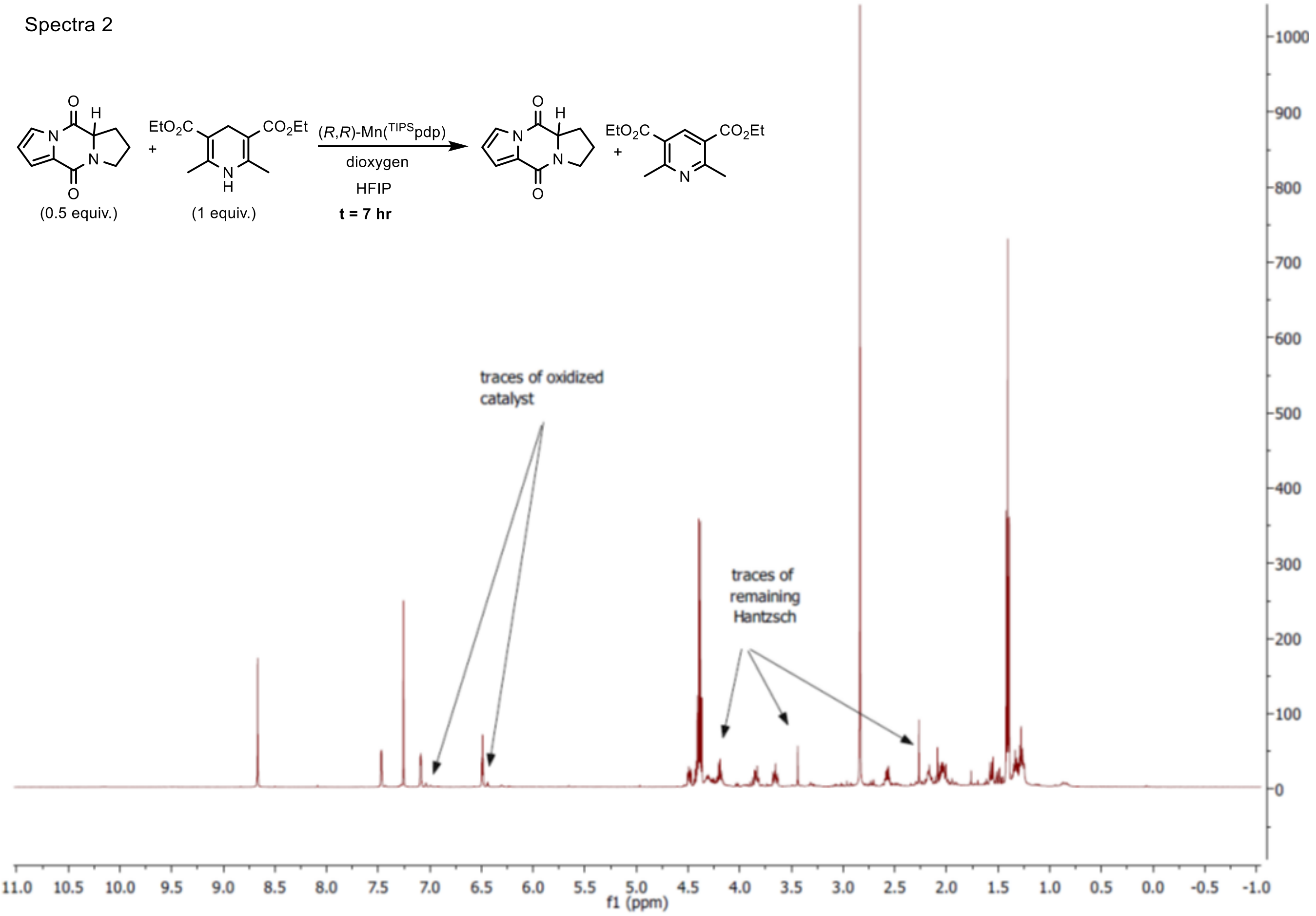

Spectra 3

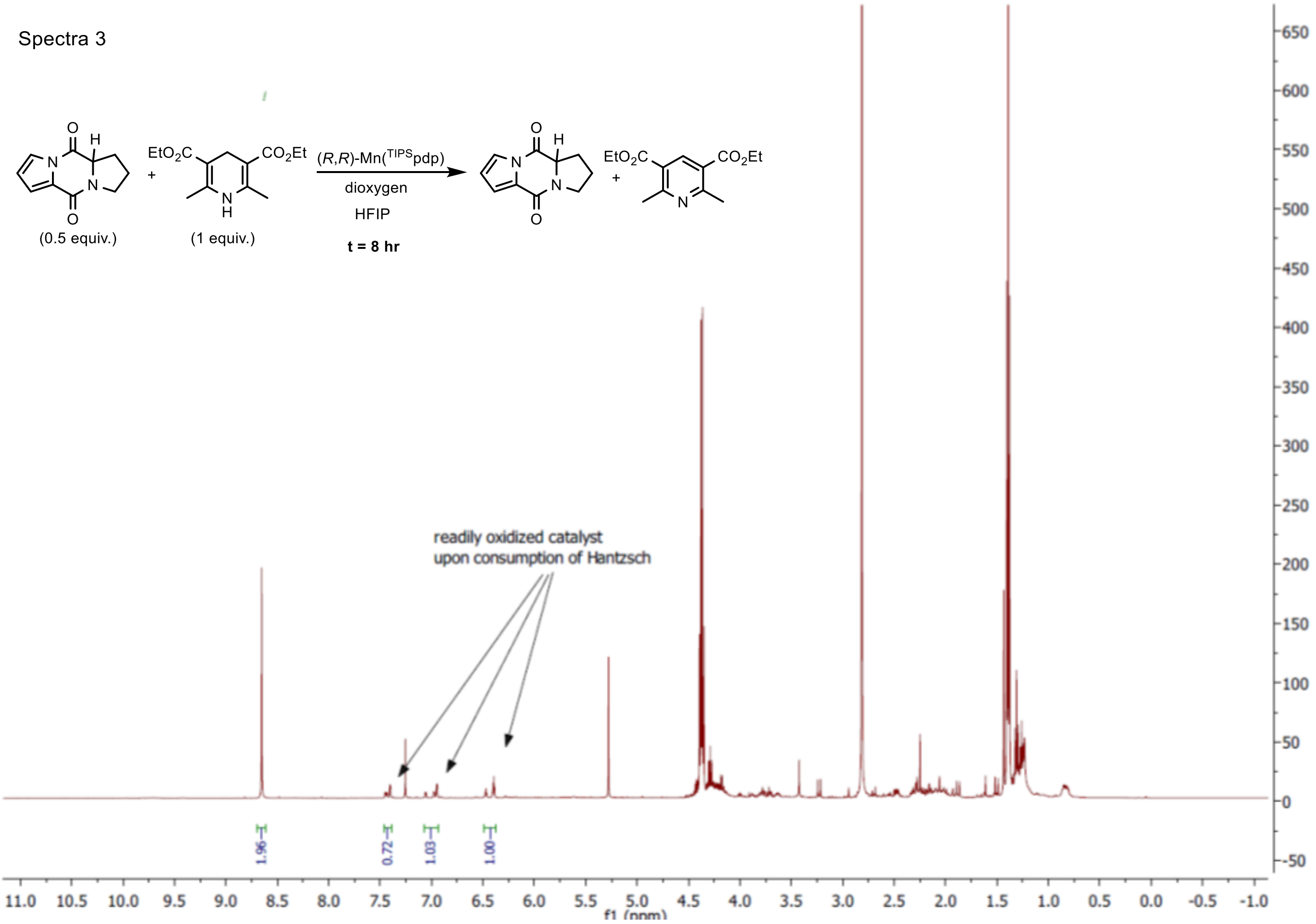
(0.5 equiv.)
(1 equiv.)
(R,R)-Mn(TIPSpdp)
dioxygen
HFIP
t = 8 hr
readily oxidized catalyst
upon consumption of Hantzsch
1.96
0.72
1.03
1.00
f1 (ppm)

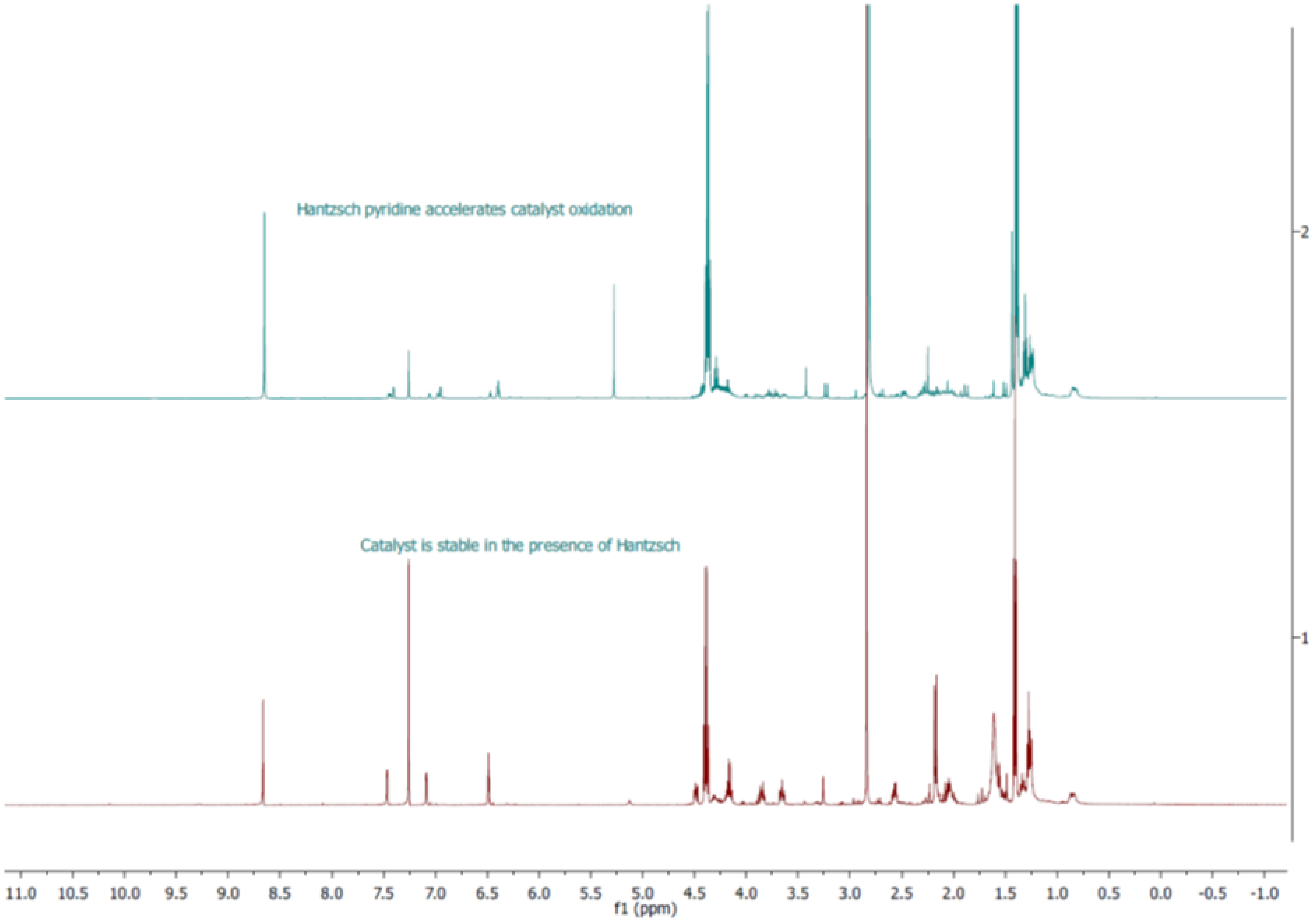
Hantzsch pyridine accelerates catalyst oxidation
Catalyst is stable in the presence of Hantzsch
f1 (ppm)

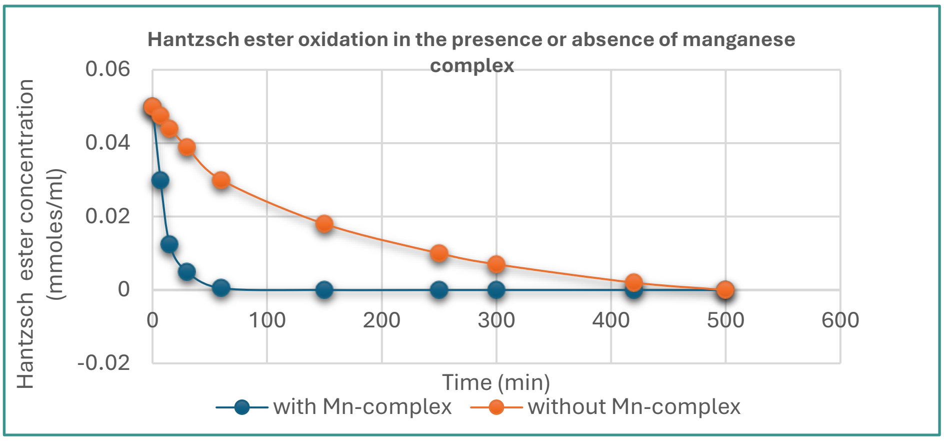

Scheme 9. Rate of oxidation of HE (**2**) in the presence or absence of manganese catalyst.

Testing the effect of catalyst bulkiness and Hantzsch ester's steady supply

At this point, it became clear that since we could not replace (**2**), we should find a way to either retard its oxidation or retain its steady supply in the reaction mixture. Thus, two different pathways were dictated. Increasing manganese catalyst's bulkiness could potentially delay the electron donation from Hantzsch ester to the metal centre, slowing down its oxidation. For this reason, a bulkier manganese catalyst was tested (Table 2; entries 2 and 5). Meanwhile, to have a steady supply of (**2**) in the reaction mixture, we sought to initially increase its amount (Table 2; entries 1 and 2) and later use a syringe pump for its addition (Table 2; entries 3-8).

Procedure

For entries 1-2 of table 2

A 4ml vial was charged with **3** (10 mg, 0.05 mmol, 1equiv.) and a magnetic stirring bar. The substrate was diluted to HFIP (2 mL). Subsequently, HE (xx mg, xx mmol, xx equiv.), DKP (0.95 mg, 0.005 mmol, 0.1eq) and the redox active metal (0.0005 mmol, 0.01 equiv.) were added. The vial was tightly capped by a rubber septum. An exit needle and a pipette were introduced in the septum, through which dioxygen was bubbled continuously by a balloon. Care was taken to adjust slow bubbling in the reaction vial to avoid solvent's evaporation. The reaction mixture was stirred at room temperature, under dioxygen atmosphere, overnight.

For entries 3-8 of table 2

A 4ml vial was charged with **3** (10 mg, 0.05 mmol, 1equiv.) and a magnetic stirring bar. The substrate was diluted to HFIP (2 mL). Subsequently, HE (25.3 mg, 0.1 mmol, 2 equiv.), DKP (0.95 mg, 0.005 mmol, 0.1eq) and the redox active metal (0.0005 mmol, 0.01 equiv.) were added. The vial was tightly capped by a rubber septum. An exit needle and a pipette were introduced in the septum, through which dioxygen was bubbled continuously by a balloon. Care was taken to adjust slow bubbling in the reaction vial to avoid solvent's evaporation. Also, a syringe charged with a solution of HE (**2**) in HFIP (xx mg, xx mmol, xx ml) was adjusted to the septum, the other edge of which was introduced to a syringe pump (the remaining HE equiv. are added as 0.07M solution in HFIP in such rate to achieve the indicated times of Table 2). The reaction mixture was stirred at room temperature, under dioxygen atmosphere, overnight.

For GC analysis: 60-90 μL of the crude reaction mixture were filtered through a thin pad of celite, which was further washed with acetonitrile (up to a final volume of 1mL), and selected to a GC vial.
For isolation of products: The reaction mixture was evaporated under reduced pressure and the residue was diluted with EtOAc (3 mL) and extracted with water (3 mL). The aqueous layer was further washed with EtOAc (3x3mL). The combined organic layers were dried with Na_2SO_4, filtered and the solvent was removed under reduced pressure. ^{1}HNMR analysis and column chromatography followed for qualitative analysis, while quantitative analysis was performed with GC.

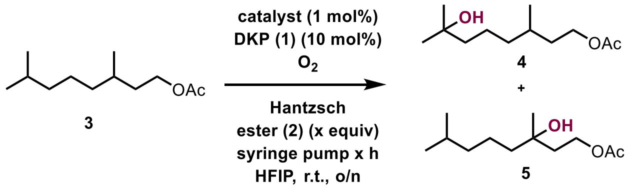

Entry	HE (equiv.) in addition time	Catalyst	4; 5 (yields%)
**1**	5 one portion	(*R*,*R*)-Mn(^{TIPS}pdp)	3.7%; 0.3%
**2**	5 one portion	(*R*,*R*)-Mn($^{iPr-Bz}$bpeb)	2.3%; 0.5%
**3**	5 in 5h	(*R*,*R*)-Mn(^{TIPS}pdp)	4.5%; 0.4%
**4**^{b}	10 in 5h	(*R*,*R*)-Mn(^{TIPS}pdp)	6.9%; 0.7%
**5**^{b}	10 in 5h	(*R*,*R*)-Mn($^{iPr-Bz}$bpeb)	5.5%; 1.0%
**6**^{b}	10 in 5h	(*R*,*R*)-Mn(pdp)	2.1%; 0.2%
**7**	10 in 10h	(*R*,*R*)-Mn(^{TIPS}pdp)	9.4%; 0.7%
**8**	10 in 20h	(*R*,*R*)-Mn(^{TIPS}pdp)	6.8%; 0.7%

Table 2. Testing the effect of catalyst bulkiness and Hantzsch ester's steady supply. Conditions used: substrate (0.05 mmoles), catalyst (1 mol%), DKP (10 mol%), Hantzsch ester = HE equiv, HFIP (2 ml), rt stirring overnight with dioxygen bubbling. For syringe pump additions an initial portion of 2 equiv. HE in reaction mixture is added. The remaining HE equiv. are added as 0.07M solution in HFIP in such rate to achieve the indicated times of Table 2. ^{b}HE concentration to (0.14M).

GC Analysis Reports

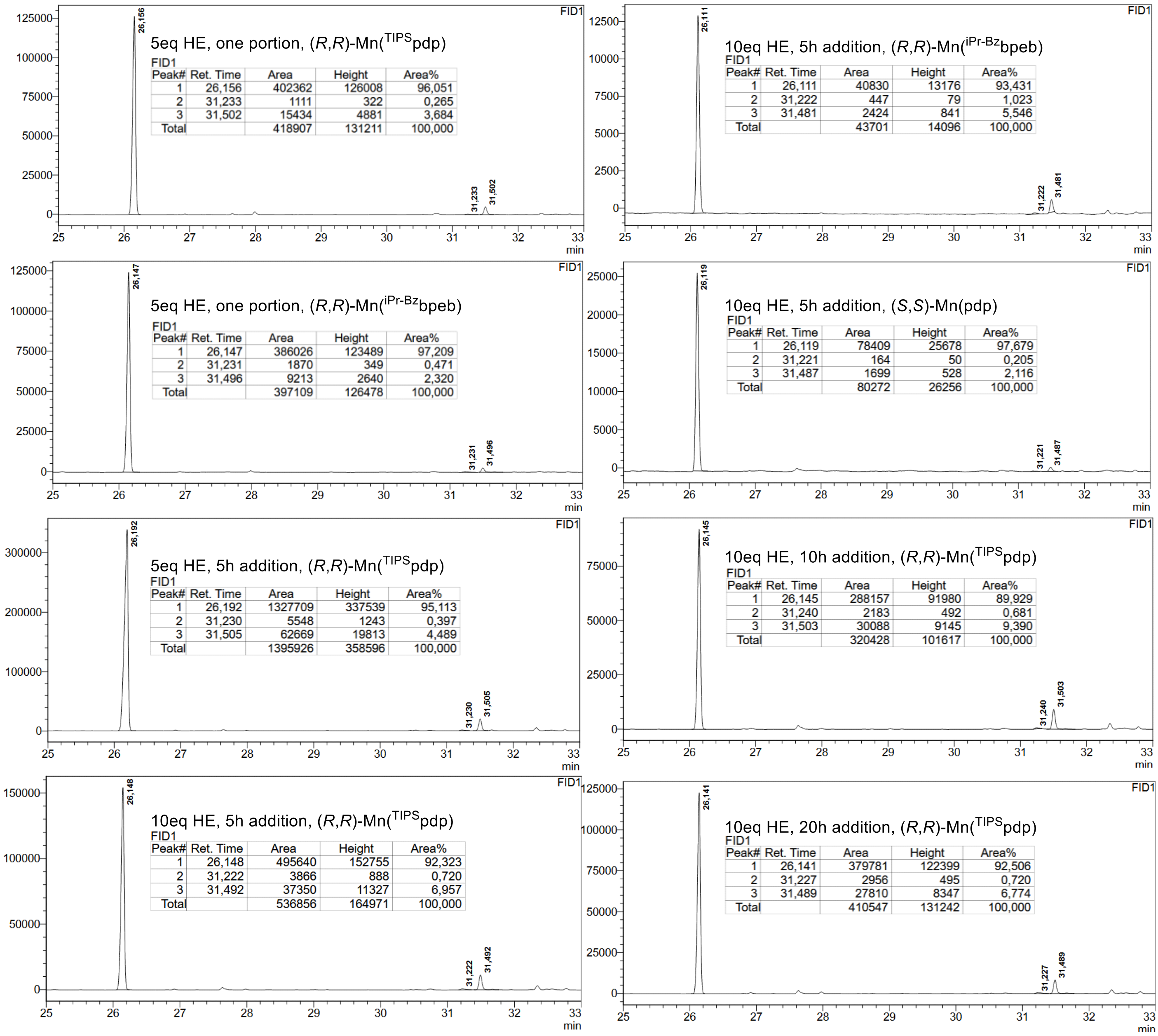

Scheme 10. GC chromatographs of crude reactions for testing the effect of catalyst bulkiness and Hantzsch ester's steady supply. For conditions used please advise table 2.

Results

Increasing the amount of Hantzsch ester (**2**) to 5 equivalents, in the presence of 10 mol% DKP (**1**) and 1 mol% (*R*,*R*)-Mn(^{TIPS}pdp) catalyst, resulted in a modest increase in the reaction yield to 4% for the C-H oxidation products 4 and 5 (Table 2; entry 1). The key question was whether this yield improvement was due to the higher concentration of Hantzsch ester or its prolonged presence in the reaction mixture. To investigate further, a syringe pump was employed for the gradual addition of Hantzsch ester. Supplying the same equivalents of Hantzsch ester dissolved in HFIP (0.07 M) over 5 hours, while keeping all other parameters constant, led to a slightly improved yield of 5.1% (Table 2; entry 3). Increasing the Hantzsch ester to 10 equivalents, while maintaining the 5-hour addition time (corresponding to a higher steady-state concentration of 0.014 M in the reaction vessel), further optimized the yield to 7.5% (Table 2; entry 4).

Different addition times were also evaluated (Table 2; entries 6–7), with a 10-hour addition at 0.07 M proving most effective, achieving a yield of 10.5% for oxidized products 4 and 5. Notably, (*R*,*R*)-Mn(^{TIPS}pdp) consistently outperformed the bulkier (*R*,*R*)-Mn($^{iPr\text{-}Bz}$bpeb) under these optimized conditions (Table 2; entries 5 and 6). In all cases, good selectivity between the 7-hydroxylated and 3-hydroxylated products was observed. Interestingly, no additional products arising from methylene oxidation were detected. Although the yields were comparable

between the two catalysts, the reduced regioselectivity observed for (*R,R*)-Mn($^{iPr-Bz}$bpeb) (>10:1 for (*R,R*)-Mn(^{TIPS}pdp) vs. 6:1 for (*R,R*)-Mn($^{iPr-Bz}$bpeb)) ultimately led to its exclusion from further investigation.

Effect of acidic additives in premixing conditions with the catalyst

Hoping to further improve the method, we decided to add to the equation another parameter we, until that time, ignored. That was incorporating an acidic additive that has proven to contribute to the rapid formation of oxo-metal intermediates. Two main problems could arise from such a modification: firstly, the observed incompatibility of acids with Hantzsch ester (the presence of an acid accelerates the oxidation of (**2**) to the corresponding pyridine), and secondly the halt of DKP's cycle (excess of acid reduces the efficacy of the oxidation step of (**1**)).[1] To avoid the direct contact of acid with Hantzsch ester, we considered the premixing of (*R,R*)-Mn(^{TIPS}pdp) catalyst with various acidic additives prior to its addition to the reaction mixture (Table 3). To prevent the interference of acid to DKP's cycle, we maintained the quantities of acid tested up to 10% maximum.

Procedure

A 2ml vial was charged with manganese catalyst (xx mg, 0.005 mmol, 0.01 equiv.) and a magnetic stirring bar. HFIP (0.1 ml) was added, along with the indicated acid (xx mg, xx mmol, xx equiv.) and the solution was left stirring, at room temperature, for 10 minutes (solution **S1**). A 4ml vial was charged with **3** (10 mg, 0.05 mmol, 1equiv.) and a magnetic stirring bar. The substrate was diluted to HFIP (2 mL). Subsequently, HE (25.3 mg, 0.1 mmol, 2 equiv.), DKP (0.95 mg, 0.005 mmol, 0.1eq) and **S1** were added. The vial was tightly capped by a rubber septum. An exit needle and a pipette were introduced in the septum, through which dioxygen was bubbled continuously by a balloon. Care was taken to adjust slow bubbling in the reaction vial to avoid solvent's evaporation. Also, a syringe charged with a solution of HE (**2**) in HFIP (101.3 mg, 0.4 mmol, 8 equiv. in 6 ml) was adjusted to the septum, the other edge of which was introduced to a syringe pump (the addition was adjusted in such rate to achieve the indicated times of Table 3). The reaction mixture was stirred at room temperature, under dioxygen atmosphere, overnight.
For GC analysis: 60-90 μL of the crude reaction mixture were filtered through a thin pad of celite, which was further washed with acetonitrile (up to a final volume of 1 mL), and selected to a GC vial.
For isolation of products: The reaction mixture was evaporated under reduced pressure and the residue was diluted with EtOAc (3 mL) and extracted with water (3 mL). The aqueous layer was further washed with EtOAc (3x3mL). The combined organic layers were dried with Na_2SO_4, filtered and the solvent was removed under reduced pressure. ^{1}HNMR analysis and column chromatography followed for qualitative analysis, while quantitative analysis was performed with GC.

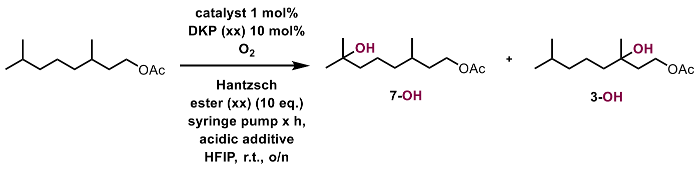

Entry[a]	Addition Time (h)	Catalyst (1 mol%)	Acid (x mol%)	4; 5 (yields %)
1	5	(*R,R*)-Mn(^{TIPS}pdp)	AA 1%	8.2%; 1.0%,
2	5	(*R,R*)-Mn(^{TIPS}pdp)	AA 3%	8.7%; 1.3%
3	5	(*R,R*)-Mn($^{iPr-Bz}$bpeb)	AA 1%	4.2%; 0.6%,
4	5	(*R,R*)-Mn(^{TIPS}pdp)	PA 1%	9.6%; 1.3%,
5	5	(*R,R*)-Mn(^{TIPS}pdp)	PA 3%	11.2%; 0.7%,
6	5	(*R,R*)-Mn(^{TIPS}pdp)	PA 10%	8.9%; 1.3%,
7	5	(*R,R*)-Mn($^{iPr-Bz}$bpeb)	PA 1%	5.5%; 0.9%,
8	10	(*R,R*)-Mn(^{TIPS}pdp)	PA 1%	18.9%; 1.1%,
9	10	(*R,R*)-Mn(^{TIPS}pdp)	PA 3%	17.5%; 1.5%
10	15	(*R,R*)-Mn(^{TIPS}pdp)	PA 1%	9.9%; 0.5%,
11	5	(*R,R*)-Mn($^{o,p-tBu-Bz}$pdp)	PA 1%	4.0%; 0.3%,
12	10	(*R,R*)-Mn(^{TIPS}pdp)	Tf 1%	10.5%; 0.7%,
13[b]	10	(*R,R*)-Mn(^{TIPS}pdp)	PA 1%	28.7%; 2.6%

Table 3. Effect of acidic additive in premixing conditions with the catalyst. Conditions used unless otherwise noticed: substrate (0.05 mmoles), (*R,R*)-Mn(^{TIPS}pdp) (1 mol%) was premixed with the indicated acid for 10 min before its addition to reaction mixture, DKP (10 mol%), Hantzsch ester (10 equiv.) addition in x hours as indicated in Table, HFIP (6 ml), rt stirring overnight under dioxygen bubbling or by saturating oxygen in a septum sealed vial. [b](*R,R*)-Mn($^{iPr-Bz}$bpeb) was used. [c](*R,R*)-

Mn($^{o,p\text{-tBu-Bz}}$pdp) was used. ^{d}An additional portion of DKP (10 mol%) and premixed catalyst-PA (1 mol%) was added after 5 hours of reaction. AA = acetic acid; PA = pivalic acid; Tf = triflic acid

GC Analysis Reports

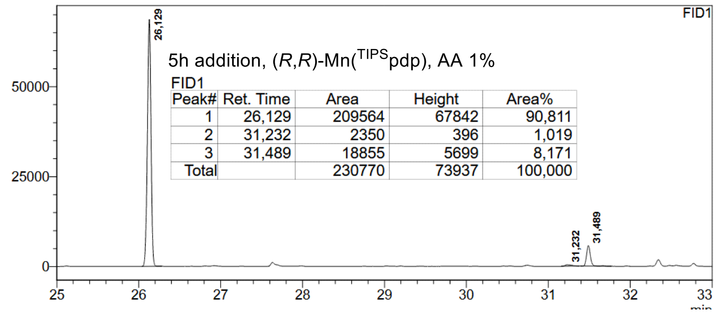

Peak#	Ret. Time	Area	Height	Area%
1	26,129	209564	67842	90,811
2	31,232	2350	396	1,019
3	31,489	18855	5699	8,171
Total		230770	73937	100,000

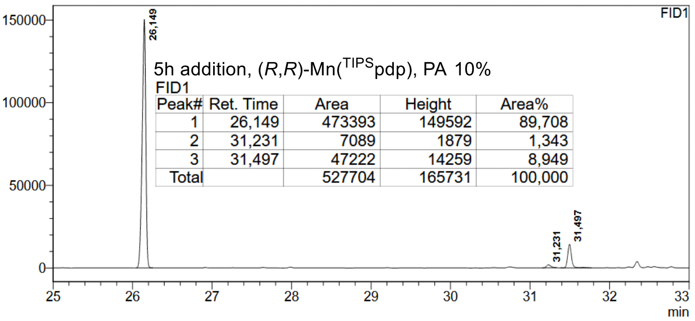

Peak#	Ret. Time	Area	Height	Area%
1	26,149	473393	149592	89,708
2	31,231	7089	1879	1,343
3	31,497	47222	14259	8,949
Total		527704	165731	100,000

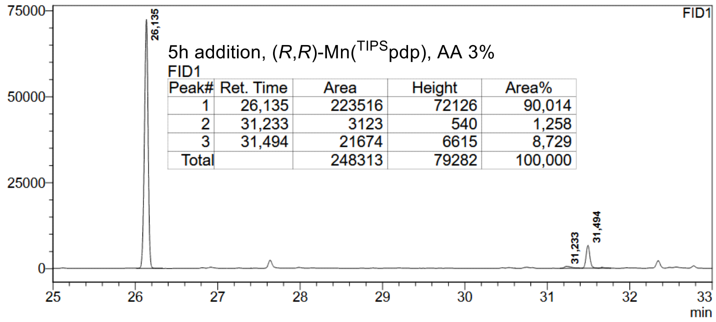

Peak#	Ret. Time	Area	Height	Area%
1	26,135	223516	72126	90,014
2	31,233	3123	540	1,258
3	31,494	21674	6615	8,729
Total		248313	79282	100,000

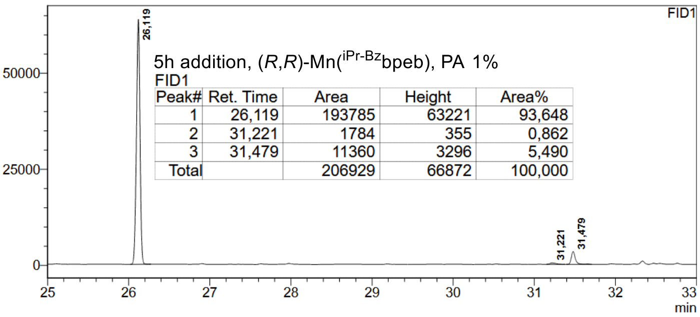

Peak#	Ret. Time	Area	Height	Area%
1	26,119	193785	63221	93,648
2	31,221	1784	355	0,862
3	31,479	11360	3296	5,490
Total		206929	66872	100,000

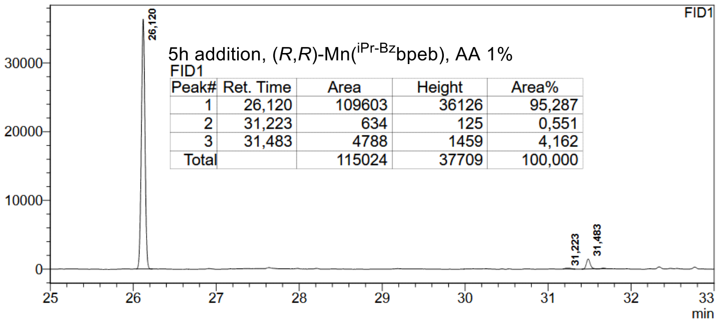

Peak#	Ret. Time	Area	Height	Area%
1	26,120	109603	36126	95,287
2	31,223	634	125	0,551
3	31,483	4788	1459	4,162
Total		115024	37709	100,000

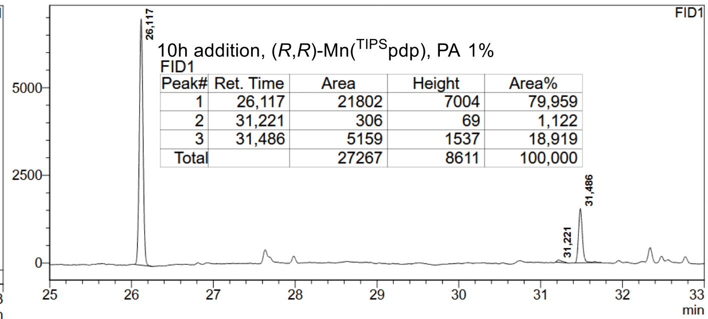

Peak#	Ret. Time	Area	Height	Area%
1	26,117	21802	7004	79,959
2	31,221	306	69	1,122
3	31,486	5159	1537	18,919
Total		27267	8611	100,000

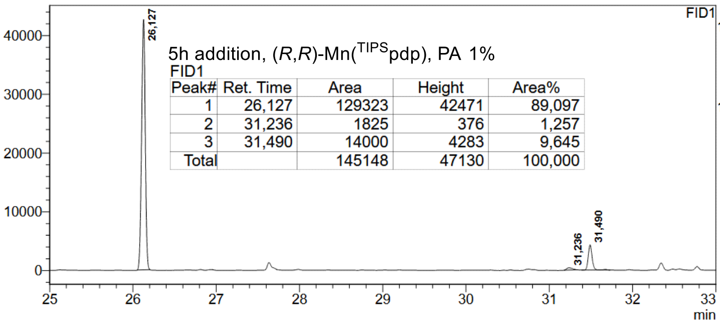

Peak#	Ret. Time	Area	Height	Area%
1	26,127	129323	42471	89,097
2	31,236	1825	376	1,257
3	31,490	14000	4283	9,645
Total		145148	47130	100,000

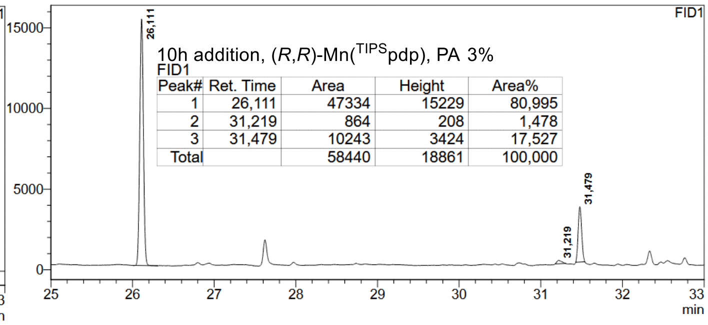

Peak#	Ret. Time	Area	Height	Area%
1	26,111	47334	15229	80,995
2	31,219	864	208	1,478
3	31,479	10243	3424	17,527
Total		58440	18861	100,000

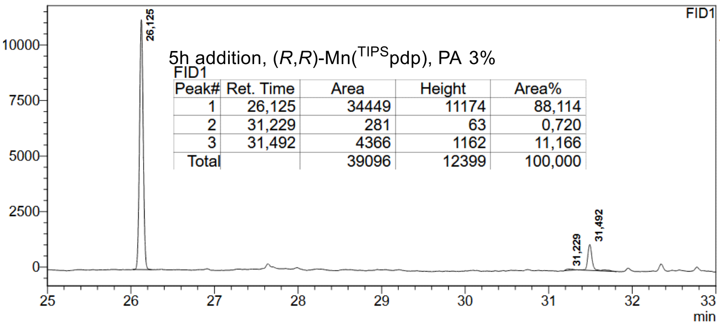

Peak#	Ret. Time	Area	Height	Area%
1	26,125	34449	11174	88,114
2	31,229	281	63	0,720
3	31,492	4366	1162	11,166
Total		39096	12399	100,000

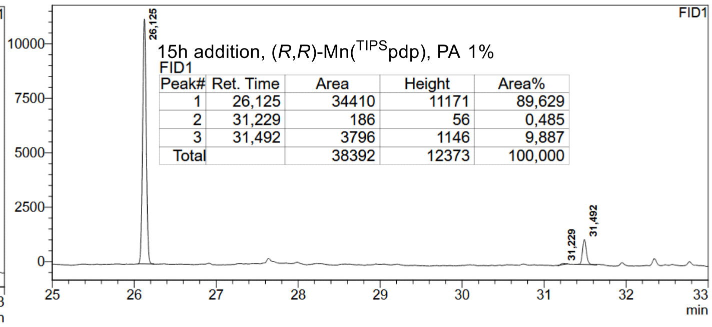

Peak#	Ret. Time	Area	Height	Area%
1	26,125	34410	11171	89,629
2	31,229	186	56	0,485
3	31,492	3796	1146	9,887
Total		38392	12373	100,000

Scheme 11a. GC chromatographs of crude reactions for testing the effect of acidic additive in premixing conditions with the catalyst. For conditions used please advise table 3.

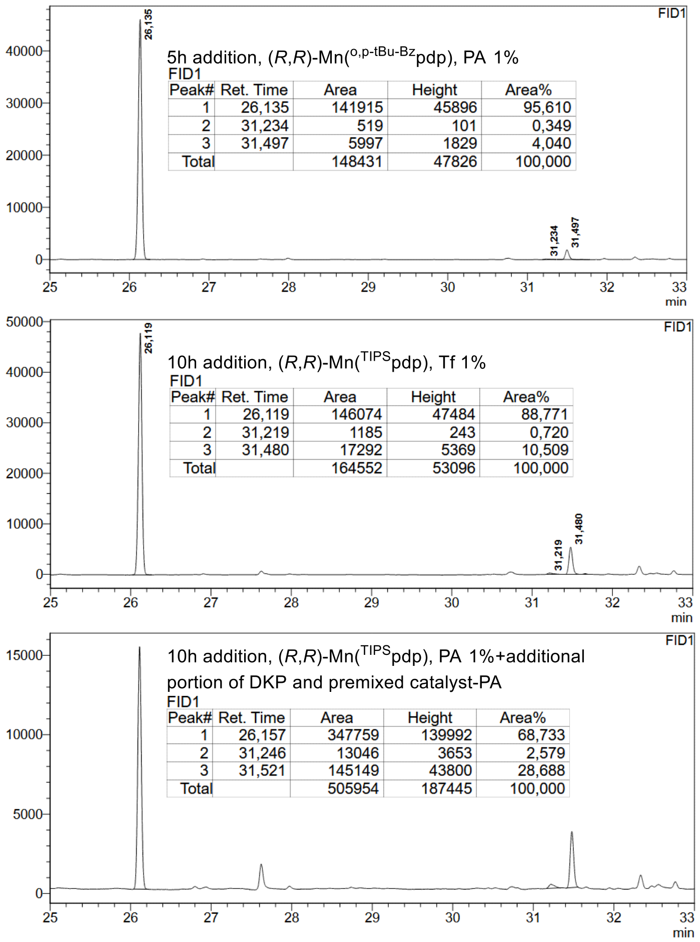

Scheme 11b. GC chromatographs of crude reactions for testing the effect of acidic additive in premixing conditions with the catalyst. For conditions used please advise table 3.

Results

Fortuitously, the premixing protocol affected reaction's profile, with the milder pivalic acid (pKa 5.01) being the most efficient additive among those tested (Table 3). Using over stoichiometric amounts of pivalic acid (3%) relative to the catalyst spectacularly enhanced the yield of the products to 11.2% (Table 3; entry 5) at 5h addition of Hantzsch ester. However, a significant increase in the amount of pivalic acid (10%) (Table 3; entry 6) led to a decrease in yields, suggesting an interference of pivalic acid with the DKP catalytic cycle. Finally, increasing the addition time to 10h with stoichiometric amount of pivalic acid relative to the catalyst resulted in the best yield, 20% (Table 3; entry 8). The bulkiness of the intermediate (catalyst-acid) formed also seems to play a pivotal role in the reaction's outcome. The more sterically hindered the complex of the catalyst with the acid, the lower the yield of the oxidation products (Table 3; entries 3,7 and 10). Lastly, an additional portion of DKP (10 mol%) and premixed catalyst with pivalic acid (1 mol%) after 10 hours of addition improved the yield to slightly over 30% (Table 3; entry 13).

5. Substrate preparation

Among the substrates used to explore the oxidation for inactivated CH-bonds, cyclic alkanes (**24** and **25**), benzylic compounds (**13**, **14**, **15** and **16**), as well as 4-methyl pentanoic acid and Ambroxide (**26**) were commercially available, while the rest of them were synthesized using known procedures. In this section, experimental procedures for the synthesized substrates are described.

5.1. Aliphatic chains

3,7-dimethyloctan-1-ol (**SI-2**)

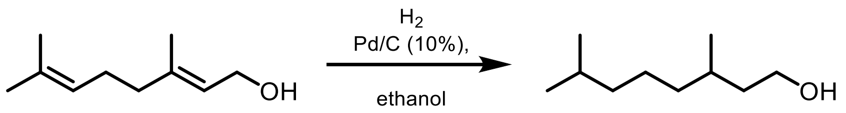

In a sealed tube, geraniol (1000 mg, 6.48 mmol, 1.0 equiv.) was dissolved in ethanol (8 mL) and palladium on activated carbon (56 mg, 10% mmol) was added, followed by bubbling with H_2 for 15 min. The tube was then sealed, and the reaction mixture was left stirring at room temperature for 4 hours. The resulting black suspension was filtered through celite, and the filtrate was evaporated to dryness. Purification by flash chromatography (SiO_2, eluent: hexane/EtOAc 10:1, TLC: R_f = 0.46 upon hexane/EtOAc 4:1, UV inactive on TLC, stains blue upon *anisaldehyde* staining) affords the product as a colourless oil (800 mg, 78%); Spectral data match those previously reported.[14] ^{1}H NMR (500 MHz, $CDCl_3$): δ_H 3.72 – 3.62 (m, 2H), 1.64 – 1.47 (m, 3H), 1.37 (dt, *J* = 13.1, 6.7 Hz, 1H), 1.31 – 1.22 (m, 3H), 1.17 – 1.08 (m, 3H), 0.88 (d, *J* = 6.6 Hz, 3H), 0.86 (d, *J* = 6.6 Hz, 6H) ppm; ^{13}C NMR (125 MHz, $CDCl_3$): δ_C 61.2, 39.9, 39.2, 37.3, 29.5, 28.0, 24.7, 22.7, 22.6, 19.6 ppm.

3,7-dimethyloctyl acetate (**3**)

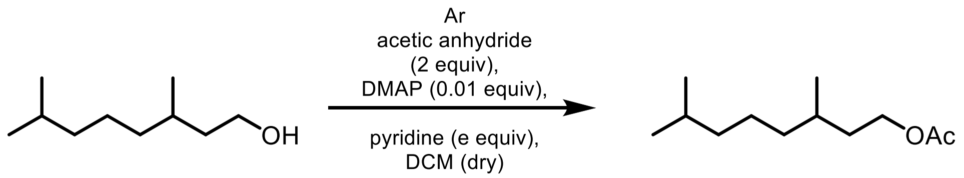

Under Ar atmosphere, **SI-2** (150 mg, 0.95 mmol, 1 equiv.) was dissolved in anhydrous CH_2Cl_2 (4.9 mL). Then, pyridine (153 µL, 1.89 mmol, 2 equiv.), acetic anhydride (180 µL, 1.89 mmol, 2 equiv.) and 4-dimethylaminopyridine (1.2 mg, 0.01 mmol, 0.01 equiv.) were added to the reaction mixture, which was subsequently left stirring, at room temperature, for 4 hours. The resulting solution was quenched with HCl (1N) and extracted. The aqueous layer was further washed with CH_2Cl_2 (3x5 mL). The combined organic layers were dried over $MgSO_4$, filtered and the solvent was removed under reduced pressure. Purification by flash chromatography (SiO_2, eluent: hexane, TLC: R_f = 0.75 upon hexane/EtOAc 20:1, UV inactive on TLC, stains light blue upon *anisaldehyde* staining) affords the product as a colourless oil (147 mg, 77% yield); Spectral data match those previously reported.[15] ^{1}H NMR (500 MHz, $CDCl_3$): δ_H 4.14 – 4.04 (m, 2H), 2.04 (s, 3H), 1.65 (td, *J* = 13.0, 7.2 Hz, 1H), 1.57 – 1.48 (m, 2H), 1.43 (td, *J* = 13.7, 7.3 Hz, 1H), 1.34 – 1.21 (m, 3H), 1.19 – 1.09 (m, 3H), 0.90 (d, *J* = 6.6 Hz, 3H), 0.87 (d, *J* = 6.6 Hz, 6H) ppm; ^{13}C NMR (125 MHz, $CDCl_3$): δ_C 171.3, 63.1, 39.2, 37.1, 35.5, 29.8, 27.9, 24.6, 22.7, 22.6, 21.1, 19.5 ppm.

3,7-dimethyloctyl pivalate (**10**)

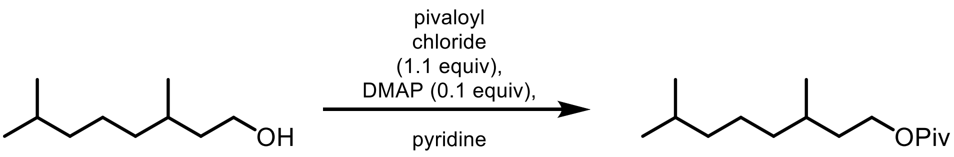

SI-2 **10**

3,7-dimethyl-1-octanol (**SI-2**) (350 mg, 2.17 mmol, 1 equiv.) and 4-dimethylaminopyridine (24 mg, 0.2 mmol, 0.1 equiv.) were dissolved in pyridine (3.5 mL). The mixture was cooled in an ice-bath and a solution of pivaloyl chloride (0.3 mL, 2.44 mmol, 1.1 equiv.) in pyridine (3 mL) was added dropwise. After stirring for 24 hours, the solvent was removed under reduced pressure and the resulting residue was treated with $CHCl_3$ (25 mL) and washed with water (1x10 mL), saturated $NaHCO_3$ aqueous solution (1x10 mL) and saturated NaCl aqueous solution (1x10 mL). The organic phase was dried over $MgSO_4$, filtered and the solvent was removed under reduced pressure. Purification by flash chromatography (SiO_2, eluent: hexane/EtOAc 80:1, TLC: R_f = 0.86 upon hexane/EtOAc 10:1, UV inactive on TLC, stains light blue upon *anisaldehyde* staining) affords the product as a colourless oil (526 mg, 72% yield); Spectral data match those previously reported.[16] ^{1}H NMR (500 MHz, $CDCl_3$): δ_H 4.15 – 4.04 (m, 2H), 1.65 (td, J = 13.1, 6.9 Hz, 1H), 1.51 (td, J = 13.1, 6.5 Hz, 2H), 1.41 (td, J = 13.8, 6.8 Hz, 1H), 1.33 – 1.22 (m, 3H), 1.18 (s, 9H), 1.16 – 1.09 (m, 3H), 0.89 (d, J = 6.6 Hz, 3H), 0.86 (d, J = 6.6 Hz, 6H) ppm; ^{13}C NMR (125 MHz, $CDCl_3$): δ_C 178.6, 62.9, 39.2, 38.7, 37.1, 35.5, 29.8, 27.9, 27.2, 24.6, 22.7, 22.6,19.6 ppm.

3,7-dimethyloctyl 2,2,2-trifluoroacetate (**11**)

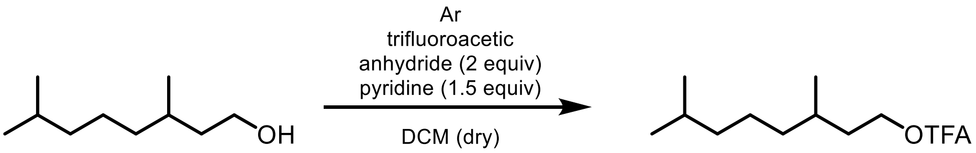

SI-2 **11**

Under Ar atmosphere, **SI-2** (150 mg, 0.95 mmol, 1 equiv.) was dissolved in anhydrous CH_2Cl_2 (1.5 mL). Then, pyridine (115 µL, 1.42 mmol, 1.5 equiv.) was added to the reaction mixture, which was subsequently cooled to 0°C using an ice bath. Dropwise addition of trifluoroacetic anhydride (265 µL, 1.89 mmol, 2 equiv.) followed, and the reaction mixture was left stirring, at room temperature, overnight. The next day, the resulting yellowish solution was concentrated under reduced pressure. Purification by flash chromatography (SiO_2, eluent: hexane, TLC: R_f = 0.85 upon hexane/EtOAc 20:1, UV inactive on TLC, stains light blue upon *anisaldehyde* staining) affords the product as a colourless oil (242 mg, 90% yield); Spectral data match those previously reported.[17] ^{1}H NMR (500 MHz, $CDCl_3$): δ_H 4.43 – 4.34 (m, 2H), 1.82 – 1.74 (m, 1H), 1.60 – 1.49 (m, 3H), 1.35 – 1.23 (m, 3H), 1.19 – 1.11 (m, 3H), 0.92 (d, J = 6.3 Hz, 3H), 0.87 (d, J = 6.6 Hz, 6H) ppm; ^{13}C NMR (125 MHz, $CDCl_3$): δ_C 157.5 (q, J = 42.1 Hz), 114.5 (q, J = 285.7 Hz), 66.8, 39.0, 36.9, 34.9, 29.6, 27.9, 24.5, 22.6, 22.5, 19.3 ppm.

<u>Notes</u>

- Column chromatography was performed as quickly as possible, as substrate **11** seems to decompose when it comes in contact with SiO_2. Neutralized SiO_2 (with Et_3N) for the isolation of **11** was also tested, without any improvement.

tert-butyl ((3,7-dimethyloctyl)oxy)dimethylsilane (**12**)

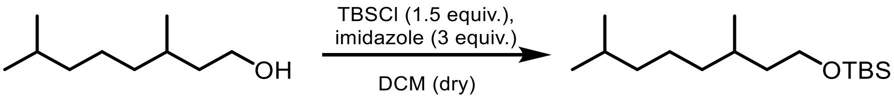

SI-2 **12**

Under Ar atmosphere, **SI-2** (50 mg, 0.31 mmol, 1 equiv.) was dissolved in anhydrous CH_2Cl_2 (15 mL). Then, TBSCl (88 µL, 0.47 mmol, 1.5 equiv.) was added to the reaction mixture, followed by the addition of imidazole (64.5 mg, 0.95 mmol, 3 equiv.) in three portions. After stirring for 1 hour, the was diluted in CH_2Cl_2 and extracted with H_2O (20 mL). The aqueous layer was further washed with CH_2Cl_2 (3 x 10 mL). The combined organic phases were dried over anhydrous $MgSO_4$, filtered and concentrated under reduced pressure. Purification by flash chromatography (SiO_2, hexane, TLC: R_f = 0.85 upon hexane/EtOAc 20:1, UV inactive on TLC, stains light blue upon *anisaldehyde* staining) affords the product as a colourless oil (67.5 mg, 80% yield); Spectral data match those previously reported.[18] ^{1}H NMR (500 MHz, $CDCl_3$): δ_H 3.69 – 3.58 (m, 2H), 1.58 – 1.48 (m, 3H), 1.35 – 1.20 (m, 4H), 1.16 – 1.07 (m, 3H), 0.89 (d, J = 2.6 Hz, 9H), 0.86 (d, J = 6.5 Hz, 9H), 0.04 (s, 6H) ppm; ^{13}C NMR (125 MHz, $CDCl_3$): δ_C 61.5, 40.0, 39.2, 37.3, 29.4, 27.9, 26.0, 25.6, 24.7, 22.7, 22.6, 19.7, 18.3, -5.3 ppm.

5.2. Carboxylic acids

3,7-dimethyloctanoic acid (**18**)

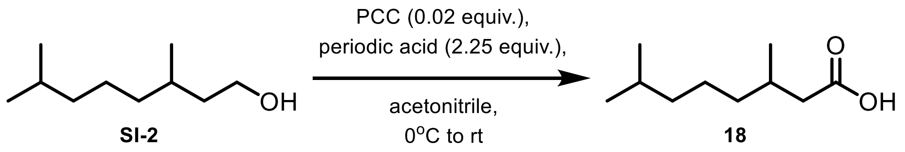

A round bottom flask containing a suspension of periodic acid (486 mg, 2.13 mmol, 2.25 equiv.) in acetonitrile (6.7 mL), was left stirring at room temperature for 40 minutes. Then, PCC (4.1 mg, 0.02 mmol, 0.02 equiv.) was added to the reaction mixture and the latter was cooled to 0°C using a water bath. At 0°C, **SI-2** (150 mg, 0.95 mmol, 1 equiv.) was added dropwise to the reaction vessel, diluted in acetonitrile (2 mL). After the completion of the addition, the mixture was left stirring at 0°C for 1hour, and at room temperature for 1hour and 40 minutes. Upon completion, the reaction mixture was concentrated under reduced pressure. The residue was then washed with EtOAc (3x10 mL). The combined organic phases were washed with H_2O and brine, dried over anhydrous $MgSO_4$, filtered and concentrated under reduced pressure, to afford the product as a colourless oil (88.3 mg, 54%), which was used with no further purification. Spectral data match those previously reported.[19] ^{1}H NMR (500 MHz, $CDCl_3$): δ_H 2.35 (dd, J = 15.0, 5.9 Hz, 1H), 2.14 (dd, J = 15.0, 8.2 Hz, 1H), 1.99 – 1.92 (m, 1H), 1.52 (dp, J = 13.3, 6.6 Hz, 1H), 1.36 – 1.27 (m, 3H), 1.21 – 1.13 (m, 3H), 0.97 (d, J = 6.7 Hz, 3H), 0.86 (d, J = 6.6 Hz, 6H) ppm; ^{13}C NMR (125 MHz, $CDCl_3$): δ_C 179.2, 41.5, 39.0, 36.9, 30.1, 27.9, 24.6, 22.6, 22.5, 19.7 ppm.

1-ethylcyclohexane-1-carboxylic acid (**19**)

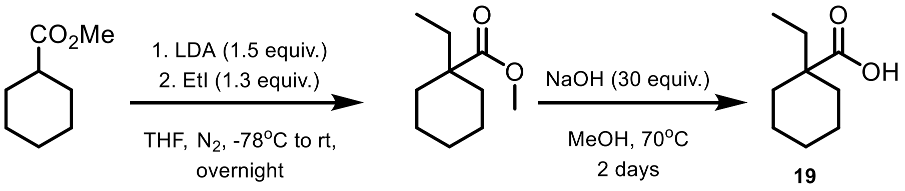

Under N_2 atmosphere, 3 mL of diisopropylamine (21.1 mmol, 1.5 equiv.) were dissolved in anhydrous THF (30 mL). The mixture was cooled to -78°C and n-BuLi (2.5 M in hexane, 8.4 mL, 21.1 mmol, 1.5 equiv.) were added dropwise. When the addition was completed, 1 equiv. of methyl cyclohexanecarboxylate (2 g, 2.01 mL, 14.1 mmol, 1 equiv.) was added and the mixture was stirred at -78°C for 1 h before 1.3 equiv. of ethyl iodide (1.5 mL, 18.24 mmol) were added at once. The reaction was stirred at room temperature overnight. Then, it was quenched with saturated NH_4Cl(aq) (10 mL), acidified with HCl 2 M (20 mL) and the product was extracted with Et_2O (3 x 15mL). The combined organic phases were dried over anhydrous $MgSO_4$, filtered and concentrated under reduced pressure. The resulting oil was dissolved in MeOH (30 mL), and solid NaOH (17 g, 30 equiv.) were added. The reaction mixture was heated at reflux for two days. Then, the mixture was cooled to room temperature, and the solvent was removed under reduced pressure. The resulting residue was diluted with H_2O (20 mL) and

washed with CH_2Cl_2 (3 x 10 mL). The aqueous layer was acidified to pH = 1 with 2 M HCl and the product was extracted with CH_2Cl_2 (3 x 15 mL). The combined organic phases were dried over anhydrous $MgSO_4$, filtered and concentrated under reduced pressure. The crude was purified by column chromatography (SiO_2, CH_2Cl_2) to afford the product as a white solid (1.49 g, 9.54 mmol, 68% yield). Spectral data match those previously reported.[9] ^{1}H-NMR (400 MHz, $CDCl_3$): δ_H 11.73 (brs, 1H), 2.10 – 2.06 (m, 2H), 1.64 – 1.57 (m, 5H), 1.47 – 1.38 (m, 2H), 1.29 – 1.20 (m, 3H), 0.89 (t, *J* = 7.6 Hz, 3H) ppm. ^{13}C-NMR (101 MHz, $CDCl_3$): δ_C 183.9, 47.4, 33.6, 33.2, 26.1, 23.4, 8.6 ppm.

5.3. Complex molecules

(3S,3aR,4aR,5R,7aR,9aR)-4a-hydroxy-3-(methoxymethyl)-5-methyl-8-methylenedecahydroazuleno[6,5-b]furan-2(3H)-one **(28)**

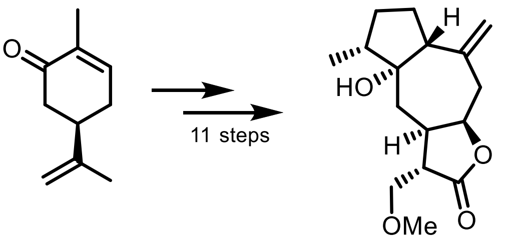

28

Compound **28** was obtained via a previously described 11 step procedure, starting from (*R*)-carvone. Purification by flash chromatography (SiO_2, eluent: benzene/Et_2O 10:1, TLC: R_f = 0.2 upon benzene/Et_2O 4:1, UV active on TLC, stains green upon *anisaldehyde* staining) affords the product as a white amorphous solid (in a 0.2 mmol scale, 72% yield, 40 mg); $[\alpha]^{20}_D$ = +34.3 (*c* 0.23, $CHCl_3$). Spectral data match those previously reported.[20] ^{1}H NMR (500 MHz, $CDCl_3$): δ_H 5.14 (s, 1H), 4.95 (s, 1H), 4.58 (ddd, *J* = 12.2, 8.7, 3.5 Hz, 1H), 3.66 (qd, *J* = 9.4, 4.2 Hz, 2H), 3.36 (s, 3H), 2.94 (dtd, *J* = 11.8, 9.1, 5.9 Hz, 1H), 2.74 (dd, *J* = 12.6, 3.4 Hz, 1H), 2.53 - 2.47 (m, 1H), 2.29 (dt, *J* = 11.7, 5.7 Hz, 3H), 1.96 -1.76 (m, 3H), 1.74 - 1.67 (m, 1H), 1.51 (dd, *J* = 14.4, 11.8 Hz, 2H), 1.36 (m, 1H), 0.96 (d, *J* = 6.7 Hz, 3H) ppm; ^{13}C NMR (125 MHz, $CDCl_3$): δ_C 176.3, 142.6, 115.1, 82.0, 79.5, 70.6, 59.2, 58.4, 47.3, 43.4, 40.7, 37.5, 36.2, 28.6, 24.0, 12.9 ppm.

(3S,3aR,4aR,5R,7aR,8R,9aR)-4a-hydroxy-3-(methoxymethyl)-5,8-dimethyldecahydroazuleno[6,5-b]furan-2(3H)-one (**27**)

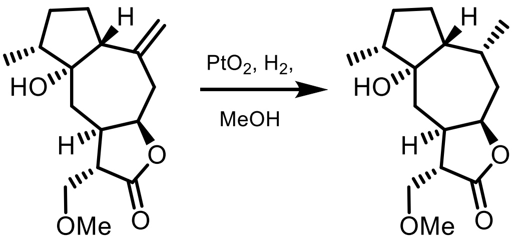

28 **27**

Compound (**xx**) (40 mg, 0.14 mmol, 1.0 equiv.) was dissolved in CH_3OH (4 mL) in a sealed tube, Adams catalyst (PtO_2*H_2O) (3 mg, 10% mmol) was added, followed by bubbling with H_2 for 15 min. The tube was then sealed, and the reaction mixture was left stirring at room temperature for 2 hours. The resulting black suspension is filtered through celite, and the filtrate was evaporated to dryness. Purification by flash chromatography (SiO_2, eluent: benzene/Et_2O 8:1, TLC: R_f = 0.24 upon benzene/Et_2O 4:1, UV inactive on TLC, stains green upon *anisaldehyde* staining) affords the product as a white amorphous solid (37 mg, 93%); $[\alpha]^{20}_D$ = +46.7 (*c* 0.3, $CHCl_3$). Spectral data match those previously reported.[20] ^{1}H NMR (500 MHz, $CDCl_3$): δ_H 5.02 (ddd, *J* = 11.9, 8.7, 2.8 Hz, 1H), 3.70 – 3.62 (m, 2H), 3.37 (s, 3H), 3.01 – 2.91 (m, 1H), 2.48 (dt, *J* = 8.3, 3.7 Hz, 1H), 2.21 –2.11 (m, 2H), 2.05 – 2.01(m, 1H), 1.94 – 1.85 (m, 2H), 1.78 – 1.67 (m, 2H), 1.60 – 1.54 (m, 2H), 1.40 (dd, *J* = 14.3, 11.8 Hz, 1H), 1.33 – 1.28 (m, 2H), 1.15

(d, *J* = 7.3Hz, 3H), 0.88 (d, *J* = 6.9 Hz, 3H) ppm; ^{13}C NMR (125 MHz, $CDCl_3$): δ_C 176.8, 82.7, 78.8, 70.6, 59.2, 55.2, 47.3, 43.7, 37.8, 37.1, 36.7, 29.9, 28.8, 24.9, 13.9, 12.4 ppm.

6. Reaction scope of the DKP-Mn co-catalysed aerobic CH-oxidation reaction

Using the optimized conditions as defined above (section 4 of SI; Table 3; entry 8), we sought to test the efficiency of the proposed oxidation method in different substrates. Along with the aerobic conditions, reactions with H_2O_2 peroxide were also carried out, for each substrate, to qualitatively compare the detected products. Below, experimental procedures are described for both methods. It is important to underline that in case of the H_2O_2 reactions, yields might differ if other combinations of solvents, manganese catalysts and carboxylic acids are applied, but further experimentation was out of this paper's scope.

As described thoroughly in the manuscript, in all cases, oxidation of both tertiary and secondary C(sp^3)–H bonds were detected, without any trace of activation of primary positions. Among the substrates tested the benzylic and cyclic ones seem to give the best results, followed by carboxylic and aliphatic substrates, while the more complex molecules provided diminished yields. In all cases, reactions carried under aerobic conditions seem more selective in terms of chemo selectivity, while overoxidation products (mainly ketones deriving from the corresponding alcohols) are absent in sharp contrast with the catalytic conditions with the aid of hydrogen peroxide. Further observations are given in each category of substrates separately.

6.1 General procedures for CH oxidation with optimized catalytic conditions

General oxidation protocol under aerobic conditions (Method A)

A 2ml vial was charged with manganese catalyst (0.001 equiv.) and a magnetic stirring bar. HFIP (0.1 ml) was added, along with the pivalic acid (0.001 equiv.) and the solution was left stirring, at room temperature, for 10 minutes (solution **S1**). A 10 ml vial was charged with the substrate (1 equiv.) and a magnetic stirring bar. The substrate was diluted to HFIP (2 mL) and subsequently HE (**2**) (2 equiv.), DKP (**1**) (0.1 equiv.) and **S1** were added. The vial was tightly capped by a rubber septum. An exit needle and a pipette were introduced in the septum, through which dioxygen was bubbled continuously by a balloon. Care was taken to adjust slow bubbling in the reaction vial to avoid solvent's evaporation. Also, a syringe charged with a solution of HE (**2**) in HFIP (8 equiv. in 6 mL) was adjusted to the septum, the other edge of which was introduced to a syringe pump (the addition was adjusted in such rate to achieve the 10 hours addition). The reaction mixture was stirred at room temperature, under dioxygen atmosphere, overnight.

General oxidation protocol with the aid of H_2O_2 (Method B)

A 4ml vial was charged with the substrate (1 equiv.) and a magnetic stirring bar. The substrate was diluted to acetonitrile (2 mL). Subsequently, acetic acid (1.5 equiv.) and the catalyst (0.03 equiv.) were added, and the reaction mixture was cooled at 0°C, using an ice bath. Slow addition of a solution of H_2O_2 30% in acetonitrile (3 equiv. in 2 mL) followed, via a syringe, over 2h. After syringe addition, the solution was stirred for 10 minutes at 0°C and at room temperature overnight.

<u>*Notes*</u>

When Method B was applied in carboxylic acids for lactonization, acetonitrile was replaced with HFIP, and the addition of acetic acid was no further needed (according to reported methods).[9]

For both methods

For GC analysis: 60-120 μL of the crude reaction mixture were filtered through a thin pad of celite, which was further washed with acetonitrile (up to a final volume of 1 mL), and selected to a GC vial.
For isolation of products: The reaction mixture was evaporated under reduced pressure and the residue was diluted with EtOAc (6 mL) and extracted with water (6 mL). The aqueous layer was further washed with EtOAc (3x6 mL). The combined organic layers were dried with Na_2SO_4, filtered and the solvent was removed under

reduced pressure. ^{1}HNMR analysis and column chromatography followed for qualitative analysis, while quantitative analysis was performed with GC.

6.2 Oxidized products

Aliphatic chains

Saturated geraniols, functionalized with various electron-donating and electron-withdrawing groups, were tested under aerobic conditions. The reactions consistently exhibited superior regioselectivity at the C-7 position, with substrate **11** (baring the most electron-withdrawing group) affording exclusively the remote oxidation product at C-7 (**SI-4**). No trace of overoxidized products was detected.

7-hydroxy-3,7-dimethyloctyl acetate (**4**)

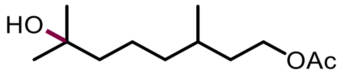

Isolated with (*R*,*R*)-Mn(^{TIPS}pdp) in a 0.1 mmol scale. Purification by flash chromatography (SiO_2, eluent: hexane/EtOAc 3:1, TLC: R_f = 0.29 upon hexane/EtOAc 3:1, UV inactive on TLC, stains blue upon *seebach* staining) affords the product as a colourless oil (Method A: 5.6 mg, 26% yield, Method B: 11.2 mg, 52% yield); Spectral data match those previously reported.[21] ^{1}H NMR (500 MHz, $CDCl_3$): δ_H 4.14 – 4.05 (m, 2H), 2.04 (s, 3H), 1.66 (td, *J* = 13.0, 7.2 Hz, 1H), 1.55 (td, *J* = 12.9, 6.9 Hz, 1H), 1.47 – 1.39 (m, 4H), 1.32 (ddd, *J* = 15.6, 9.5, 6.3 Hz, 3H), 1.21 (s, 6H), 0.91 (d, *J* = 6.6 Hz, 3H) ppm; ^{13}C NMR (125 MHz, $CDCl_3$): δ_C 171.3, 71.0, 63.0, 44.1, 37.3, 35.4, 29.8, 29.3, 29.2, 21.6, 21.1,19.4 ppm.

3-hydroxy-3,7-dimethyloctyl acetate (**5**)

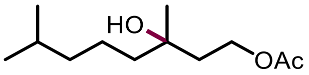

Isolated with (*R*,*R*)-Mn(^{TIPS}pdp) in a xx mmol scale. Purification by flash chromatography (SiO_2, eluent: hexane/EtOAc 4:1, TLC: R_f = 0.39 upon hexane/EtOAc 3:1, UV inactive on TLC, stains blue upon *seebach* staining) affords the product as a colourless oil (Method A: 1 mg, 5% yield, Method B: 1.7 mg, 8% yield); Spectral data match those previously reported.[21] ^{1}H NMR (500 MHz, $CDCl_3$): δ_H 4.22 (t, *J* = 7.0 Hz, 2H), 2.04 (s, 3H), 1.85 – 1.77 (m, 2H), 1.54 (dt, *J* = 13.3, 6.6 Hz, 1H), 1.47 – 1.42 (m, 2H), 1.36 – 1.29 (m, 2H), 1.20 (s, 3H), 1.17 (dd, *J* = 15.1, 7.1 Hz, 2H), 0.87 (d, *J* = 6.6 Hz, 6H) ppm; ^{13}C NMR (125 MHz, $CDCl_3$): δ_C 171.0, 71.9, 61.3, 42.7, 39.6, 39.4, 27.9, 27.0, 22.6, 21.6, 21.0 ppm.

7-hydroxy-3,7-dimethyloctyl pivalate (**SI-3**)

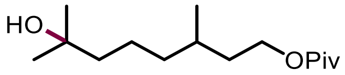

Isolated with (*R*,*R*)-Mn(^{TIPS}pdp) in a 0.1 mmol scale. Purification by flash chromatography (SiO_2, eluent: hexane/EtOAc 6:1, TLC: R_f = 0.27 upon hexane/EtOAc 10:1, UV inactive on TLC, stains purple upon *anisaldehyde* staining) affords the product as a colourless oil (Method A: 3.6 mg, 14% yield, Method B: 6.2 mg, 24% yield); Spectral data match those previously reported.[22] ^{1}H NMR (500 MHz, $CDCl_3$): δ_H 4.13 – 4.03 (m, 2H), 1.66 (dt, *J* = 12.4, 5.4 Hz, 1H), 1.55 (dd, *J* = 11.8, 5.7 Hz, 1H), 1.47 – 1.37 (m, 4H), 1.35 – 1.28 (m, 3H), 1.20 (s, *J* = 1.1 Hz, 6H), 1.18 (s, *J* = 1.0 Hz, 9H), 0.91 (dd, *J* = 6.4, 1.1 Hz, 3H) ppm; ^{13}C NMR (125 MHz, $CDCl_3$): δ_C 178.6, 71.0, 62.8, 44.1, 38.7, 37.4, 35.5, 29.9, 29.3, 29.2, 27.2, 21.6, 19.5 ppm.

7-hydroxy-3,7-dimethyloctyl 2,2,2-trifluoroacetate (**SI-4**)

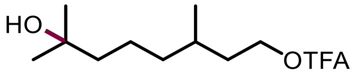

Isolated with (*R*,*R*)-Mn(^{TIPS}pdp) in a 0.15 mmol scale. Purification by flash chromatography (SiO_2, eluent: hexane/EtOAc 3:1, TLC: R_f = 0.68 upon hexane/EtOAc 2:1, UV inactive on TLC, stains blue-black upon *seebach* staining) affords the product as a colourless oil (Method A: 9.3 mg, 23% yield, Method B: 21 mg, 52% yield); HRMS (ESI-MS) m/z calculated for $C_{12}H_{22}F_3O_3^+$ ($[M+H]^+$): 271.1516, found 271.1515.^{1}H NMR (500 MHz, $CDCl_3$): δ_H 4.43 – 4.34 (m, 2H), 1.83 – 1.75 (m, 3H), 1.60 – 1.56 (m, 2H), 1.54 (s, 6H), 1.42 – 1.29 (m, 3H), 1.23 – 1.16 (m, 1H), 0.93 (d, *J* = 6.4 Hz, 3H) ppm; ^{13}C NMR (125 MHz, $CDCl_3$): δ_C 156.8 (m, 1C), 114.4 (m, 1C), 89.0, 66.6, 40.4, 36.6, 34.8, 29.4, 25.5, 20.8, 19.1 ppm.

Notes

- Column chromatography was performed as quickly as possible, as substrate **SI-4** (like its starting material **11**) seems to decompose when it comes in contact with SiO_2.

8-((tert-butyldimethylsilyl)oxy)-2,6-dimethyloctan-2-ol (**SI-5**)

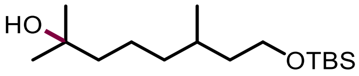

Isolated with (*R*,*R*)-Mn(^{TIPS}pdp) in a 0.1 mmol scale. Purification by flash chromatography (SiO_2, eluent: hexane/EtOAc 3:1, TLC: R_f = 0.5 upon hexane/EtOAc 1:1, UV inactive on TLC, stains blue upon *seebach* staining) affords the product as a colourless oil (Method A: 9.8 mg, 34% yield, Method B: 17.3 mg, 60% yield); Spectral data match those previously reported.[23] ^{1}H NMR (500 MHz, $CDCl_3$): δ_H 3.70 – 3.58 (m, 2H), 1.56 (dq, *J* = 12.1, 5.9 Hz, 2H), 1.46 – 1.27 (m, 7H), 1.21 (s, 6H), 0.93 – 0.86 (m, 12H), 0.05 (s, 6H) ppm; ^{13}C NMR (125 MHz, $CDCl_3$): δ_C 71.0, 61.4, 44.2, 39.9, 37.6, 29.5, 29.2, 26.0, 21.7, 19.7, 18.3, -5.3 ppm.

Lactones

To broaden the scope of the catalytic method, lactonization of carboxylic acids was also tested. In this case, directed (from the carboxylic acid moiety) oxidation of nonactivated tertiary and secondary C(*sp*^{3})–H bonds was observed. Notably, substrate **17**, in addition to the expected butenolide product (**20**), yielded compound **21** under both aerobic and hydrogen peroxide conditions. This product likely results from an epoxidation-lactonization sequence on a terminal alkene formed via a desaturation pathway (for details please advise the manuscript). Interestingly, the desaturation pathway was only evident in directed intramolecular hydroxyl rebound, as reported earlier. In the case of compound **18**, where tertiary lactonization is highly strained, non-selective aerobic lactonization at five- and six-membered secondary positions (compounds **22a,b**) was favoured. Moreover, both oxidation conditions produced high yields of unidentified alkene products, attributed to the unselective activation of the desaturase pathway.

5,5-dimethyldihydrofuran-2(3H)-one (**20**)

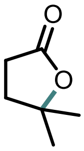

Isolated with (*R*,*R*)-Mn(^{TIPS}pdp) in a 0.15 mmol scale. Purification by flash chromatography (SiO_2, eluent: benzene/EtOAc 6:1, TLC: R_f = 0.64 upon benzene/EtOAc 1:1, UV inactive on TLC, stains light blue upon *anisaldehyde* staining) affords the product as a colourless oil (Method A: 3.9 mg, 23% yield, Method B: 11.6 mg, 68% yield);

Spectral data match those previously reported.[24] ^{1}H NMR (500 MHz, $CDCl_3$): δ_H 2.59 (t, *J* = 7.9 Hz, 2H), 2.03 (t, *J* = 8.2 Hz, 2H), 1.40 (s, 3H) ppm; ^{13}C NMR (125 MHz, $CDCl_3$): δ_C 176.7, 84.6, 34.6, 29.3, 27.7 ppm.

Notes

- Removal of the organic solvents under reduced pressure was performed at 0°C (using an ice bath), since product **20** seems to be volatile.

- The oxidation reaction of substrate **17** was also carried out in a sealed tube (for details please see Notes of substrate **SI-12**).

5-(hydroxymethyl)-5-methyldihydrofuran-2(3H)-one (**21**)

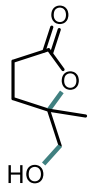

Isolated with (*R*,*R*)-Mn(^{TIPS}pdp) in a 0.15 mmol scale. Purification by flash chromatography (SiO_2, eluent: benzene/EtOAc 1:1, TLC: R_f = 0.25 upon benzene/EtOAc 1:1, UV inactive on TLC, stains purple upon *anisaldehyde* staining) affords the product as a colourless oil (Method A: 2.9 mg, 15% yield, Method B: 5.8 mg, 30% yield); Spectral data match those previously reported.[25] ^{1}H NMR (500 MHz, $CDCl_3$): δ_H 3.69 (d, *J* = 12.2 Hz, 1H), 3.50 (d, *J* = 12.1 Hz, 1H), 2.75 – 2.65 (m, 1H), 2.63 – 2.54 (m, 1H), 2.35 (ddd, *J* = 12.8, 10.5, 6.3 Hz, 1H), 1.91 (ddd, *J* = 12.8, 10.4, 7.0 Hz, 1H), 1.35 (s, 3H) ppm; ^{13}C NMR (125 MHz, $CDCl_3$): δ_C 177.7, 86.9, 68.3, 29.7, 29.6, 23.0 ppm.

5-isobutyl-4-methyldihydrofuran-2(3H)-one (**22a**) and 6-isopropyl-4-methyltetrahydro-2H-pyran-2-one (**22b**)

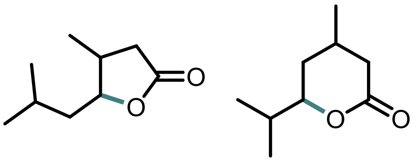

Isolated with (*R*,*R*)-Mn(^{TIPS}pdp) in a 0.1 mmol scale. Purification by flash chromatography (SiO_2, eluent: hexane/EtOAc 6:1, TLC: R_f = 0.52 upon hexane/EtOAc 1:1, UV inactive on TLC, stains brown upon *anisaldehyde* staining) affords the inseparable mixture of products as a colourless oil (Method A: 4 mg, 13% yield, Method B: 2.8 mg, 9% yield); Spectral data match those previously reported.[26]

5-(2-hydroxy-2-methylpropyl)-4-methyldihydrofuran-2(3H)-one (**23**)

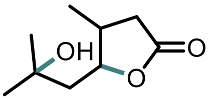

Isolated with (*R*,*R*)-Mn(^{TIPS}pdp) in a 0.1 mmol scale. Purification by flash chromatography (SiO_2, eluent: hexane/EtOAc 2:1, TLC: R_f = 0.4 upon hexane/EtOAc 4:1, UV inactive on TLC, stains brown upon *anisaldehyde* staining) affords the product as a colourless oil (Method A: -, Method B: 9.9 mg, 58% yield); HRMS (ESI-MS) m/z calculated for $C_9H_{16}NaO_3^+$ ($[M+Na]^+$): 195.0992, found 195.0993. ^{1}H NMR (500 MHz, $CDCl_3$): δ_H 4.01 (td, *J* = 8.0, 3.5 Hz, 1H), 2.68 (dd, *J* = 15.9, 6.7 Hz, 1H), 2.27 – 2.19 (m, 1H), 1.88 – 1.81 (m, 1H), 1.77 – 1.70 (m, 1H), 1.66 (ddd, *J* = 12.8, 7.7, 3.9 Hz, 1H), 1.57 – 1.50 (m, 1H), 1.24 (d, *J* = 7.1 Hz, 6H), 1.15 (d, *J* = 6.4 Hz, 3H) ppm; ^{13}C NMR (125 MHz, $CDCl_3$): δ_C 176.4, 87.7, 70.5, 39.5, 37.1, 36.2, 29.0, 28.7, 17.4 ppm.

1-ethyl-6-oxabicyclo[3.2.1]octan-7-one (**SI-6**)

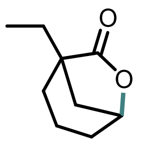

Isolated with (*S*,*S*)-Mn($^{iPr-Bz}$pdp) in a 0.15 mmol scale. Purification by flash chromatography (SiO_2, eluent: hexane/EtOAc 9:1, TLC: R_f = 0.72 upon hexane/EtOAc 2:1, UV inactive on TLC, stains yellowish upon *phosphomolybdic acid* staining) affords the product as a yellow oil (Method A: 4.6 mg, 20% yield, 77% ee, Method B: 11.6 mg, 50% yield, 97% ee); $[\alpha]_D^{20}$ = +18.13 (*c* 0.396, $CHCl_3$); Spectral data match those previously reported.[9] ^{1}H NMR (500 MHz, $CDCl_3$): δ_H 4.77-4.74 (m, 1H), 2.30-2.26 (m, 1H), 2.00-1.96 (m, 1H), 1.81-1.70 (m, 2H), 1.67-1.45 (m, 6H), 0.88 (t, *J* = 7.5 Hz, 3H) ppm; ^{13}C NMR (125 MHz, $CDCl_3$): δ_C 180.3, 75.8, 46.8, 40.3, 32.5, 27.9, 27.1, 18.8, 8.7 ppm.

<u>*Notes*</u>

- The enantioselectivity of this product was measured by chiral GC: Cyclosyl-B column was used. GC temperature program: starting at 75°C for 0.5 minutes, then raised to 120°C at 10°C/min and hold 60 minutes. Then raised to 220°C at 5°C/min and hold 2 minutes. The analysis time was 87 minutes. t_1= 22.1 min, t_2= 22.9 min.

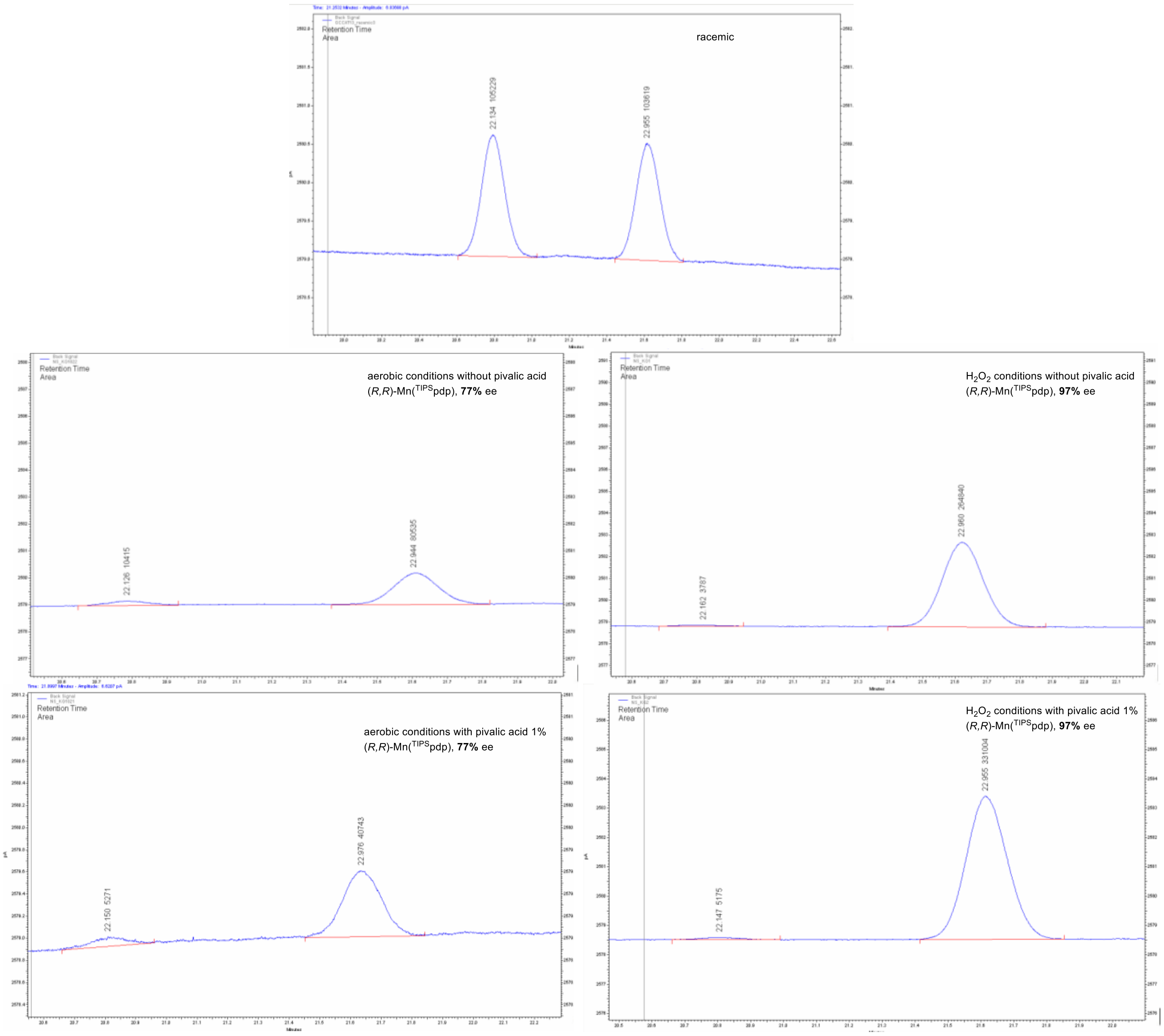

Scheme 12. Chiral GC analysis to measure ee% for product **SI-6**. Conditions used: *aerobic conditions* refer to substrate (0.05 mmoles), catalyst (1 mol%), DKP (10 mol%), HE (10 equiv.), PA (1%) or none as indicated, premixing of acid and catalyst, 10h addition, HFIP, rt stirring overnight under dioxygen atmosphere; *H_2O_2 conditions* refer to substrate (0.05 mmoles), catalyst (1 mol%), H_2O_2 (0.5 equiv.), PA (1%) or not as indicated, HFIP, 0°C to r.t. for 1h.

Complex molecules

Complex substrates bearing electron-withdrawing groups or heteroatoms showed diminished yields compared to the hydrogen peroxide variant or yielded only trace amounts of products. The pronounced effect of these substituents on product yields is attributed to their hydrogen-bonding deactivation in HFIP solvation. This effect is further highlighted by the complete absence of overoxidation products in aerobic reactions. A characteristic example of this pattern is the differentiation of products formed in the oxidation of substrate **26**. Under aerobic conditions the only oxidation product observed is lactol **SI-9**, while in the presence of hydrogen peroxide only sclareolide is isolated. Despite the reduced reactivity of C-H bonds under aerobic conditions, complex substrate **28** successfully reacted at the terminal alkene position to form the two epoxides. This stands in sharp contrast to previously reported aerobic oxidations relying solely on DKP, which failed to initiate the reaction. The preference in β-isomer in this case is dictated by the conformation of the molecule.

(4aR,5R,7aR,8S,9aR)-4a,8-dihydroxy-3-(methoxymethyl)-5,8-dimethyldecahydroazuleno[6,5-b]furan-2(3H)-one (**SI-7**)

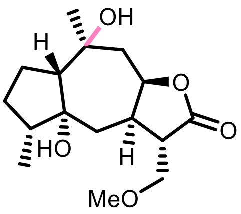

Isolated with (*R*,*R*)-Mn(^{TIPS}pdp) in a 0.1 mmol scale. Purification by flash chromatography (SiO_2, eluent: benzene/Et_2O 1:3, TLC: R_f = 0.15 upon benzene/Et_2O 1:1, UV inactive on TLC, stains green upon *anisaldehyde* staining) affords the product as a colourless oil (Method A: 3.6 mg, 12% yield, Method B: 13.1 mg, 44% yield); $[\alpha]^{20}_D$ = -36.6 (*c* 0.8, $CHCl_3$). Spectral data match those previously reported.[20] ^{1}H NMR (500 MHz, $CDCl_3$): δ_H 4.82 – 4.77 (m, 1H), 3.65 (t, *J* = 4.2 Hz, 2H), 3.36 (s, 3H), 2.95 – 2.86 (m, 1H), 2.51 – 2.46 (m, 1H), 2.22 (dd, *J* = 14.4, 6.3 Hz, 1H), 2.10 (t, *J* = 12.8 Hz, 1H), 2.01 (dd, *J* = 13.1, 2.6 Hz, 2H), 1.93 – 1.85 (m, 2H), 1.83 – 1.73 (m, 5H), 1.41 (s, 3H), 0.89 (d, *J* = 6.8 Hz, 3H) ppm; ^{13}C NMR (125 MHz, $CDCl_3$): δ_C 176.3, 79.6, 78.2, 72.5, 70.2, 61.8, 59.3, 46.8, 45.9, 43.6, 37.4, 35.9, 28.1, 23.4, 22.3, 12.4 ppm.

(4aR,5R,7aS,8R,9aR)-4a-hydroxy-3-(methoxymethyl)-5-methyldecahydro-2H-spiro[azuleno[6,5-b]furan-8,2'-oxiran]-2-one (**SI-8β**)

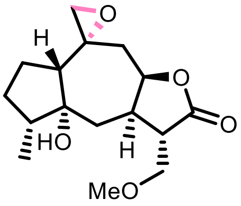

Isolated with (*R*,*R*)-Mn(^{TIPS}pdp) in a 0.1 mmol scale. Purification by flash chromatography (SiO_2, eluent: benzene/Et_2O 1:2, TLC: R_f = 0.34 upon benzene/Et_2O 1:1, UV inactive on TLC, stains green upon *anisaldehyde* staining) affords the product as a white amorphous solid (Method A: 6.5 mg, 22% yield, Method B: 16.3 mg, 55% yield); $[\alpha]^{20}_D$ = +31.6 (*c* 0.4, $CHCl_3$); Spectral data match those previously reported.[20] ^{1}H NMR (500 MHz, $CDCl_3$): δ_H 5.09 – 5.03 (m, 1H), 3.68 (qd, *J* = 9.5, 4.6 Hz, 2H), 3.37 (s, 3H), 3.13 – 3.04 (m, 1H), 2.56 (d, *J* = 4.7 Hz, 1H), 2.52 (d, *J* = 4.7 Hz, 1H), 2.48 (dd, *J* = 14.5, 6.7 Hz, 1H), 2.29 – 2.23 (m, 1H), 2.18 – 2.13 (m, 1H), 1.87 – 1.80 (m, 1H), 1.79 – 1.74 (m, 1H), 1.71 (dd, *J* = 13.7, 2.7 Hz, 2H), 1.49 – 1.29 (m, 5H), 0.94 (d, J = 6.6 Hz, 3H); ^{13}C NMR (125 MHz, $CDCl_3$): δ_C 176.3, 81.2, 78.7, 70.5, 59.25, 55.6, 54.2, 51.1, 46.7, 44.0, 38.0, 36.9, 29.7, 28.2, 20.6, 12.3 ppm.

(4aR,5R,7aS,8S,9aR)-4a-hydroxy-3-(methoxymethyl)-5-methyldecahydro-2H-spiro[azuleno[6,5-b]furan-8,2'-oxiran]-2-one (**SI-8α**)

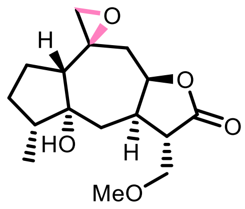

Isolated with (*R*,*R*)-Mn(^{TIPS}pdp) in a 0.1 mmol scale. Purification by flash chromatography (SiO_2, eluent: benzene/Et_2O 1:2, TLC: R_f = 0.22 upon benzene/Et_2O 1:1, UV inactive on TLC, stains green upon *anisaldehyde* staining) affords the product as a white amorphous solid (Method A: 2.4 mg, 8% yield, Method B: 8.3 mg, 28% yield); $[\alpha]^{20}_D$ = +42.5 (*c* 0.4, $CHCl_3$); HRMS (ESI, m/z) calculated for $C_{16}H_{25}O_5^+$ ($[M+H]^+$): 297.1797, found: 297.1799. ^1H NMR (500 MHz, $CDCl_3$): δ_H 4.88 – 4.82 (m, 1H), 3.67 (dd, *J* = 4.2, 2.5 Hz, 2H), 3.37 (s, 3H), 3.00 – 2.98 (m, 1H), 2.72 (d, *J* = 4.0 Hz, 1H), 2.56 – 2.50 (m, 1H), 2.39 – 2.33 (m, 2H), 1.86 (t, *J* = 10.0 Hz, 2H), 1.65 – 1.56 (m, 5H), 1.49 (dd, *J* = 14.5, 11.6 Hz, 2H), 0.92 (dd, *J* = 12.8, 5.0 Hz, 3H) ppm; ^{13}C NMR (125 MHz, $CDCl_3$): δ_C 176.0, 80.1, 79.1, 70.0, 59.3, 57.0, 55.7, 48.7, 46.5, 43.6, 40.6, 37.2, 36.4, 28.4, 21.9, 12.3 ppm.

(3aR,5aS,9aS,9bR)-3a,6,6,9a-tetramethyldodecahydronaphtho[2,1-b]furan-2-ol (**SI-9**)

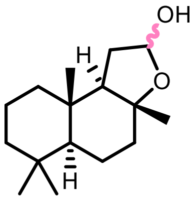

Isolated with (*R*,*R*)-Mn(^{TIPS}pdp) in a 0.15 mmol scale. Purification by flash chromatography (SiO_2, eluent: hexane/EtOAc 6:1, TLC: R_f = 0.3 upon hexane/EtOAc 5:1, UV inactive on TLC, stains blue upon *anisaldehyde* staining) affords the product as a white solid (Method A: 1.9 mg, 5% yield, Method B: -); Spectral data match those previously reported.[27] ^1H NMR (300 MHz, $CDCl_3$): δ_H 5.53 – 5.45 (m, 1H), 2.03 – 1.89 (m, 2H), 1.78 – 1.62 (m, 3H), 1.54 – 1.32 (m, 4H), 1.30 (s, 3H), 1.28 – 1.23 (m, 1H), 1.22 – 1.07 (m, H), 1.06 – 0.90 (m, 2H), 0.88 (s, 3H), 0.83 (s, 6H). ppm; ^{13}C NMR (75 MHz, $CDCl_3$): δ_C 101.8, 100.4, 82.6, 81.2, 60.1, 57.0 (2C), 56.9, 42.3, 40.2, 39.9, 39.8, 36.0, 33.5, 33.1 (2C), 30.8, 23.5, 21.1, 20.8, 20.5, 18.3, 15.3 ppm.

Cyclic alkanes

Two cyclic alkanes were tested under synergistic catalytic conditions **24** and **25**. They both showed good yields, that can be attributed to their rigid backbone, lowering the bond-dissociation energy of the oxidized position. Particularly, in the case of **24** stereospecificity was also observed, with a diastereomeric ratio exceeding 14:1. This substrate was a key in understanding the importance of solubility in achieving high selectivity. More specifically, under the optimized conditions cis-decalin provided an unexpected 82% yield, though with low diastereocontrol (cis:trans alcohol = 4:1). This was attributed to its complete insolubility in HFIP, leading to uncontrolled autooxidation processes in the dioxygen stream. When DCM was used as a co-solvent (HFIP: DCM = 3:1), the yield for cis-decalol reached 72%, with a diastereomeric ratio exceeding 14:1.

(4ar,8ar)-octahydronaphthalen-4a(2H)-ol (**SI-10**)

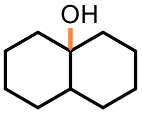

Isolated with (*R*,*R*)-Mn(^{TIPS}pdp) in a 0.1 mmol scale. Purification by flash chromatography (SiO_2, eluent: hexane/acetone 9:1, TLC: R_f = 0.68 upon hexane/acetone 3:1, UV inactive on TLC, stains blueish upon anisaldehyde staining) affords the product as a colourless solid (Method A: 11.1 mg, 72% yield, Method B: 4.2 mg, 27% yield). Spectral data match those previously reported.[15] ^{1}H NMR (500 MHz, $CDCl_3$): δ_H 1.80-1.24 (m, 17H) ppm; ^{13}C NMR (125 MHz, $CDCl_3$): δ_C 71.8, 42.8, 28.0, 22.7 ppm.

adamantan-1-ol (**SI-11**)

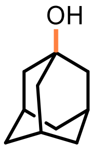

Isolated with (*R*,*R*)-Mn(^{TIPS}pdp) in a xx mmol scale. Purification by flash chromatography (SiO_2, eluent: hexane/EtOAc 2:1, TLC: R_f = 0.25 upon hexane/EtOAc 4:1, UV inactive on TLC, stains light blue upon *seebach* staining) affords the product as a colourless oil (Method A: 12.9 mg, 85% yield, Method B: 3.8 mg, 25% yield). Spectral data match those previously reported.[28] ^{1}H NMR (500 MHz, $CDCl_3$): δ_H 2.14 (s, 3H), 1.71 (d, *J* = 2.6 Hz, 6H), 1.61 (q, *J* = 12.3 Hz, 7H) ppm; ^{13}C NMR (125 MHz, $CDCl_3$): δ_C 68.2, 45.3, 36.1, 30.7 ppm.

Benzylic

Lastly, benzylic substrates were also tested under optimized catalytic conditions. The yields of the detected products were much higher compared to the aliphatic substrates previously tested. This was attributed to the partially activated benzylic position, which also explains the uncontrol oxidation observed in case of H_2O_2 reactions (overoxidized products were obtained to a great extent, mostly the ketones of the corresponding alcohols). The profile of the synergetic catalytic reaction seems much cleaner than the one observed in manganese catalysis with the aid of hydrogen peroxide. That implies two things: benzylic oxidations can take place under this method (while they do not occur at all under sole DKP catalysis) and they are selective (which does not apply in case of H_2O_2 reactions).

That gave us the opportunity to further explore the limits of the method, now in terms of enantioselectivity too. As described below, some enantioselectivity was detected in each benzylic substrate tested, providing a solid proof of the metal-oxo intermediate formed. Different manganese catalysts were tested in each case to unveil the full potential of the proposed method. Among those tested, (*R*,*R*)-Mn($^{CF3-Bz}$bpeb) seems to give the best results in terms of enantioselectivity. To measure and compare ee%, Method B was slightly changed to simulate as much as possible conditions of Method A (for details, please see the corresponding schemes 13-15). In these reactions, since measuring the yields was not the goal, only a small amount of H_2O_2 was used, in order to obtain a clean reaction profile (avoid overoxidations).

1,2,3,4-tetrahydronaphthalen-1-ol (**SI-12**)

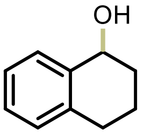

Isolated with (*R*,*R*)-Mn(TIPSpdp) in a 0.15 mmol scale. Purification by flash chromatography (SiO_2, eluent: hexane/EtOAc 4:1, TLC: R_f = 0.32 upon hexane/EtOAc 4:1, UV active on TLC, stains light blue upon *seebach* staining) affords the product as a colourless oil (Method A: 16.7 mg, 75% yield, 17% ee, Method B: 4 mg, 18% yield, racemic). Spectral data match those previously reported.[29] ^{1}H NMR (500 MHz, $CDCl_3$): δ_H 7.45 – 7.42 (m, 1H), 7.22 – 7.19 (m, 2H), 7.13 – 7.10 (m, 1H), 4.79 (t, *J* = 4.7 Hz, 1H), 2.87 – 2.80 (m, 2H), 2.77 – 2.70 (m, 2H), 2.02 – 1.89 (m, 4H), 1.82 – 1.76 (m, 1H) ppm; ^{13}C NMR (125 MHz, $CDCl_3$): δ_C 138.8, 137.1, 129.0, 128.6, 127.6, 126.2, 68.2, 32.3, 29.2, 18.8 ppm.

<u>*Notes*</u>

- During initial trials for the aerobic C-H oxidation of substrate **16**, an imbalance between substrate's conversion and mass balance was detected during GC analysis. This was attributed to the volatility of the starting material **16**, under bubbling conditions. To test our hypothesis the reaction was repeated in a sealed tube. Once the components were weighted and diluted in the solvent (according to the general oxidation protocol under aerobic conditions, Method A), the tube was capped, evacuated 3 times and then refilled with oxygen (using a balloon, which was then removed) and addition of Hantzsch ester followed, using a syringe pump, as described above. GC analysis revealed higher mass balance and yield.

- Substrate **SI-12** was successfully scaled up to 0.5 mmoles without any impact on its yield**,** using Method A. To isolate the product in this case, an extra work up was carried out, to get rid of the excess of Hantzsch pyridine formed during the reaction. More specifically, once the reaction was completed, the solvent was removed under reduced pressure and the residue was diluted to THF (20 mL). Then, 2N NaOH (20 mL) was added, and biphasic solution was left vigorously stirring at room temperature overnight. Extraction with EtOAc (20 mL) followed. The aquas layer was further washed with EtOAc (3x 10 mL). The combined organic layers were dried over anhydrous $MgSO_4$, filtered and concentrated under reduced pressure. Purification by flash chromatography afforded pure **SI-12**.

- For the SFC analysis: Chiralpak IB, CO_2: EtOH 95:5, 35 ºC, flow rate = 1.2 mL/min, λ = 210 nm. The analysis time was 4 minutes. t1 = 1.78 min, t2 = 1.95 min.

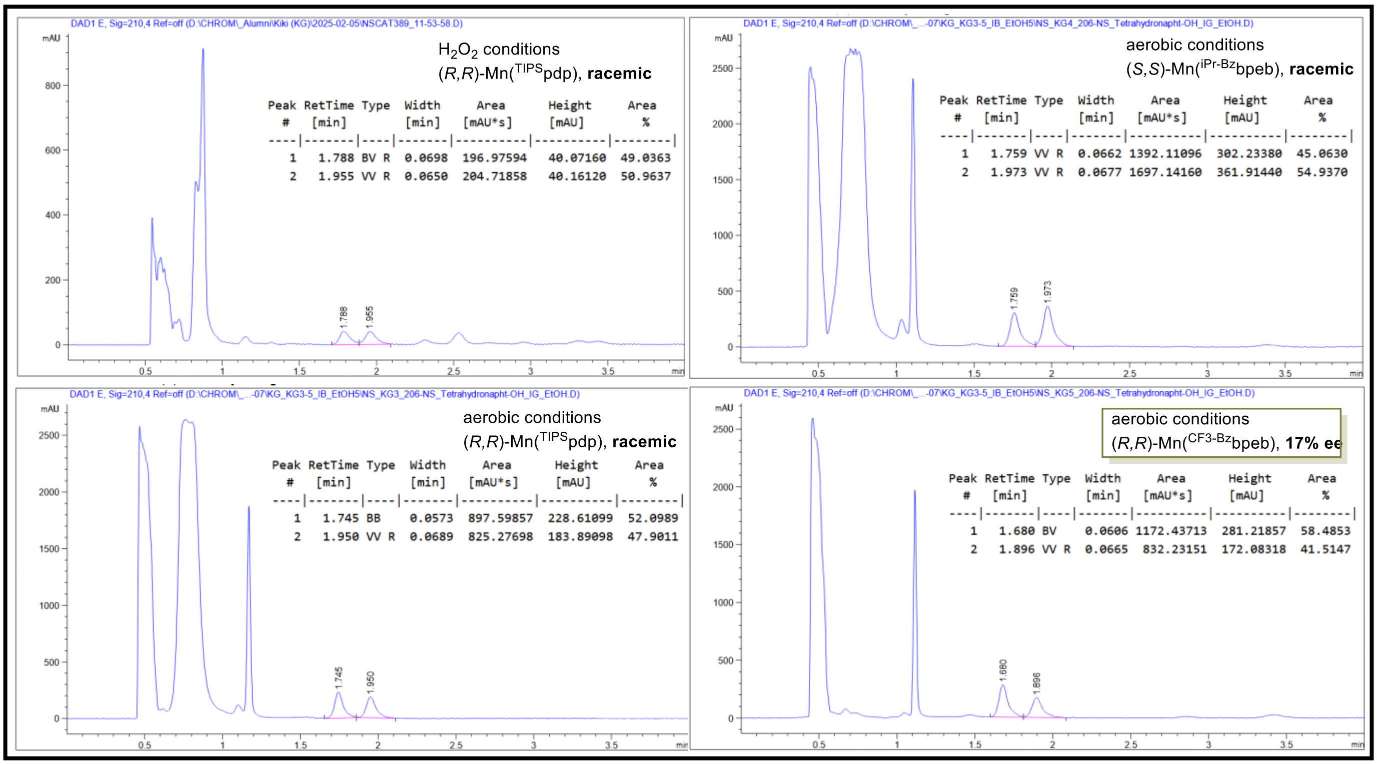

Scheme 13. SFC analysis to measure ee% for product **SI-12**. The best results are highlighted with a green frame. Conditions used: *aerobic conditions* refer to substrate (0.05 mmoles), catalyst (1 mol%), DKP (10 mol%), HE (10 equiv.), PA (1%), premixing of acid and catalyst, 10h addition, HFIP, rt stirring overnight under dioxygen atmosphere; *H_2O_2 conditions* refer to substrate (0.05 mmoles), catalyst (1 mol%), H_2O_2 (0.5 equiv.), PA (1%), HFIP, 0°C to r.t. for 1h.

2,3-dihydro-1H-inden-1-ol (**SI-13**)

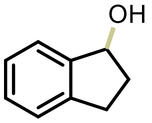

Isolated with (*R*,*R*)-Mn(^{TIPS}pdp) in a 0.15 mmol scale. Purification by flash chromatography (SiO_2, eluent: hexane/EtOAc 6:1, TLC: R_f = 0.43 upon hexane/EtOAc 4:1, UV active on TLC, stains pink upon *anisaldehyde* staining) affords the product as a white solid (Method A: 17.3 mg, 86% yield, 33% ee, Method B: 3.6 mg, 18% yield, 17% ee). Spectral data match those previously reported.[30] ^{1}H NMR (500 MHz, $CDCl_3$): δ_H 7.42 (d, *J* = 6.5 Hz, 1H), 7.28 – 7.23 (m, 3H), 5.24 (t, *J* = 6.0 Hz, 1H), 3.06 (ddd, *J* = 15.6, 8.5, 4.8 Hz, 1H), 2.86 – 2.79 (m, 1H), 2.49 (ddd, J = 13.1, 7.6, 5.0 Hz, 1H),1.99 – 1.91 (m, 2H) ppm; ^{13}C NMR (125 MHz, $CDCl_3$): δ_C 145.0, 143.3, 128.3, 126.7, 124.9, 124.2, 35.9, 29.8 ppm.

<u>*Notes*</u>

- Substrate **SI-13** was successfully scaled up to 0.5 mmoles without any impact on its yield**,** using Method A (for more details, please see notes of substrate **SI-12**).

- For the SFC analysis: Chiralpak IG, CO_2: MeOH 99:1, 35 ºC, flow rate = 1.5 mL/min, λ = 210 nm. The analysis time was 10 minutes. t1 = 6.32 min, t2 = 7.16 min.

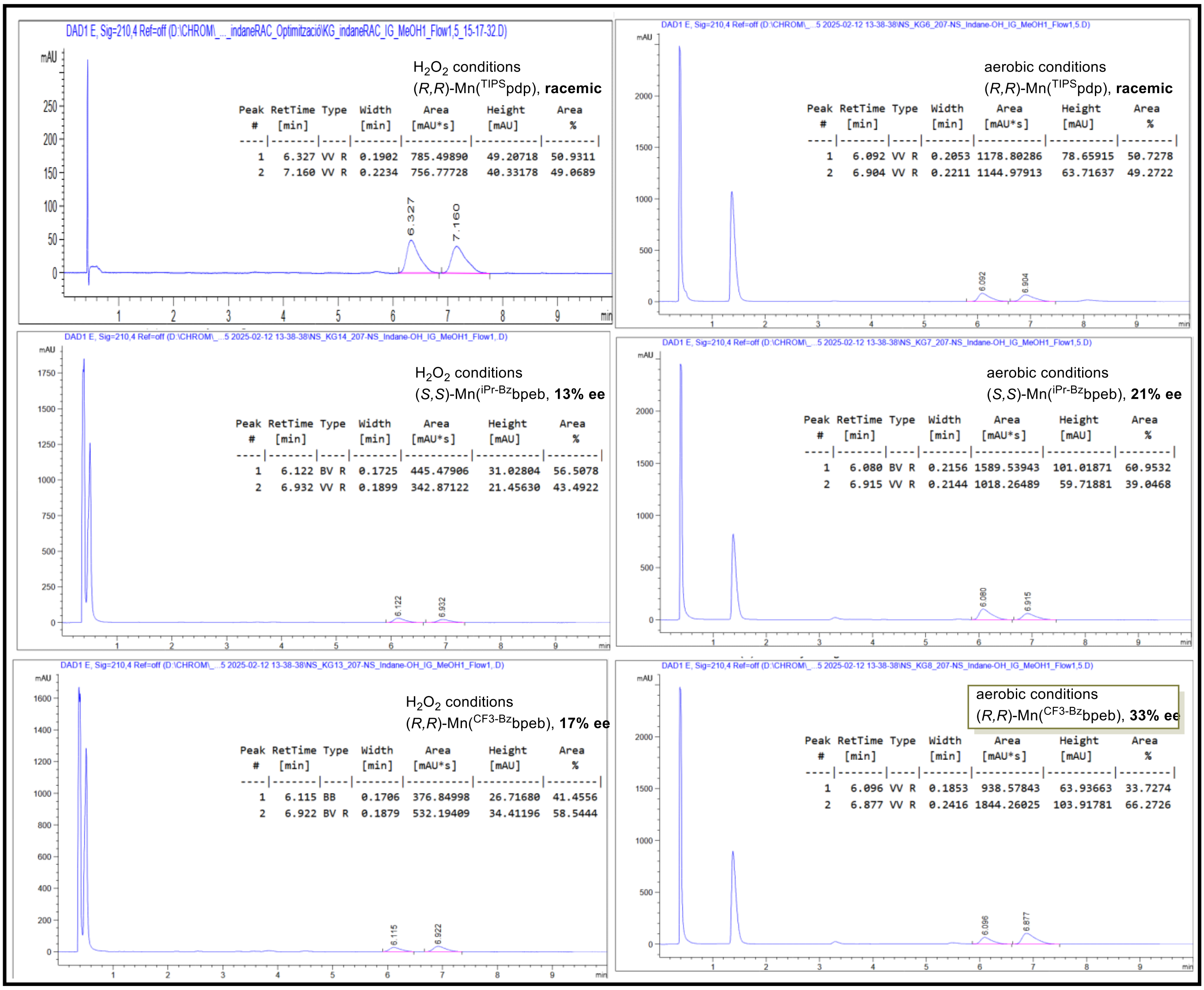

Scheme 14. SFC analysis to measure ee% for product **SI-13**. The best results are highlighted with a green frame. Conditions used: *aerobic conditions* refer to substrate (0.05 mmoles), catalyst (1 mol%), DKP (10 mol%), HE (10 equiv.), PA (1%), premixing of acid and catalyst, 10h addition, HFIP, rt stirring overnight under dioxygen atmosphere; *H_2O_2 conditions* refer to substrate (0.05 mmoles), catalyst (1 mol%), H_2O_2 (0.5 equiv.), PA (1%), HFIP, 0°C to r.t. for 1h.

(4-isopropylphenyl)methanol (**SI-14**)

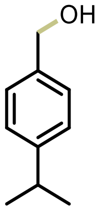

Isolated with (*R*,*R*)-Mn(^{TIPS}pdp) in a 0.15 mmol scale. Purification by flash chromatography (SiO_2, eluent: hexane/EtOAc 4:1, TLC: R_f = 0.33 upon hexane/EtOAc 4:1, UV active on TLC, stains blue-black upon *seebach* staining) affords the product as a colourless oil (Method A: 9.9 mg, 44% yield, Method B: -). Spectral data match those previously reported.[31] ^{1}H NMR (500 MHz, $CDCl_3$): δ_H 7.30 (d, *J* = 8.0 Hz, 2H), 7.24 (d, *J* = 8.0 Hz, 2H), 4.65 (s, 2H), 2.92 (dt, *J* = 13.8, 6.9 Hz, 1H), 1.26 (d, *J* = 6.9 Hz, 6H) ppm;^{13}C NMR (125 MHz, $CDCl_3$): δ_C 148.5, 138.3, 127.2, 126.6, 65.3, 33.9, 24.0 ppm.

<u>*Notes*</u>

- Substrate **SI-14** was successfully scaled up to 0.5 mmoles without any impact on its yield**,** using Method A (for more details, please see notes of substrate **SI-12**).

1-phenylethan-1-ol (**SI-15**)

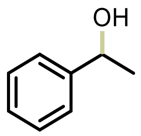

Isolated with (*R*,*R*)-Mn(^{TIPS}pdp) in a 0.1 mmol scale. Purification by flash chromatography (SiO_2, eluent: hexane/EtOAc 4:1, TLC: R_f = 0.37 upon hexane/EtOAc 4:1, UV active on TLC, stains blue upon *seebach* staining) affords the product as a colourless oil (Method A: 6 mg, 50% yield, 31% ee, Method B: 1.6 mg, 13% yield, 11% ee). Spectral data match those previously reported.[32] ^{1}H NMR (500 MHz, $CDCl_3$): δ_H 7.40 – 7.34 (m, 4H), 7.32 – 7.26 (m, 1H), 4.92 – 4.85 (m, 1H), 2.21 (brs, 1H), 1.49 (d, *J* = 6.5 Hz, 3H) ppm; ^{13}C NMR (125 MHz, $CDCl_3$): δ_C 145.8, 128.5, 127.4, 125.4, 70.4, 25.2 ppm.

Notes

- Substrate **SI-15** was successfully scaled up to 0.5 mmoles without any impact on its yield, using Method A (for more details, please see notes of substrate **SI-12**).

- For the SFC analysis: Chiralpak IG, CO_2: EtOH 95:5, 35 ºC, flow rate = 1.0 mL/min, λ = 210 nm. The analysis time was 4 minutes. t1 = 1.81 min, t2 = 1.99 min.

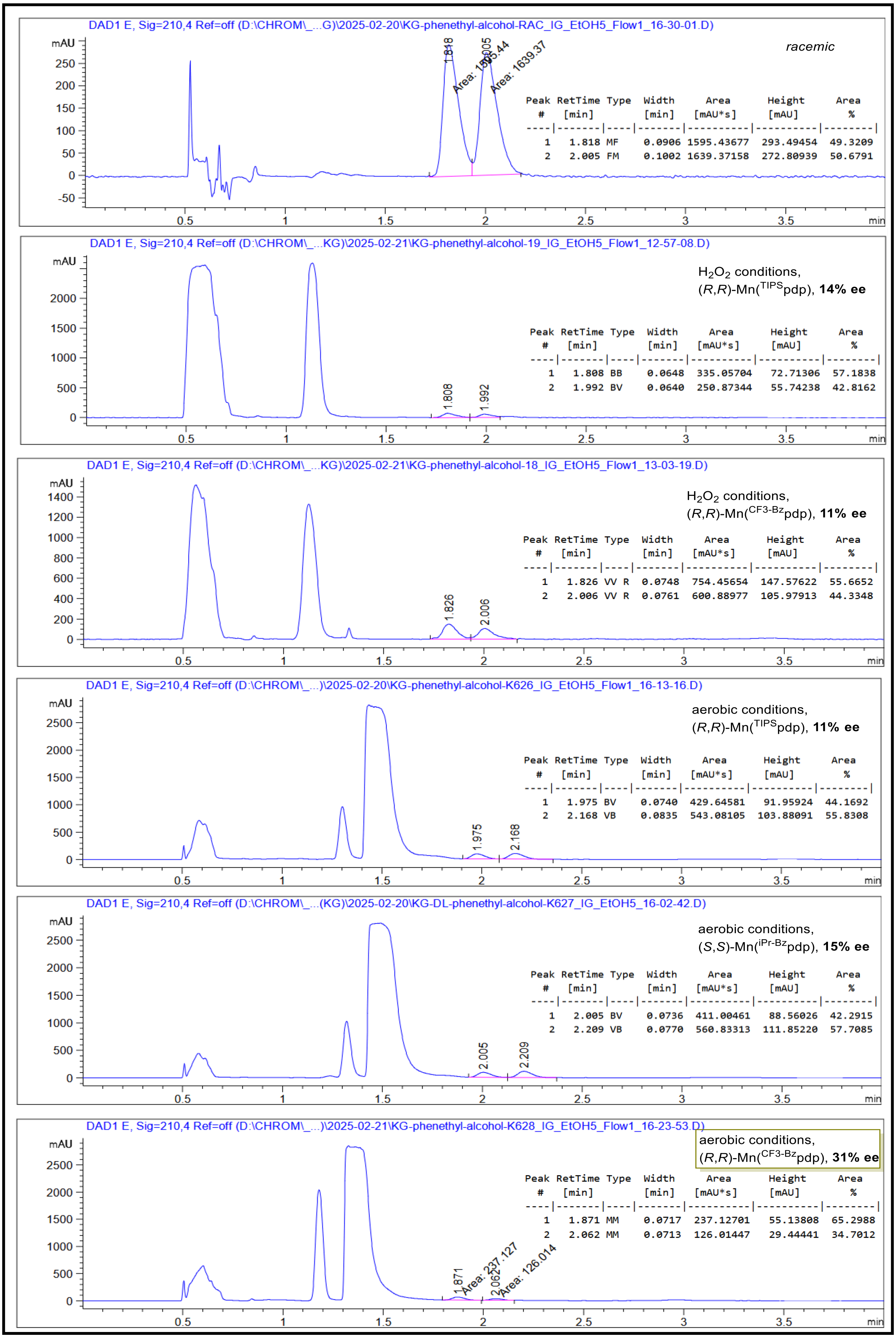

Scheme 15. SFC analysis to measure ee% for product **SI-15**. The best results are highlighted with a green frame. Conditions used: *aerobic conditions* refer to substrate (0.05 mmoles), catalyst (1 mol%), DKP (10 mol%), HE (10 equiv.), PA (1%), premixing of acid and catalyst, 10h addition, HFIP, rt stirring overnight under dioxygen atmosphere; *H_2O_2 conditions* refer to substrate (0.05 mmoles), catalyst (1 mol%), H_2O_2 (0.5 equiv.), PA (1%), HFIP, 0°C to r.t. for 1h.

7. NMR Spectra

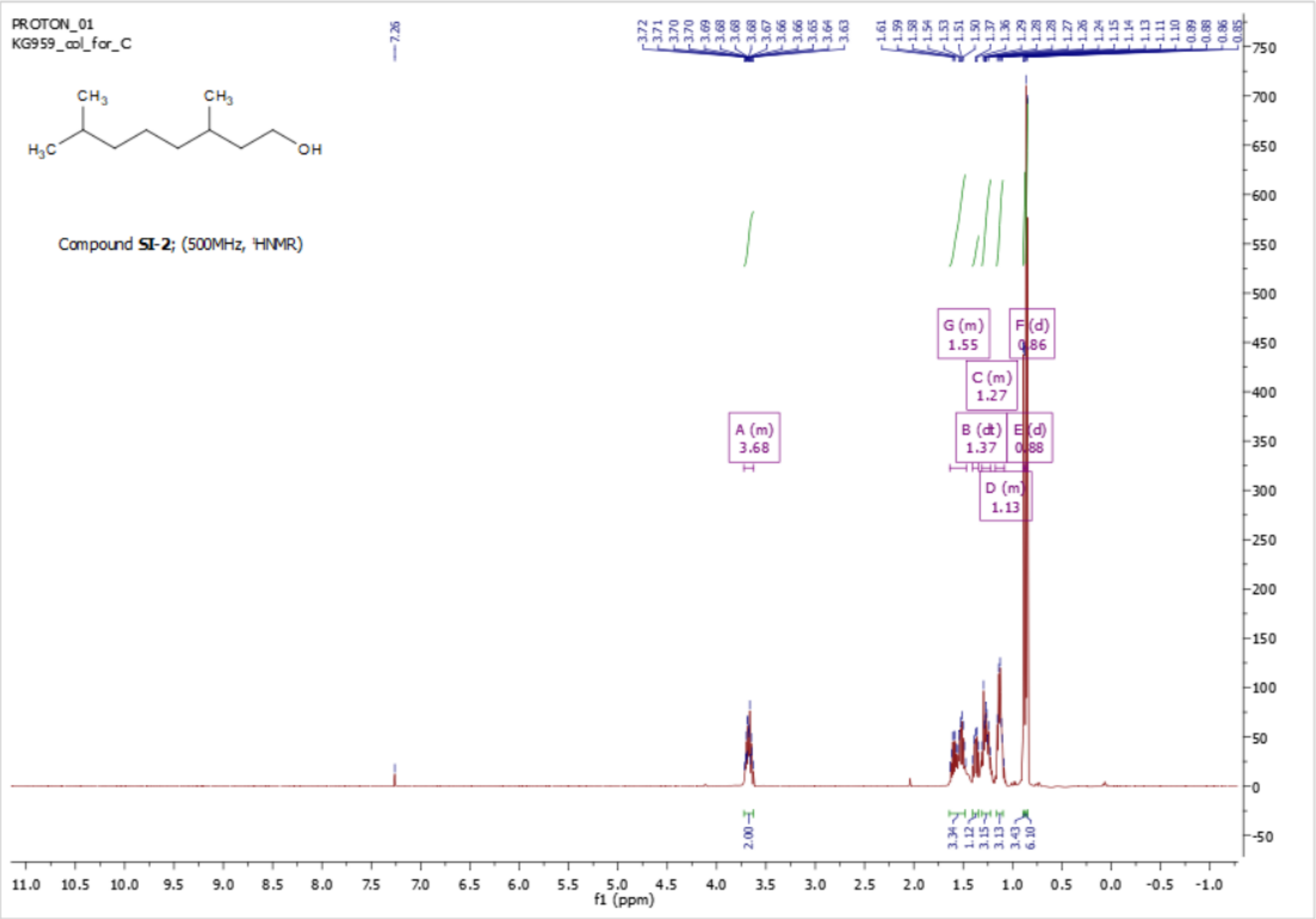

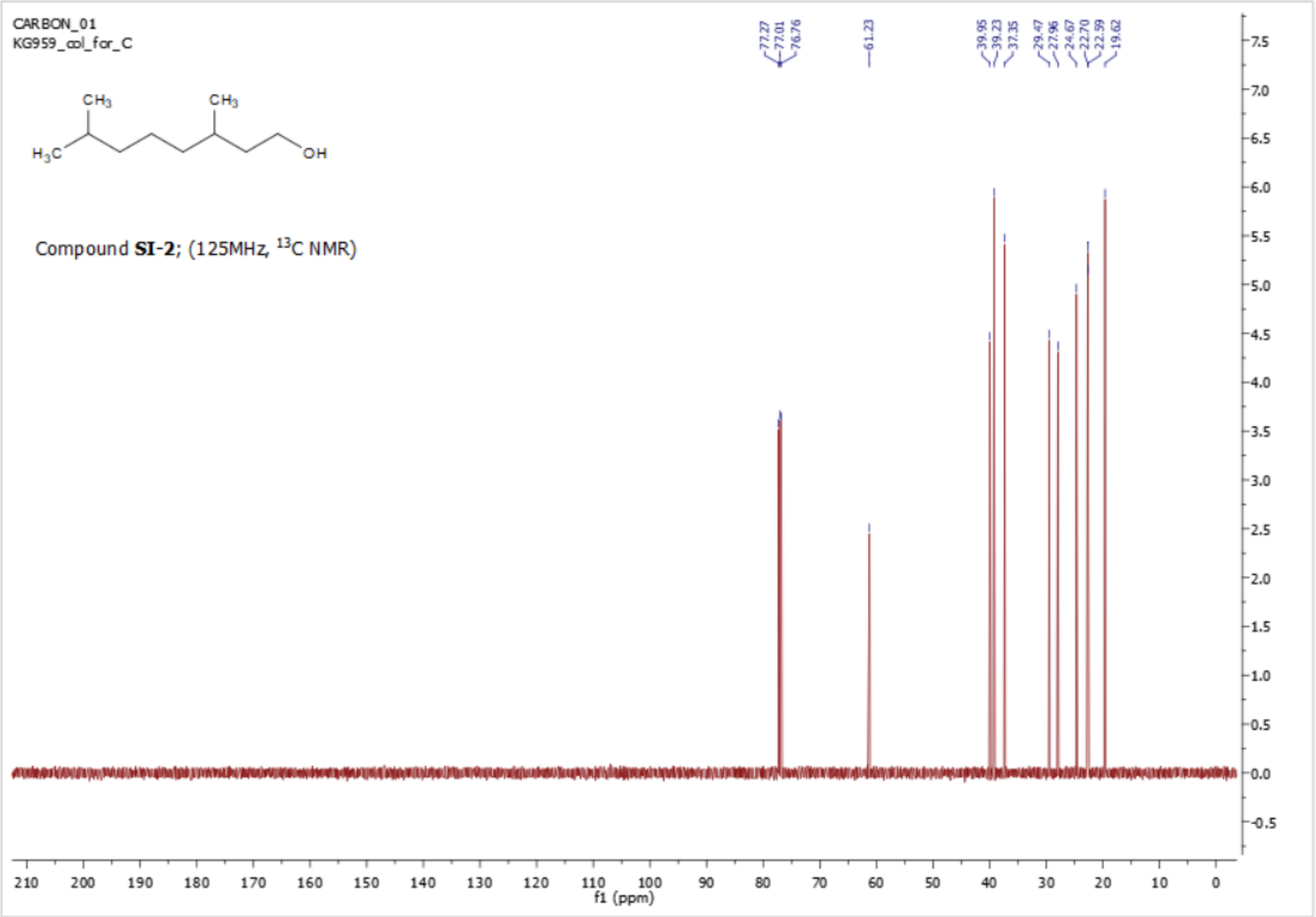

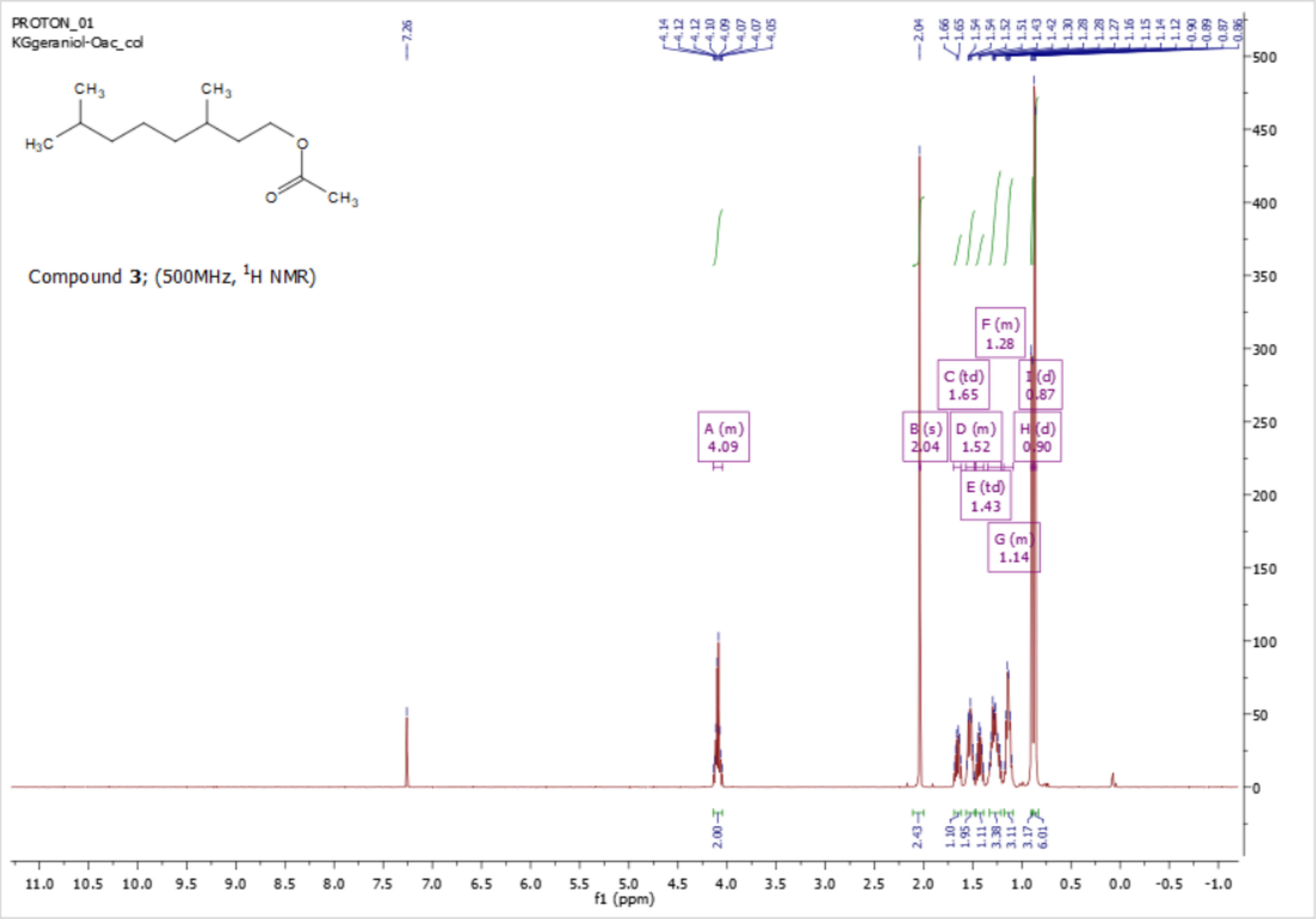
PROTON_01
KGgeraniol-Oac_col
Compound 3; (500MHz, 1H NMR)
A (m) 4.09
B (s) 2.04
C (td) 1.65
D (m) 1.52
E (td) 1.43
F (m) 1.28
G (m) 1.14
H (d) 0.90
I (d) 0.87
f1 (ppm)

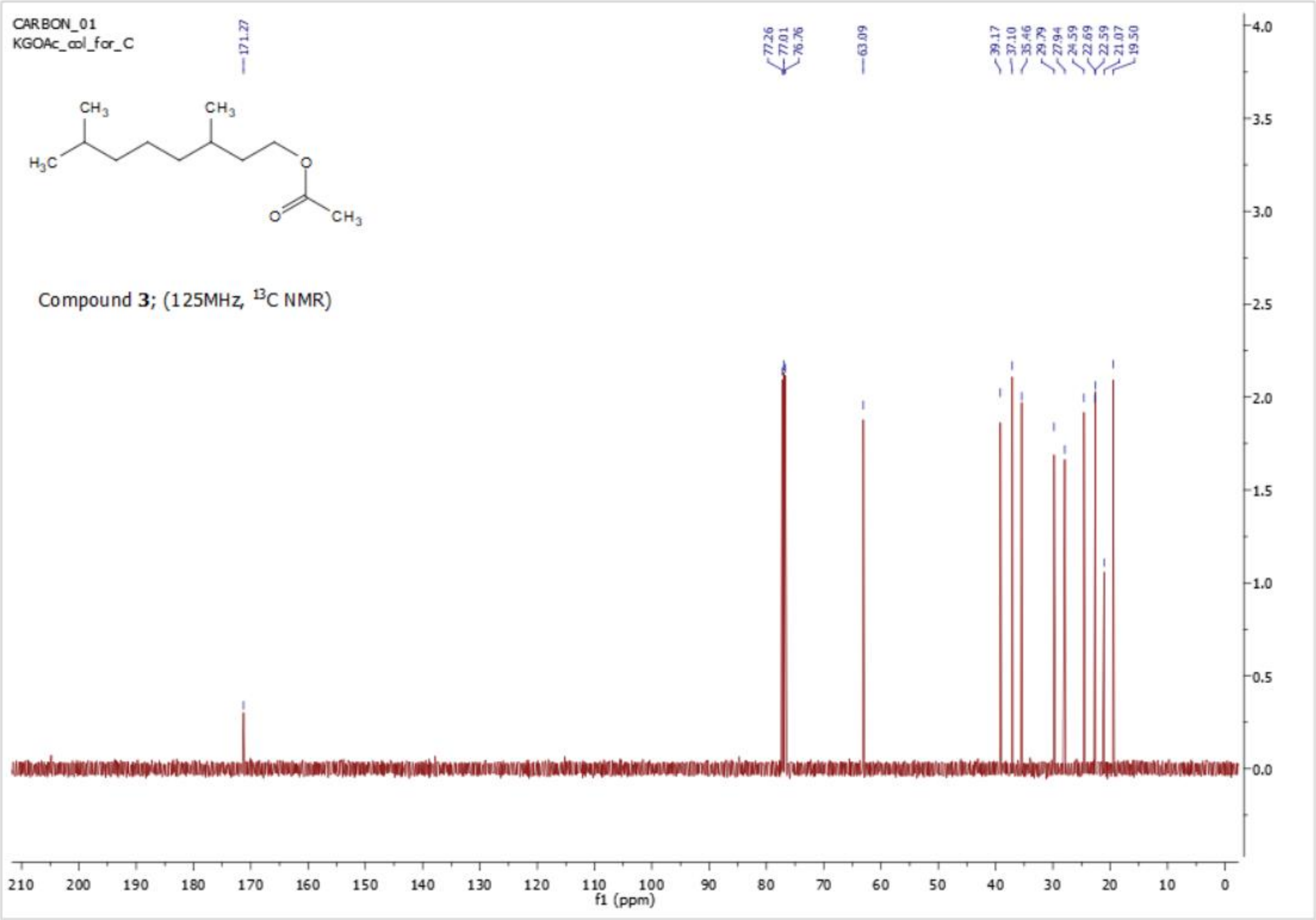
CARBON_01
KGOAc_col_for_C
171.27
77.26
77.01
76.76
63.09
39.17
37.10
35.46
29.79
27.94
24.59
22.69
22.59
21.07
19.50
Compound 3; (125MHz, 13C NMR)
f1 (ppm)

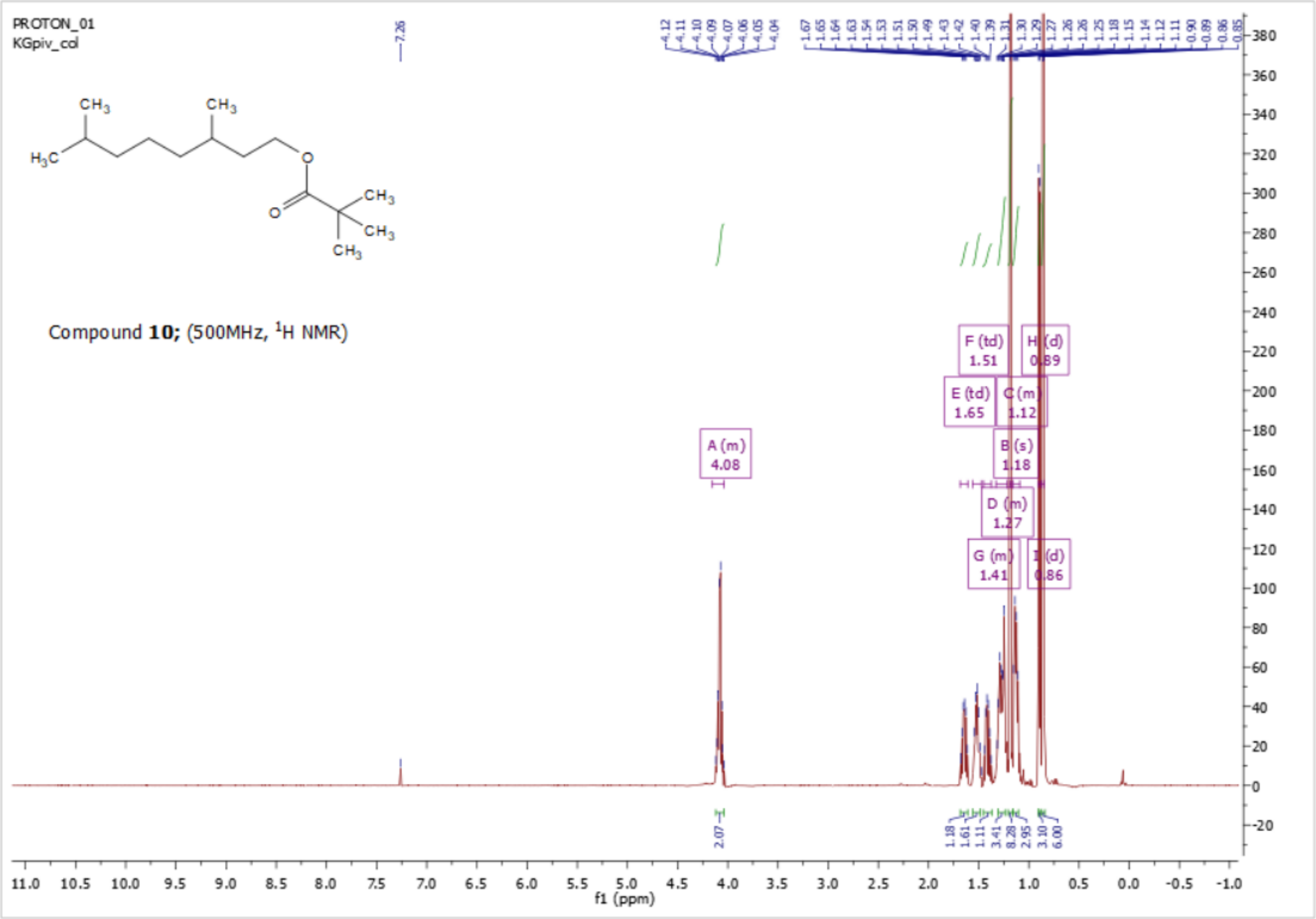
PROTON_01
KGpiv_col
Compound 10; (500MHz, 1H NMR)
f1 (ppm)

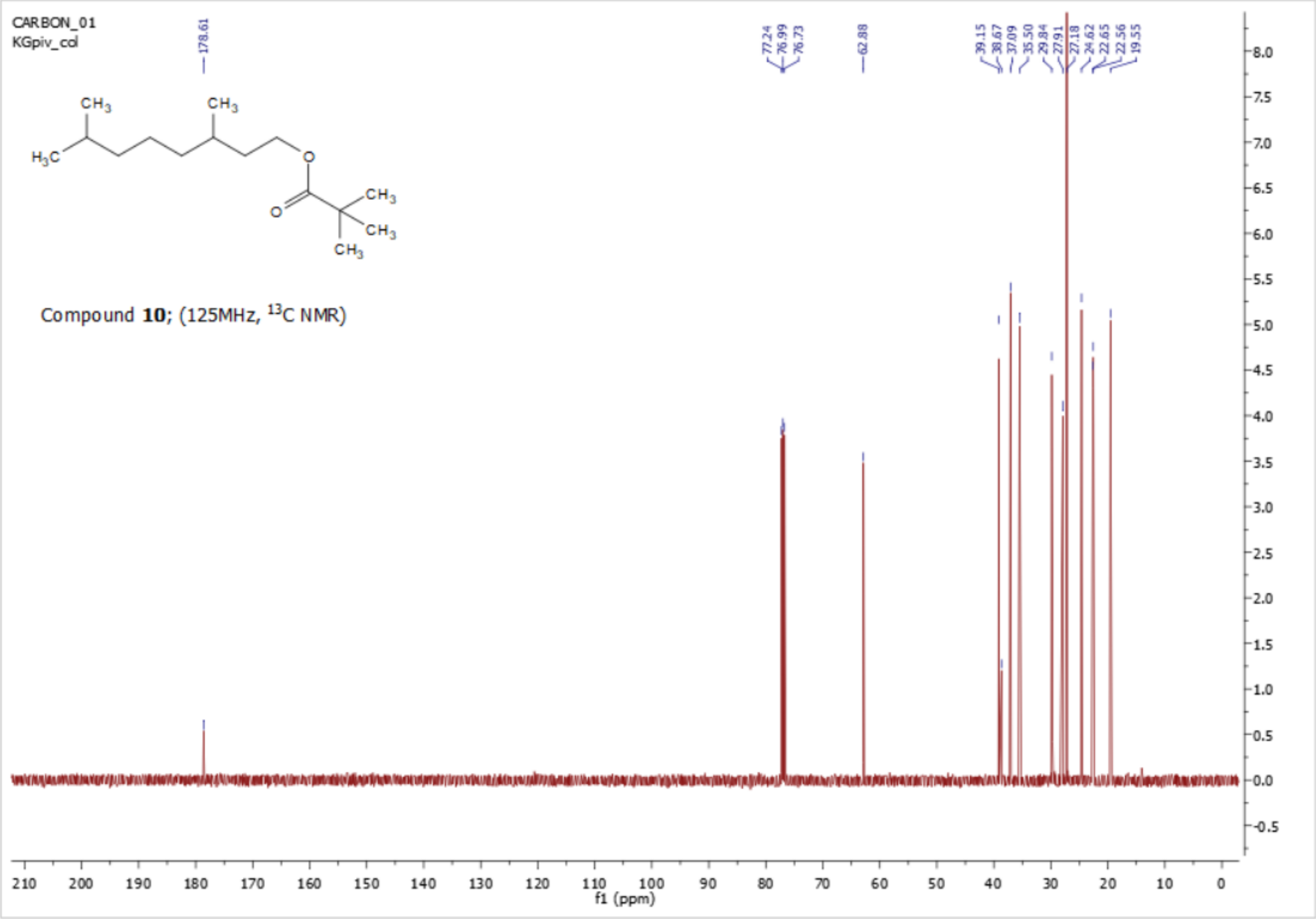
CARBON_01
KGpiv_col
Compound 10; (125MHz, 13C NMR)
f1 (ppm)

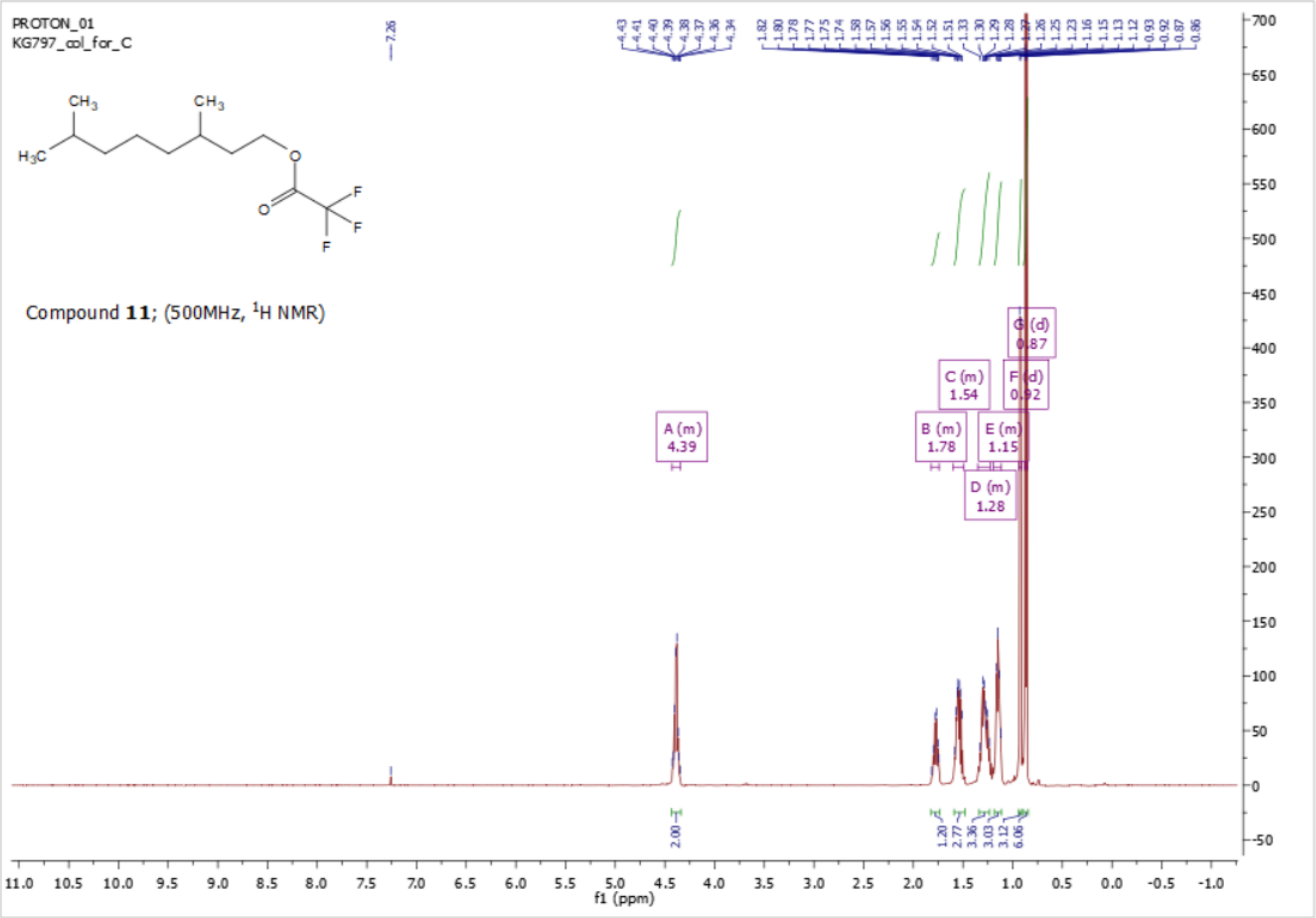
PROTON_01
KG797_col_for_C
Compound **11**; (500MHz, ^{1}H NMR)
A (m) 4.39
B (m) 1.78
C (m) 1.54
D (m) 1.28
E (m) 1.15
F (d) 0.92
G (d) 0.87
f1 (ppm)

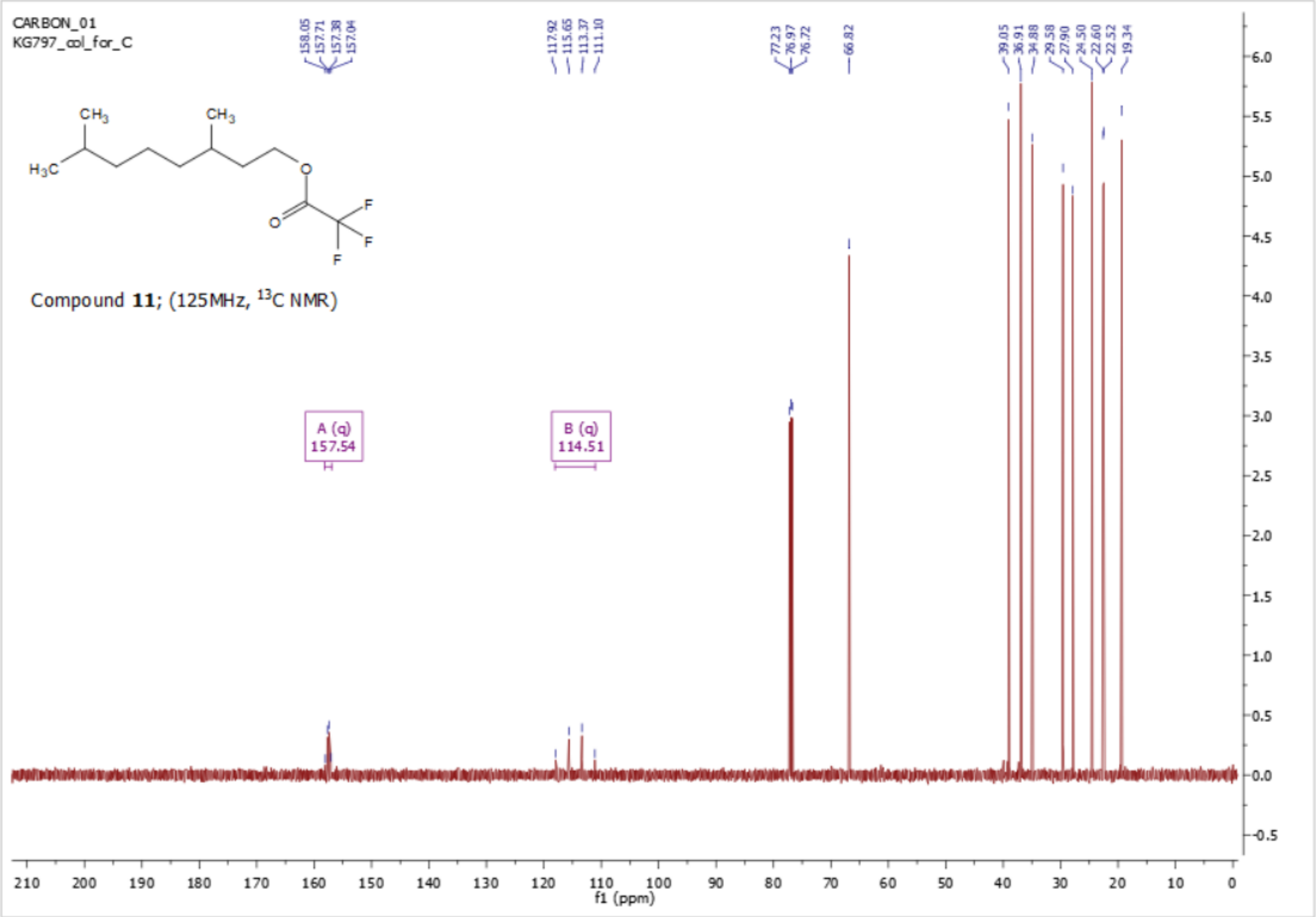
CARBON_01
KG797_col_for_C
Compound **11**; (125MHz, ^{13}C NMR)
A (q) 157.54
B (q) 114.51
f1 (ppm)

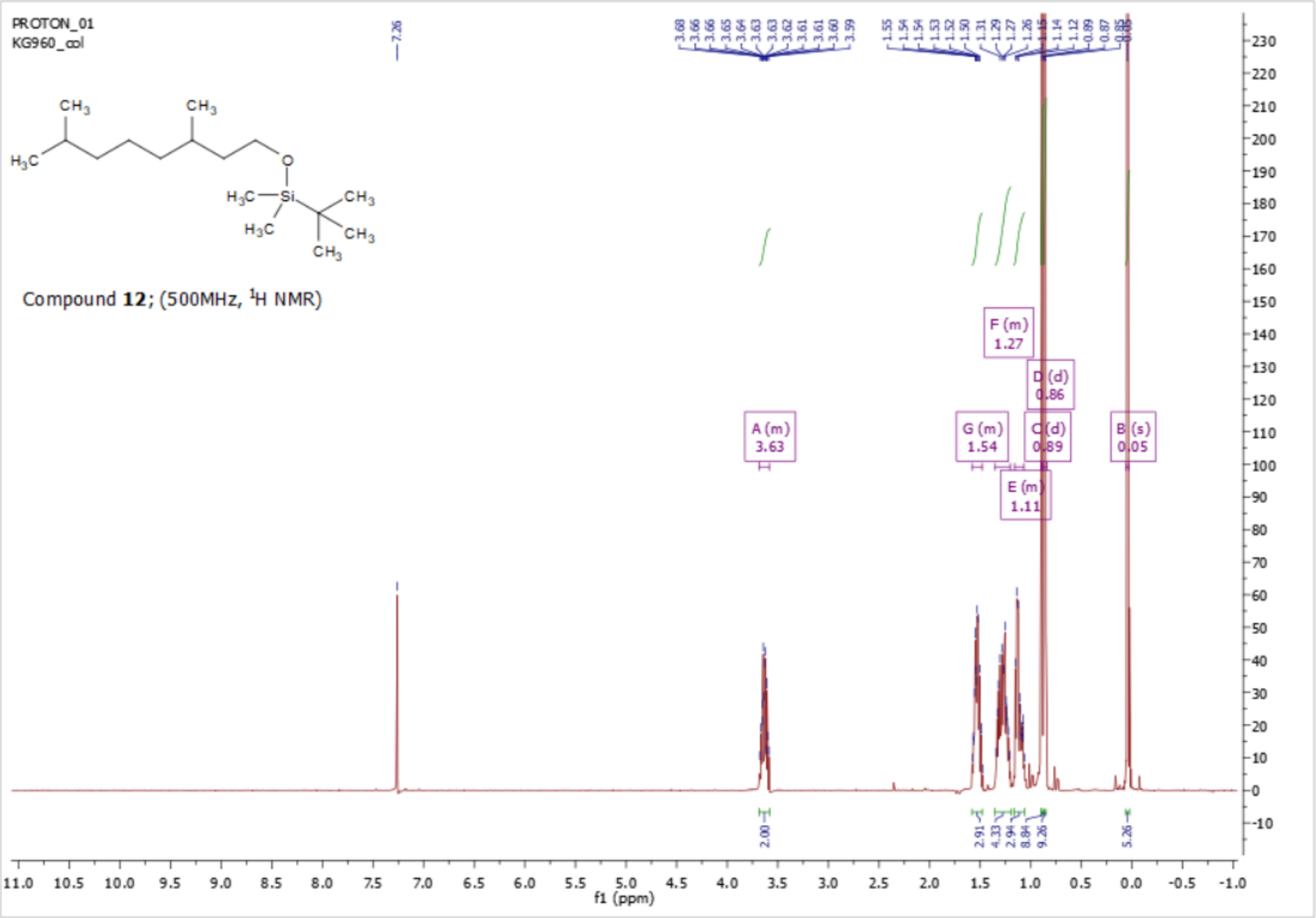
PROTON_01
KG960_col
Compound 12; (500MHz, 1H NMR)
A (m) 3.63
B (s) 0.05
C (d) 0.89
D (d) 0.86
E (m) 1.11
F (m) 1.27
G (m) 1.54
7.26
2.00
2.91
4.33
2.94
8.84
9.26
5.26
f1 (ppm)

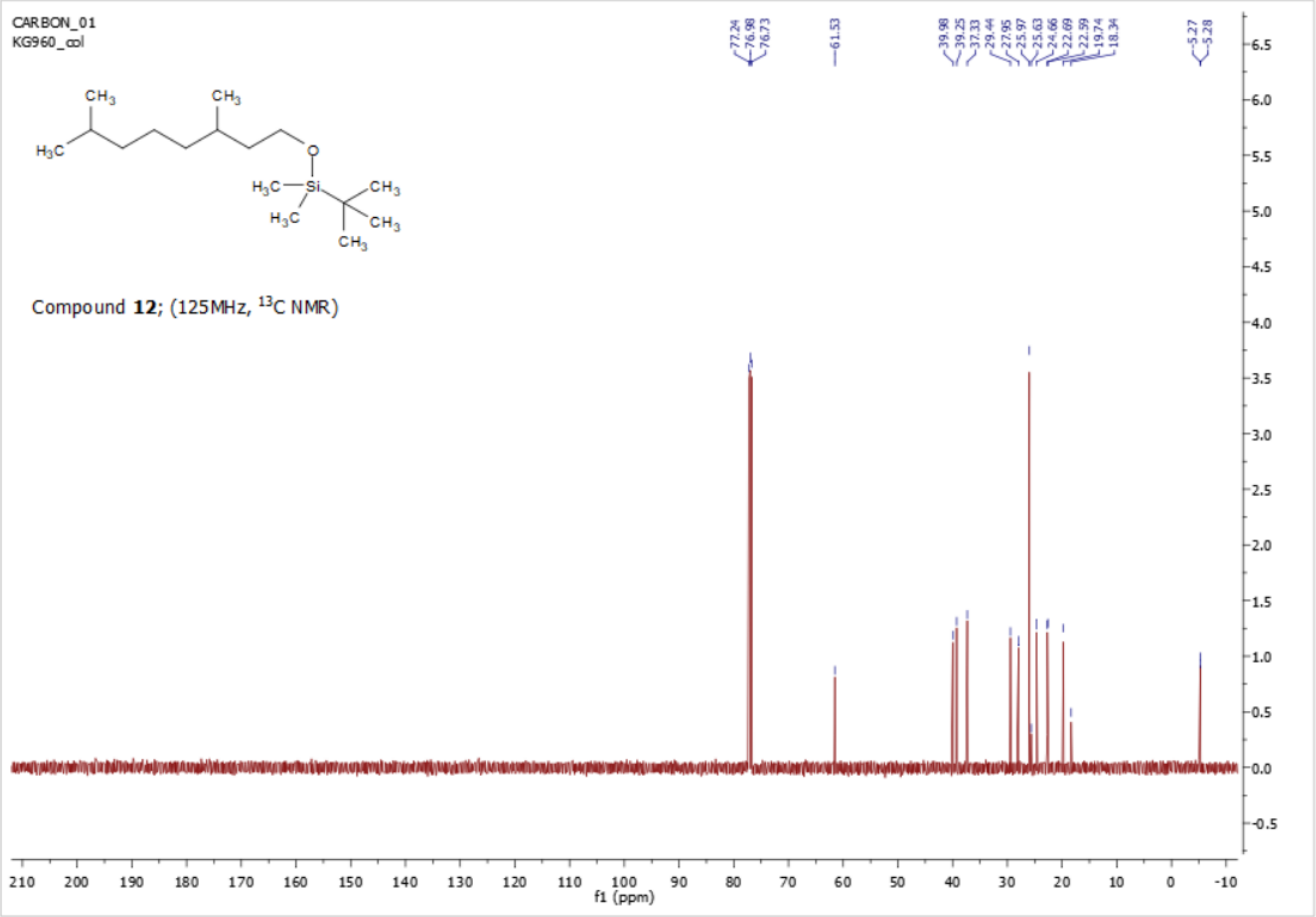
CARBON_01
KG960_col
Compound 12; (125MHz, 13C NMR)
77.24
76.98
76.73
61.53
39.98
39.25
37.33
29.44
27.95
25.97
25.63
24.66
22.69
22.59
19.74
18.34
-5.27
-5.28
f1 (ppm)

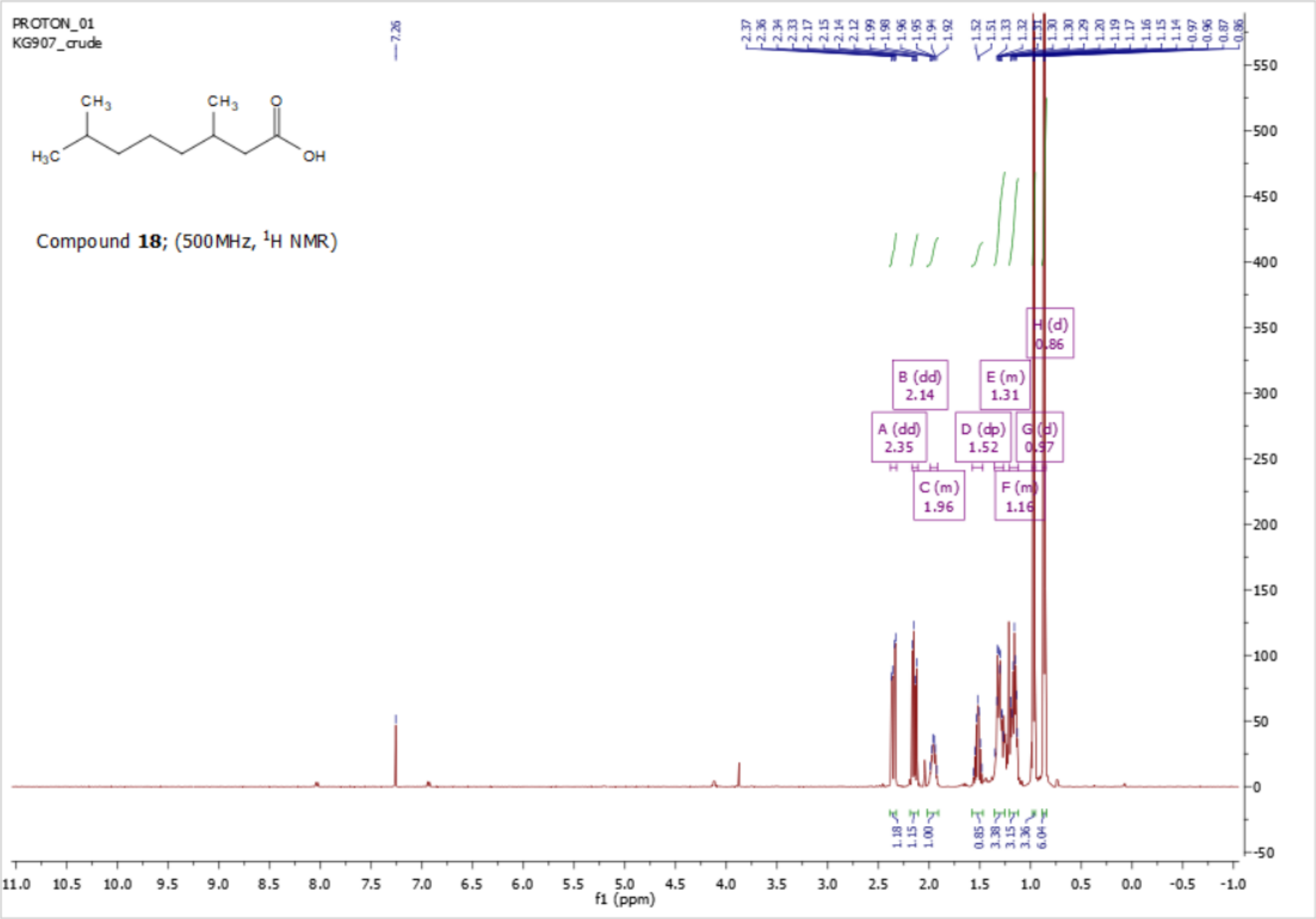
PROTON_01
KG907_crude
Compound 18; (500MHz, ¹H NMR)
f1 (ppm)

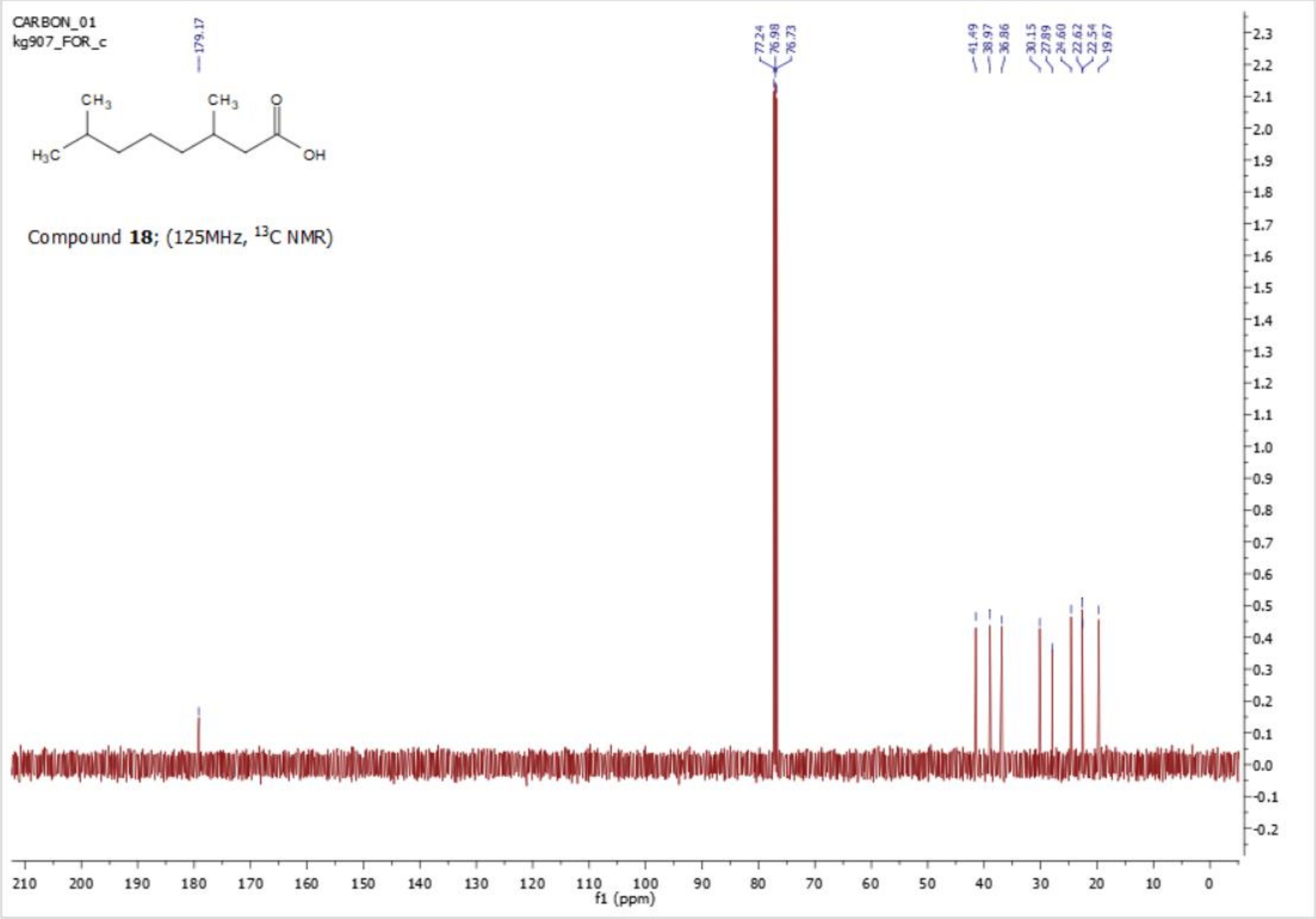
CARBON_01
kg907_FOR_c
Compound 18; (125MHz, ¹³C NMR)
f1 (ppm)

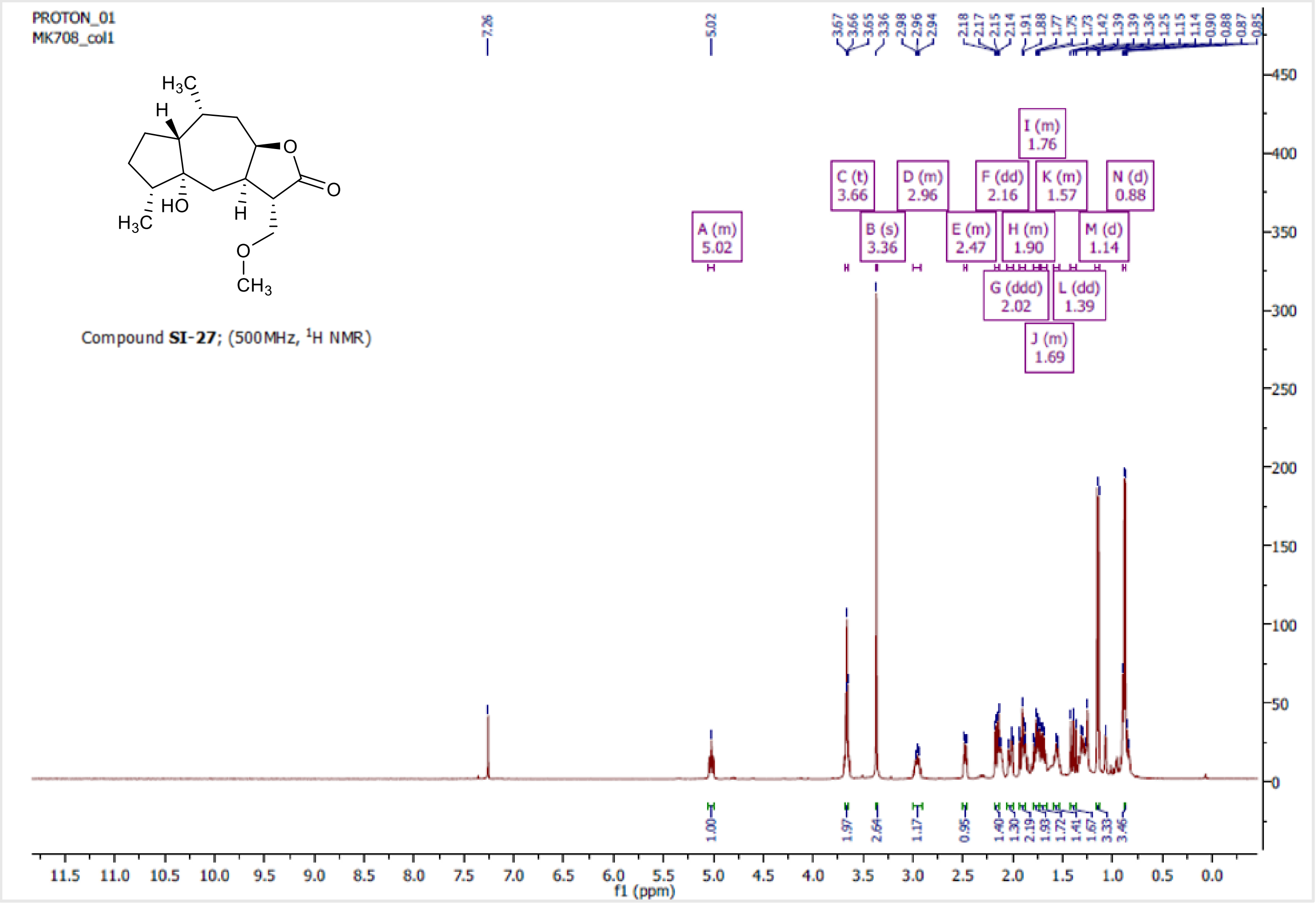
PROTON_01
MK708_col1
7.26
5.02
3.67 3.66 3.65 3.36 2.98 2.96 2.94 2.18 2.17 2.15 2.14 1.91 1.88 1.77 1.75 1.73 1.42 1.39 1.39 1.36 1.25 1.15 1.14 0.90 0.88 0.87 0.85
I (m) 1.76
C (t) 3.66
D (m) 2.96
F (dd) 2.16
K (m) 1.57
N (d) 0.88
A (m) 5.02
B (s) 3.36
E (m) 2.47
H (m) 1.90
M (d) 1.14
G (ddd) 2.02
L (dd) 1.39
J (m) 1.69
1.00 1.97 2.64 1.17 0.95 1.40 1.30 2.19 1.93 1.72 1.41 1.67 3.33 3.46
f1 (ppm)
H$_3$C
H
O
O
HO
H
H$_3$C
O
CH$_3$
Compound SI-27; (500MHz, ^{1}H NMR)

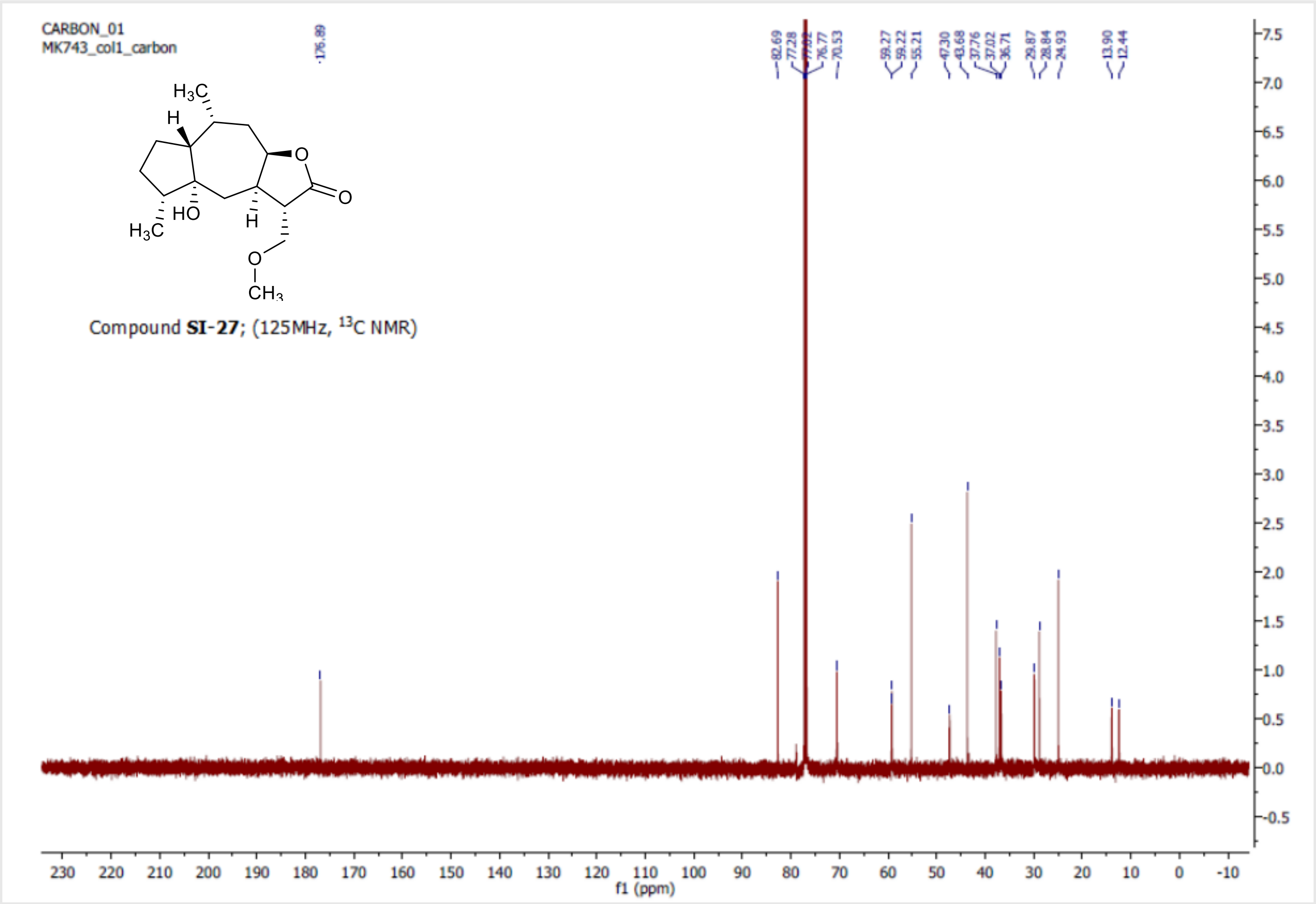
CARBON_01
MK743_col1_carbon
176.89
82.69 77.28 77.02 76.77 70.53 59.27 59.22 55.21 47.30 43.68 37.76 37.02 36.71 29.87 28.84 24.93 13.90 12.44
f1 (ppm)
H$_3$C
H
O
O
HO
H
H$_3$C
O
CH$_3$
Compound SI-27; (125MHz, ^{13}C NMR)

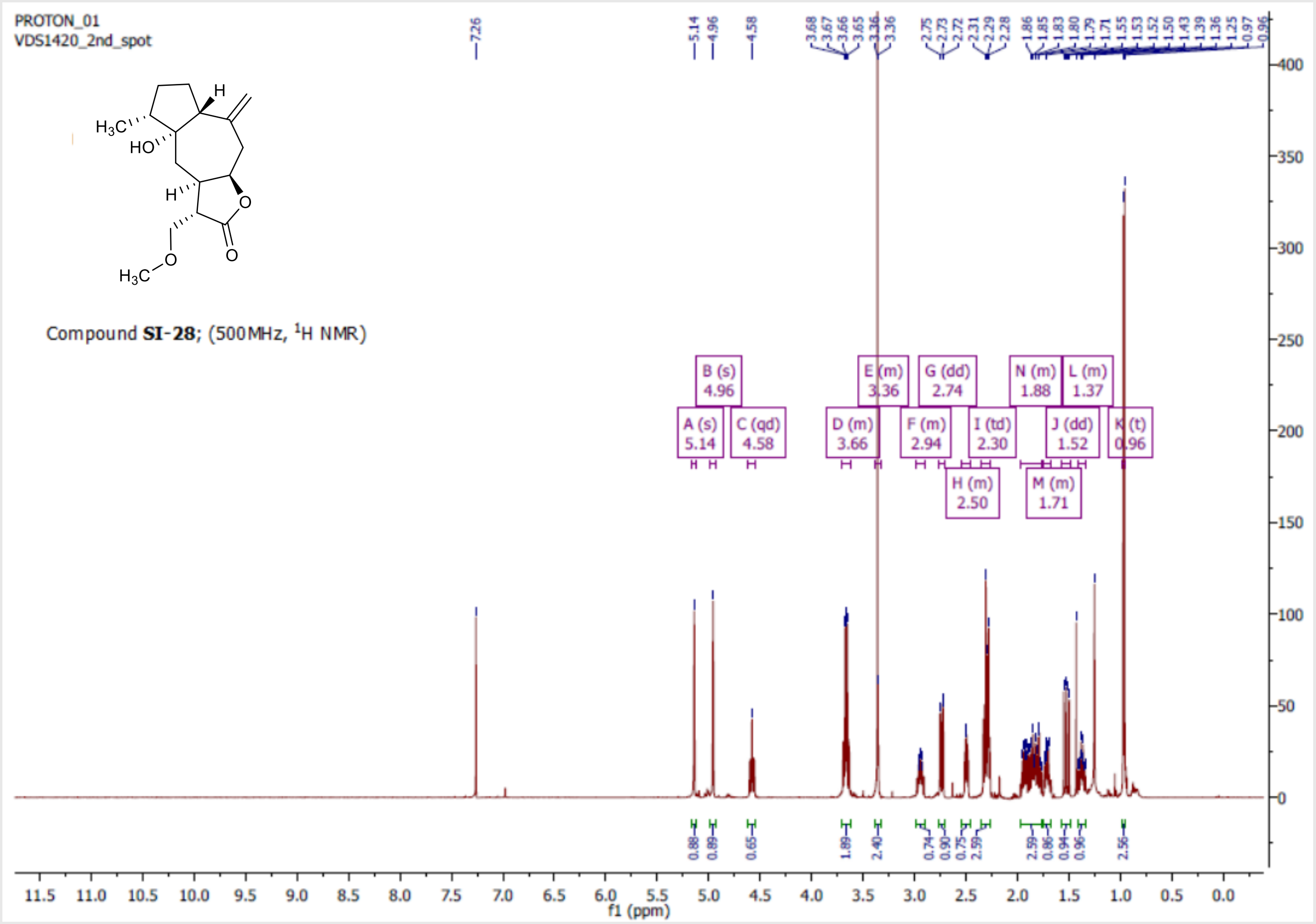
PROTON_01
VDS1420_2nd_spot
Compound SI-28; (500MHz, 1H NMR)
f1 (ppm)

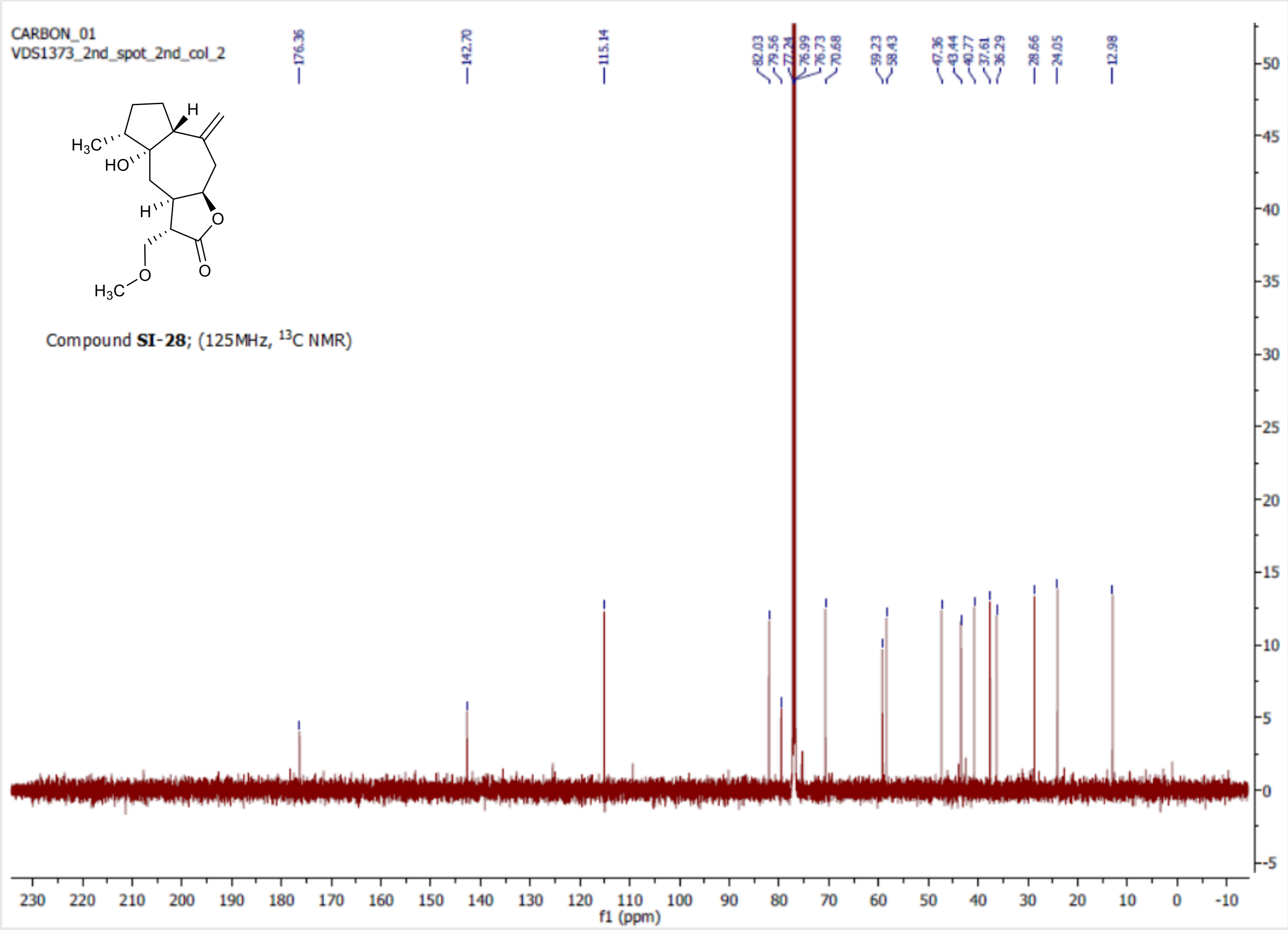
CARBON_01
VDS1373_2nd_spot_2nd_col_2
Compound SI-28; (125MHz, 13C NMR)
f1 (ppm)

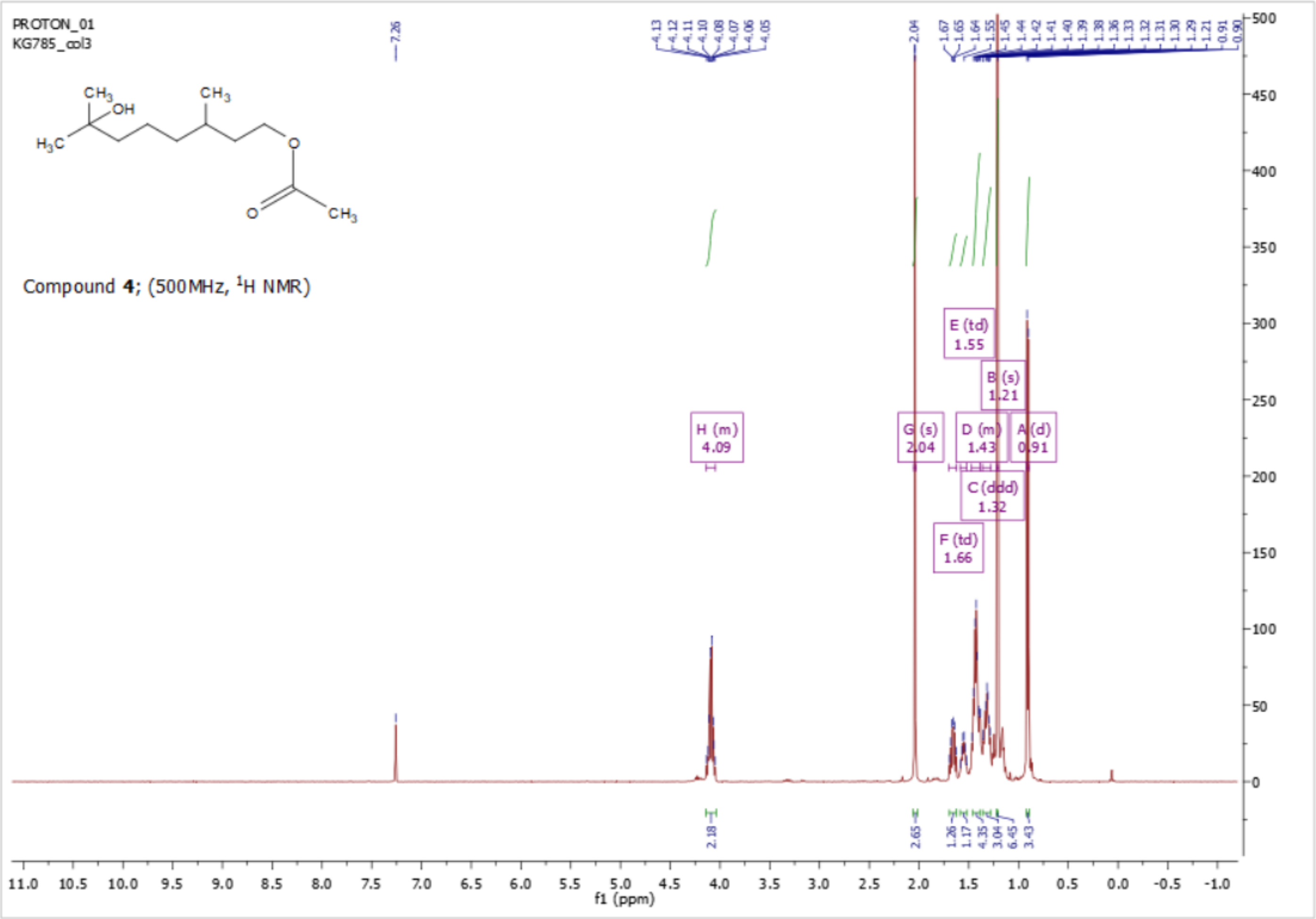
PROTON_01
KG785_cdcl3
Compound 4; (500MHz, 1H NMR)
H (m) 4.09
G (s) 2.04
E (td) 1.55
B (s) 1.21
D (m) 1.43
A (d) 0.91
C (ddd) 1.32
F (td) 1.66
f1 (ppm)

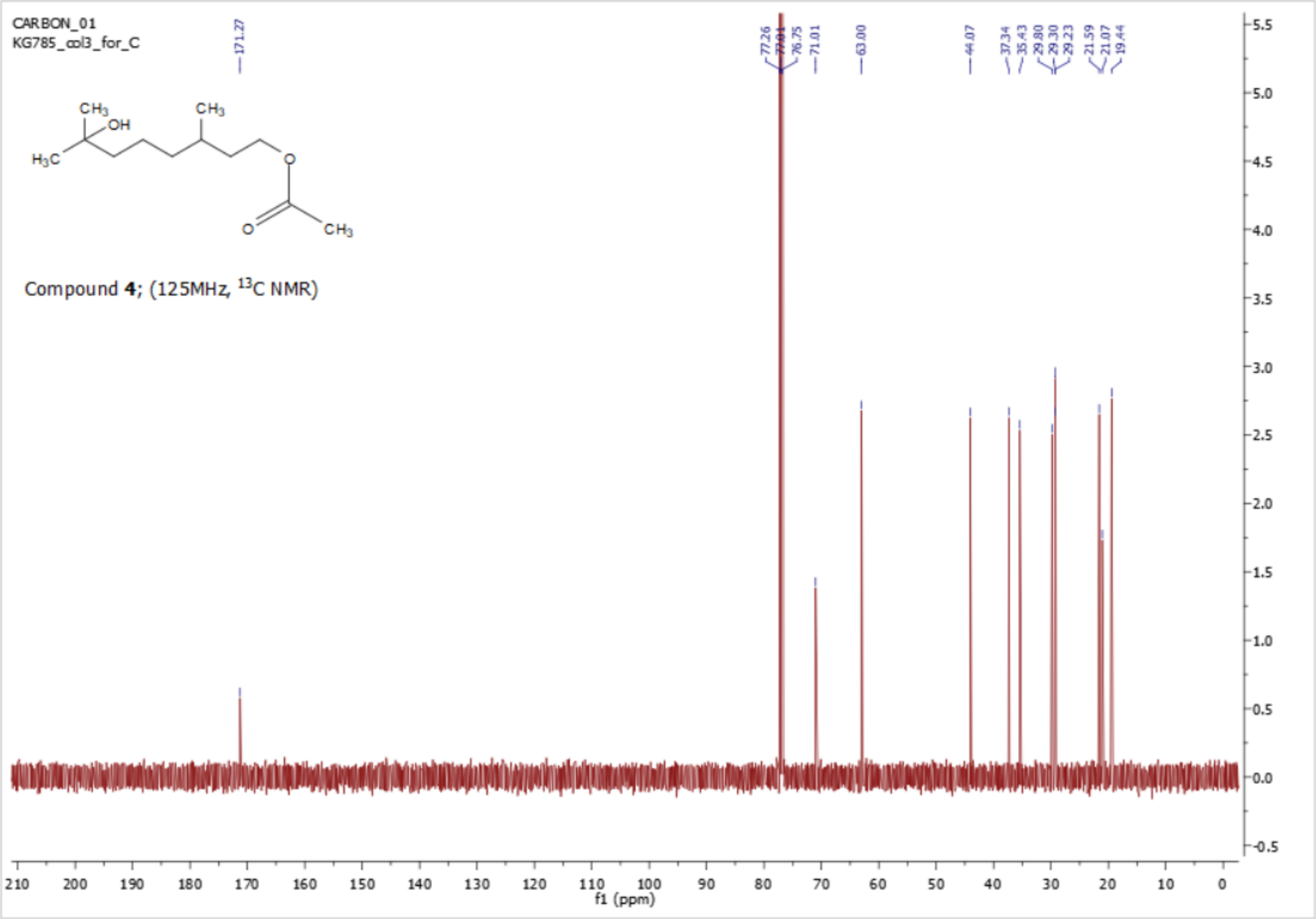
CARBON_01
KG785_cdcl3_for_C
Compound 4; (125MHz, 13C NMR)
171.27
77.26
77.01
76.75
71.01
63.00
44.07
37.34
35.43
29.80
29.30
29.23
21.59
21.07
19.44
f1 (ppm)

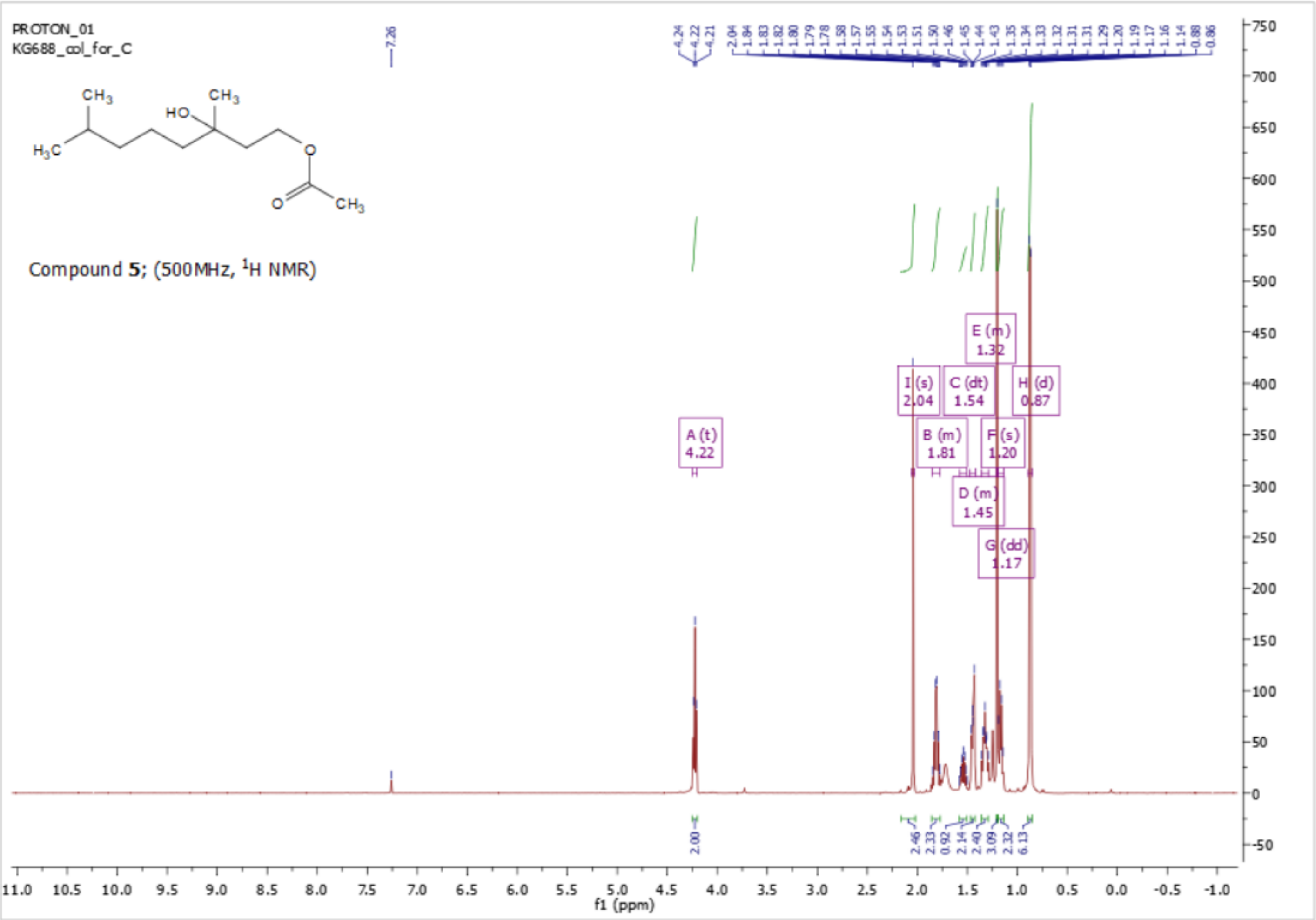
PROTON_01
KG688_col_for_C
Compound 5; (500MHz, 1H NMR)
7.26
4.24 4.22 4.21
2.04 1.84 1.83 1.82 1.80 1.79 1.78 1.58 1.57 1.55 1.54 1.53 1.51 1.50 1.46 1.45 1.44 1.43 1.35 1.34 1.33 1.32 1.31 1.31 1.29 1.20 1.19 1.17 1.16 1.14 0.88 0.86
A (t) 4.22
I (s) 2.04
B (m) 1.81
C (dt) 1.54
D (m) 1.45
E (m) 1.32
F (s) 1.20
G (dd) 1.17
H (d) 0.87
2.00
2.46 2.33 0.92 2.14 2.40 3.09 2.32 6.13
f1 (ppm)

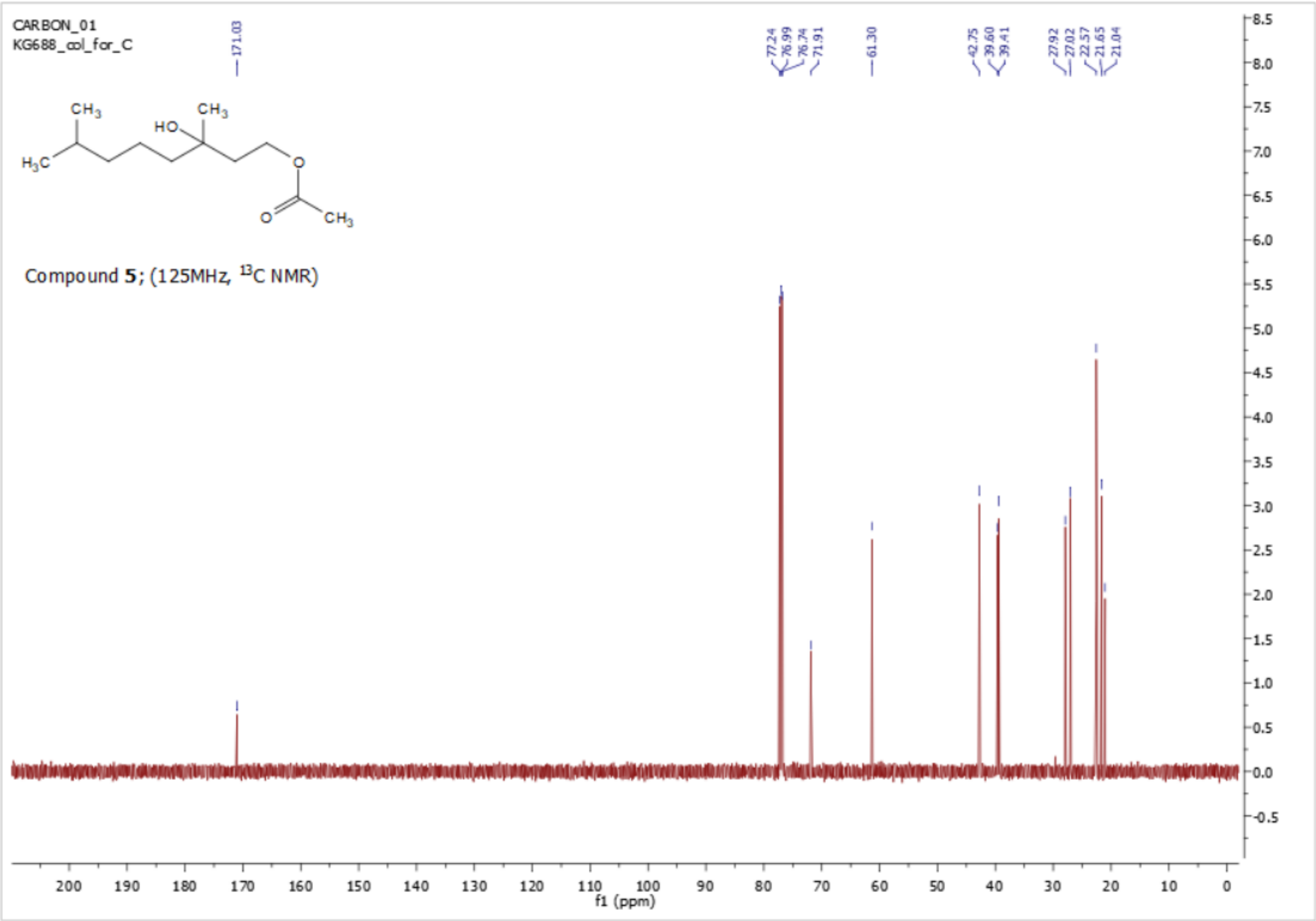
CARBON_01
KG688_col_for_C
Compound 5; (125MHz, 13C NMR)
171.03
77.24 76.99 76.74 71.91
61.30
42.75 39.60 39.41
27.92 27.02 22.57 21.65 21.04
f1 (ppm)

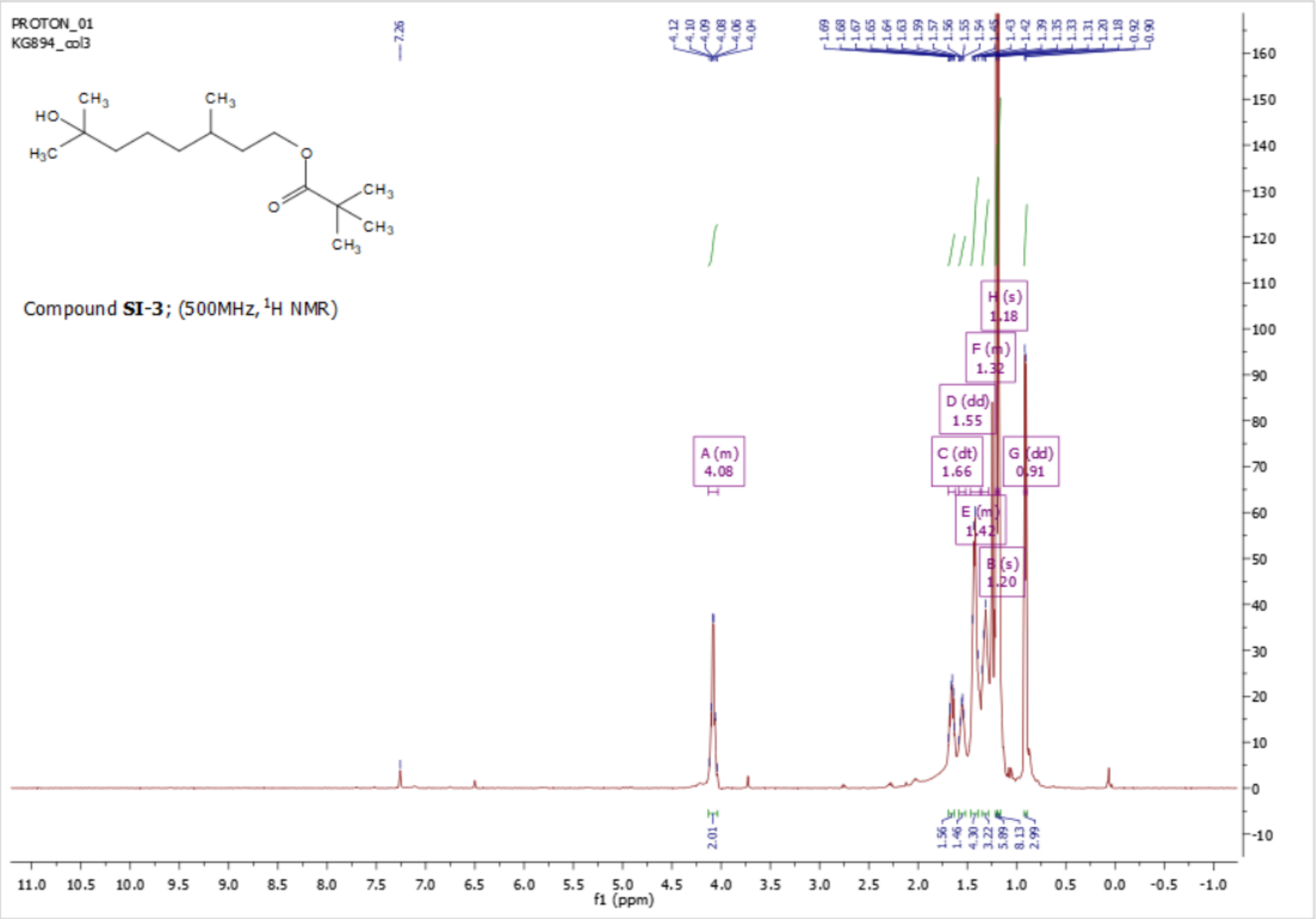
PROTON_01
KG894_cdl3
Compound SI-3; (500MHz, 1H NMR)
f1 (ppm)

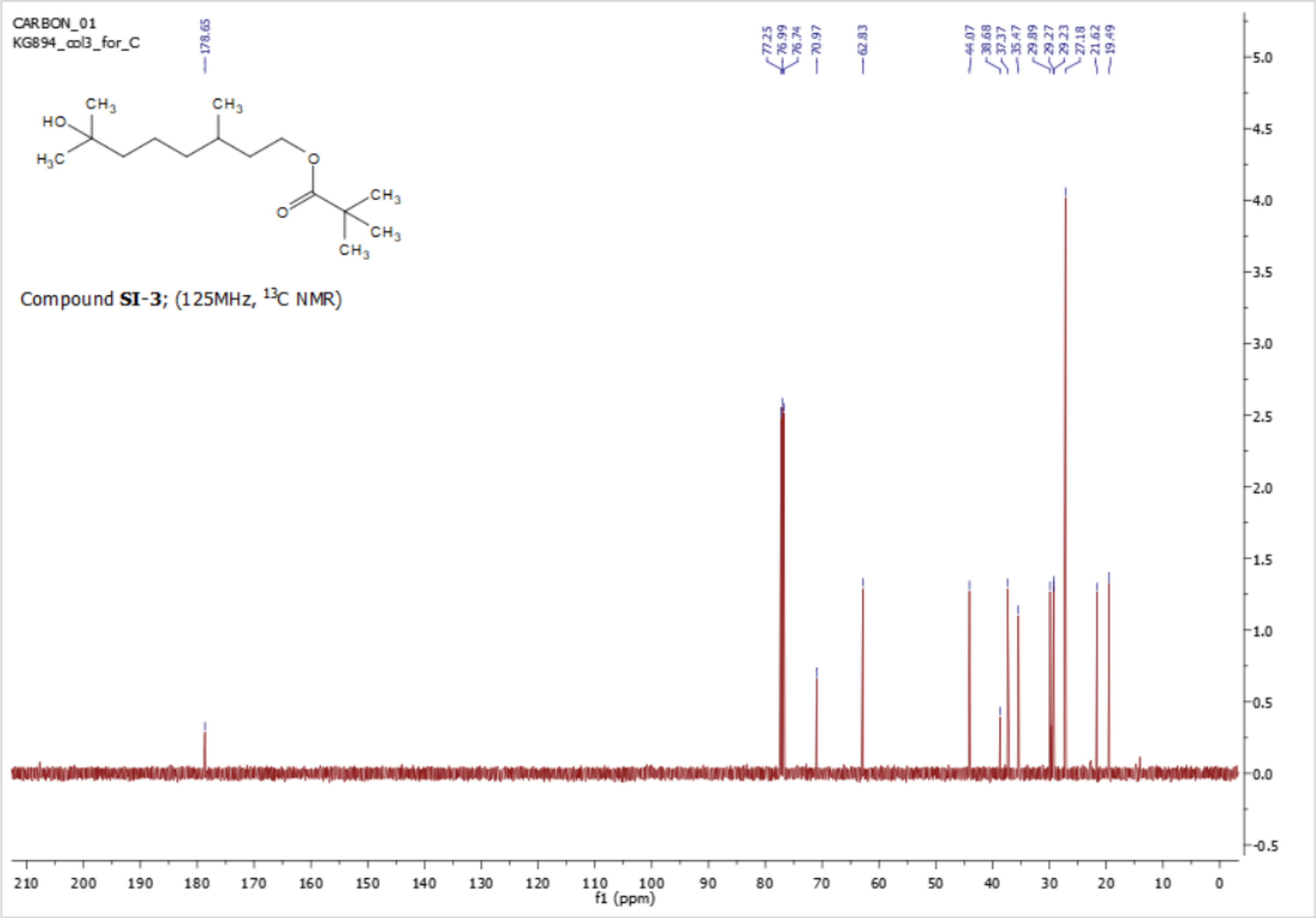
CARBON_01
KG894_cdl3_for_C
Compound SI-3; (125MHz, 13C NMR)
f1 (ppm)

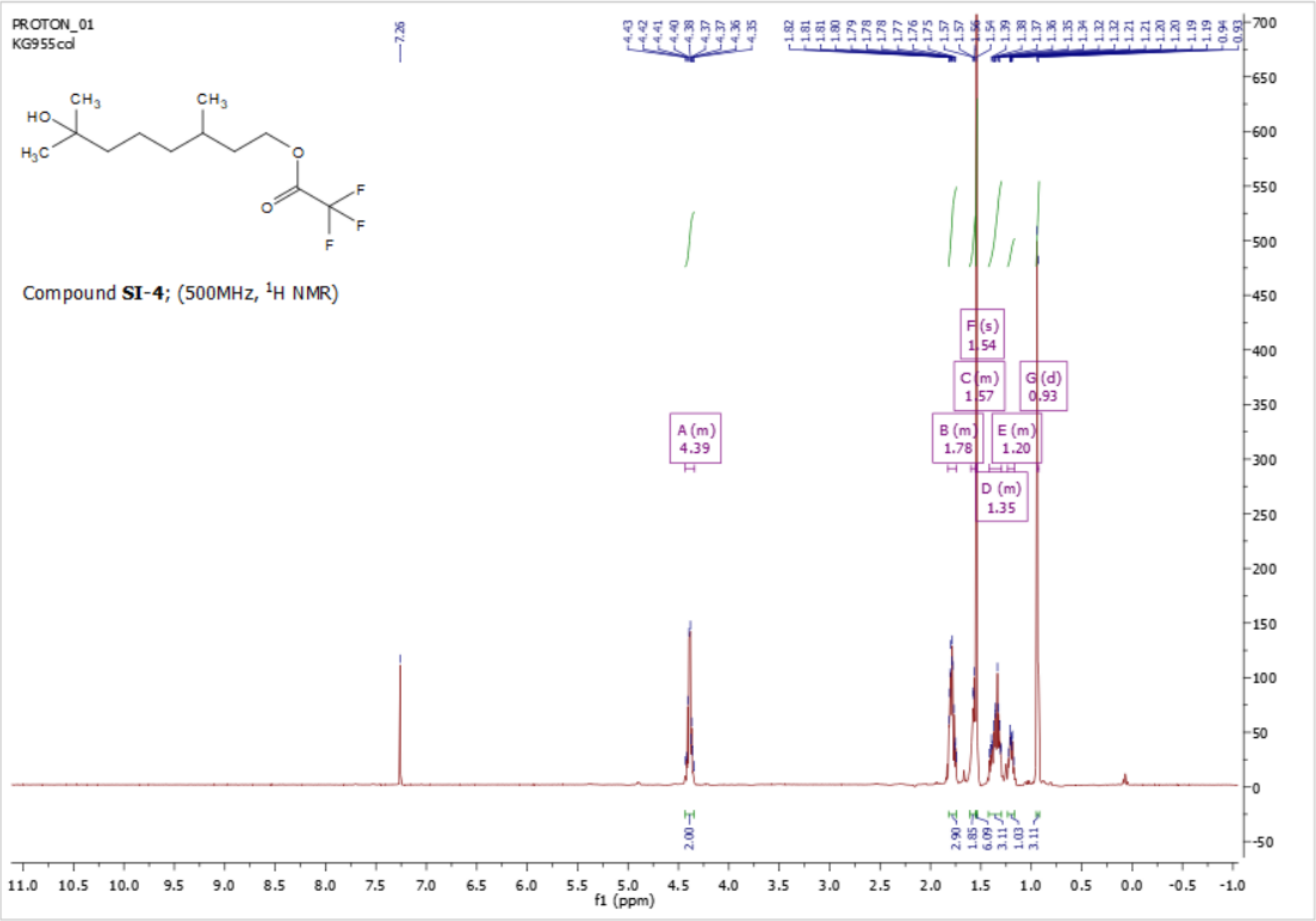
PROTON_01
KG955cdl
Compound SI-4; (500MHz, 1H NMR)
7.26
4.43 4.42 4.41 4.40 4.38 4.37 4.37 4.36 4.35
1.82 1.81 1.81 1.80 1.79 1.78 1.78 1.77 1.76 1.75 1.57 1.57 1.56 1.54 1.39 1.38 1.37 1.36 1.35 1.34 1.32 1.32 1.21 1.21 1.20 1.20 1.19 1.19 0.94 0.93
A (m) 4.39
B (m) 1.78
C (m) 1.57
D (m) 1.35
E (m) 1.20
F (s) 1.54
G (d) 0.93
2.00
2.90
1.85
6.09
3.11
1.03
3.11
f1 (ppm)

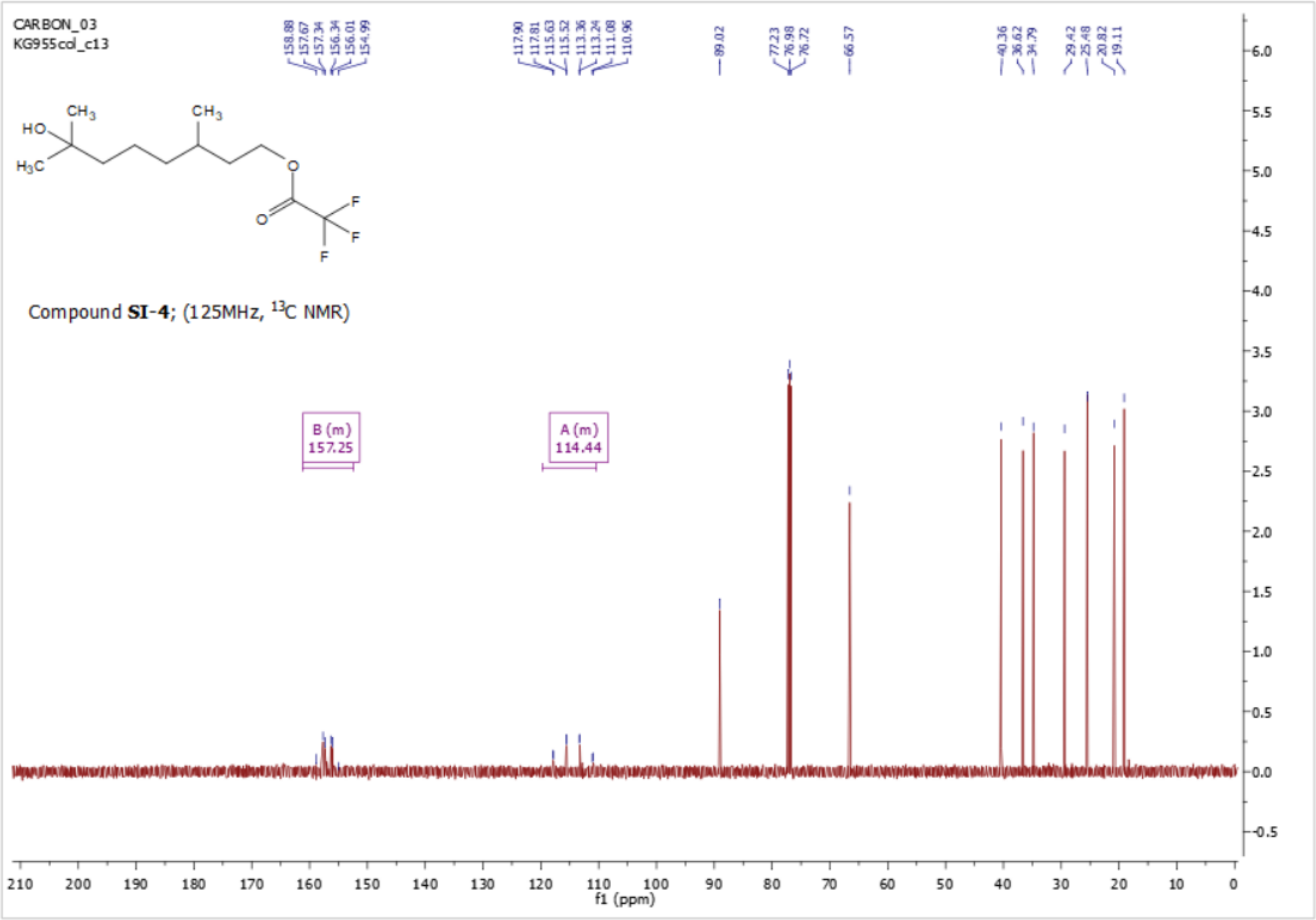
CARBON_03
KG955cdl_c13
Compound SI-4; (125MHz, 13C NMR)
158.88 157.67 157.34 156.34 156.01 154.99
117.90 117.81 115.63 115.52 113.36 113.24 111.08 110.96
89.02
77.23 76.98 76.72
66.57
40.36 36.62 34.79
29.42 25.48 20.82 19.11
B (m) 157.25
A (m) 114.44
f1 (ppm)

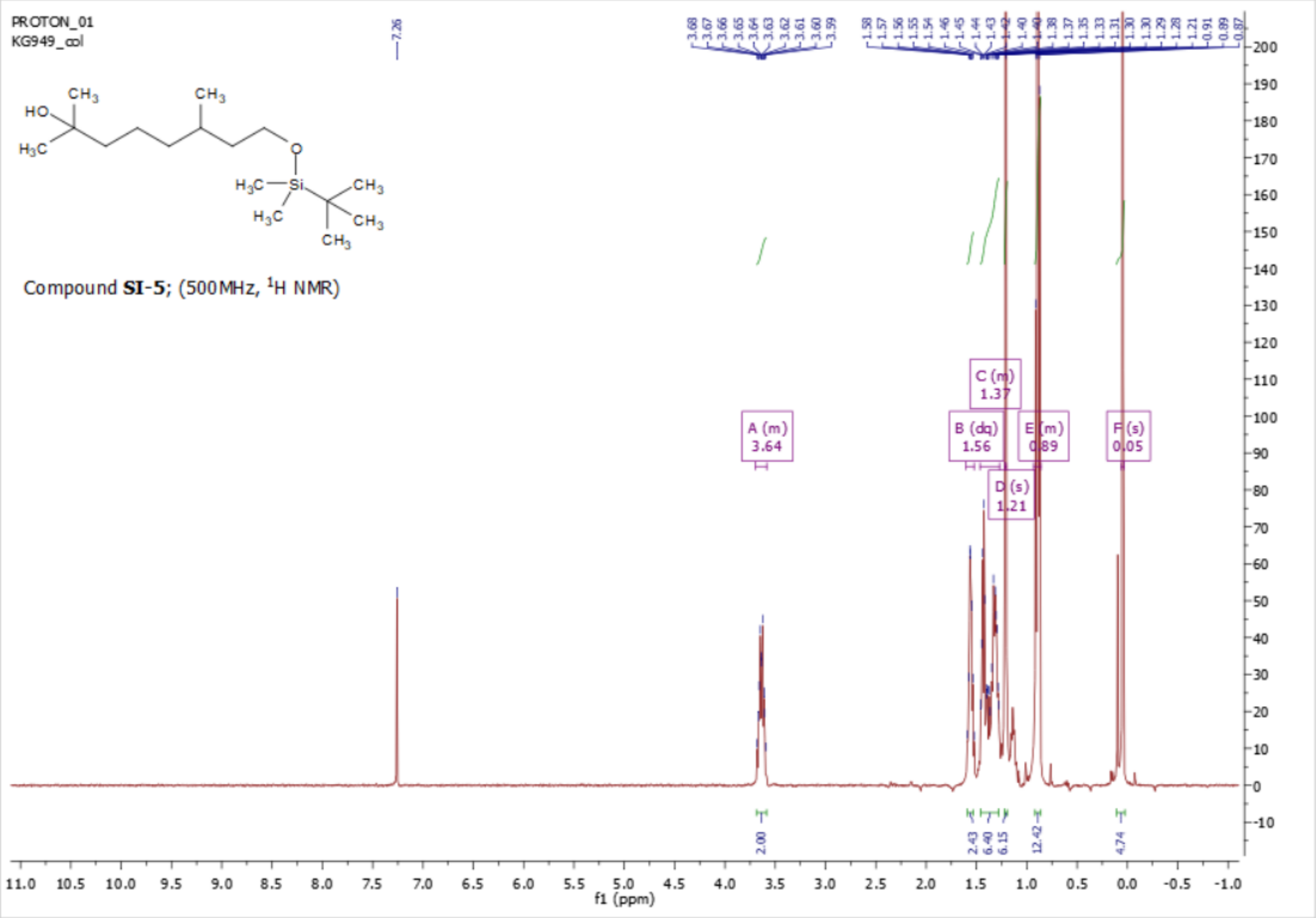
PROTON_01
KG949_cdl
Compound SI-5; (500MHz, 1H NMR)
f1 (ppm)

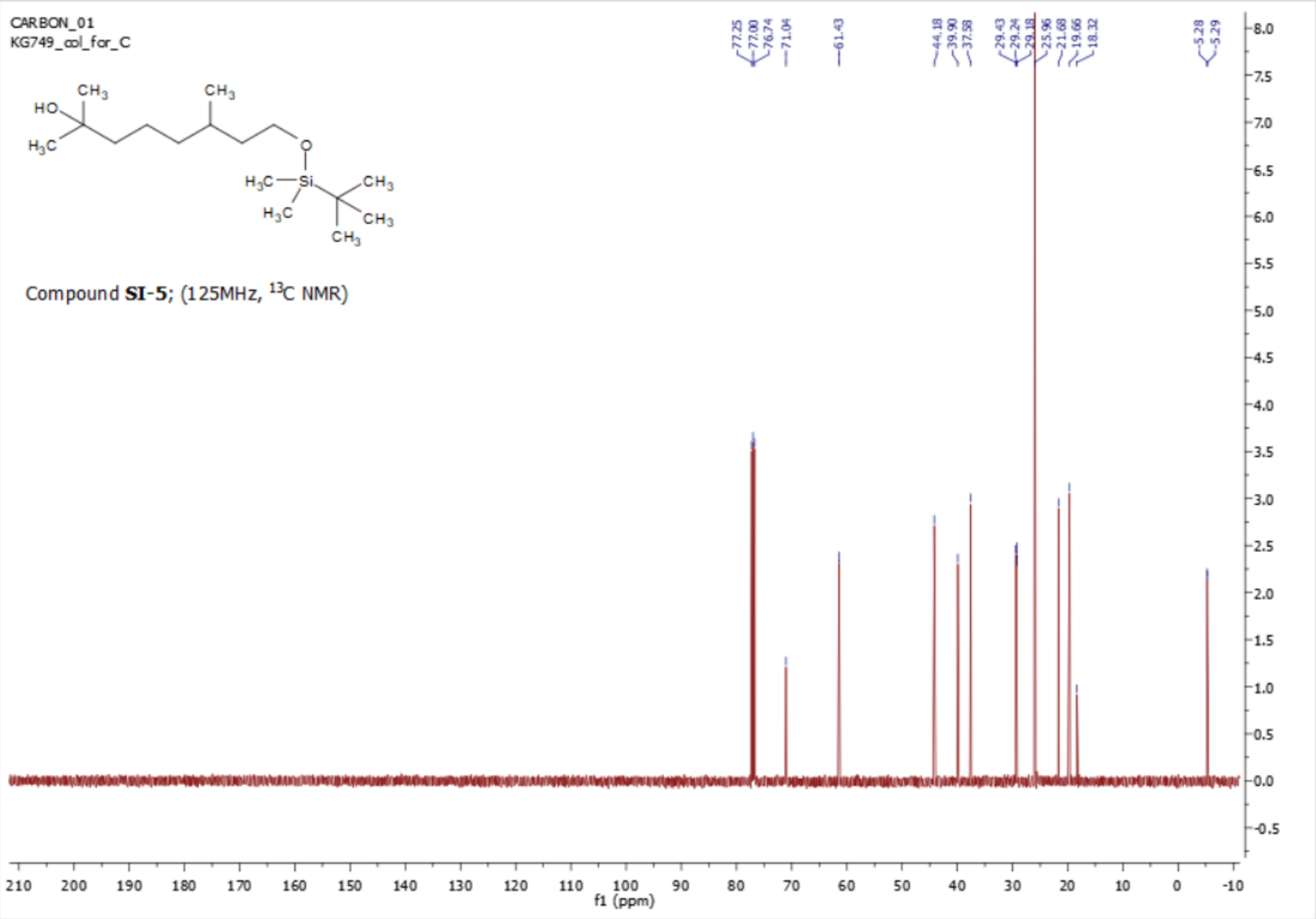
CARBON_01
KG749_cdl_for_C
Compound SI-5; (125MHz, 13C NMR)
f1 (ppm)

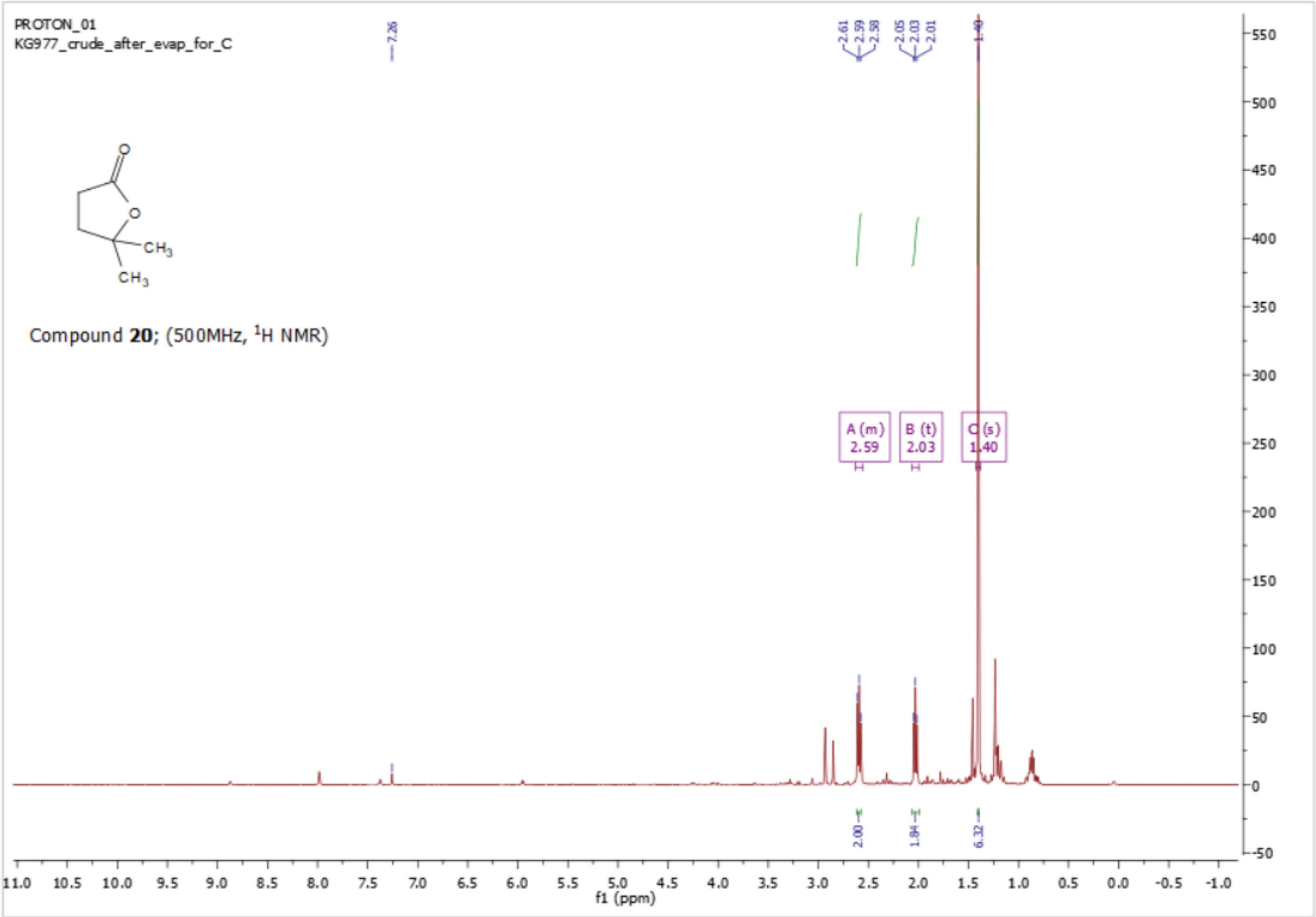
PROTON_01
KG977_crude_after_evap_for_C
Compound 20; (500MHz, 1H NMR)
A (m) 2.59
B (t) 2.03
C (s) 1.40
7.26
2.61 2.59 2.58
2.05 2.03 2.01
1.40
2.00
1.84
6.32
f1 (ppm)

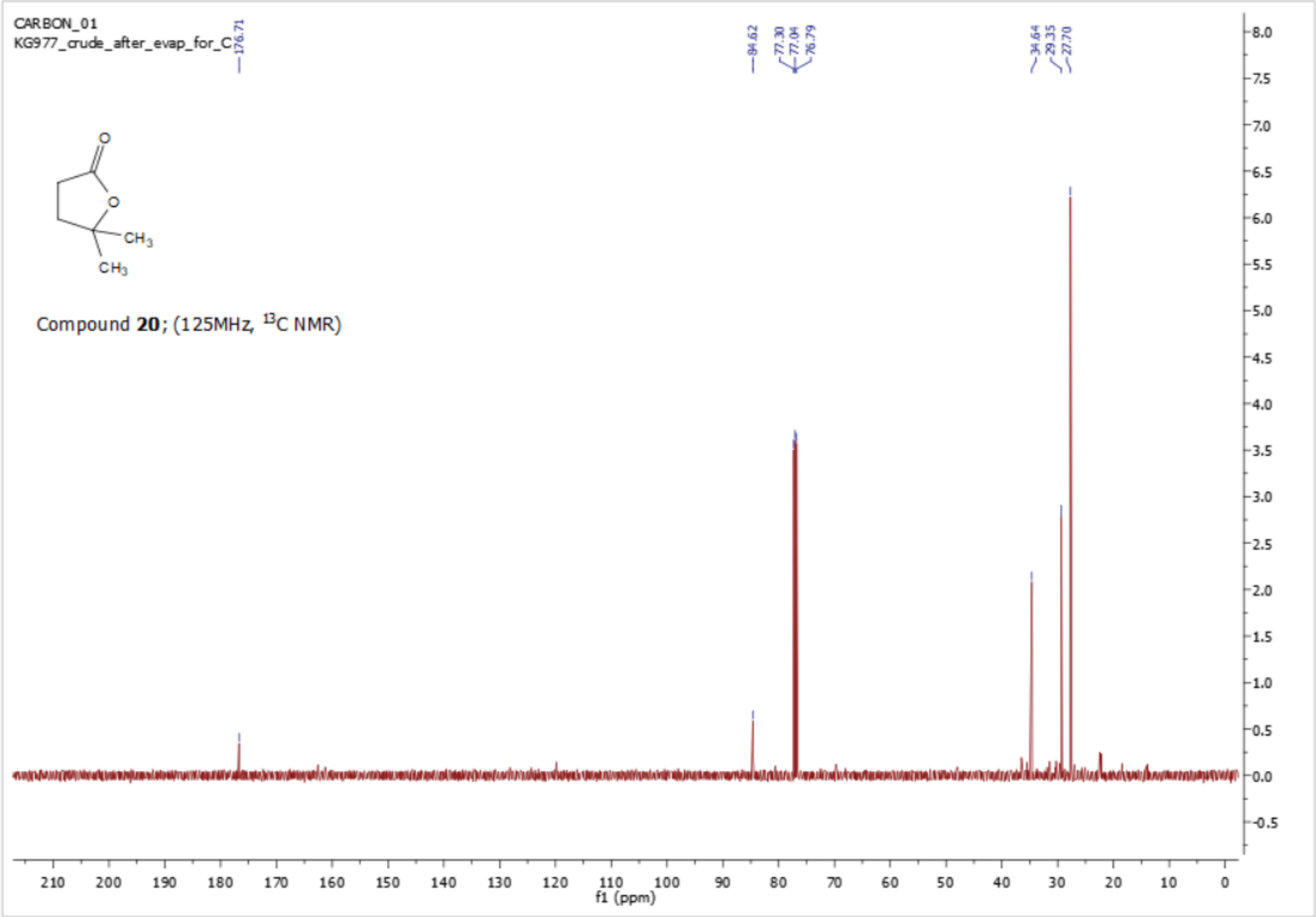
CARBON_01
KG977_crude_after_evap_for_C
Compound 20; (125MHz, 13C NMR)
176.71
84.62
77.30 77.04 76.79
34.64 29.35 27.70
f1 (ppm)

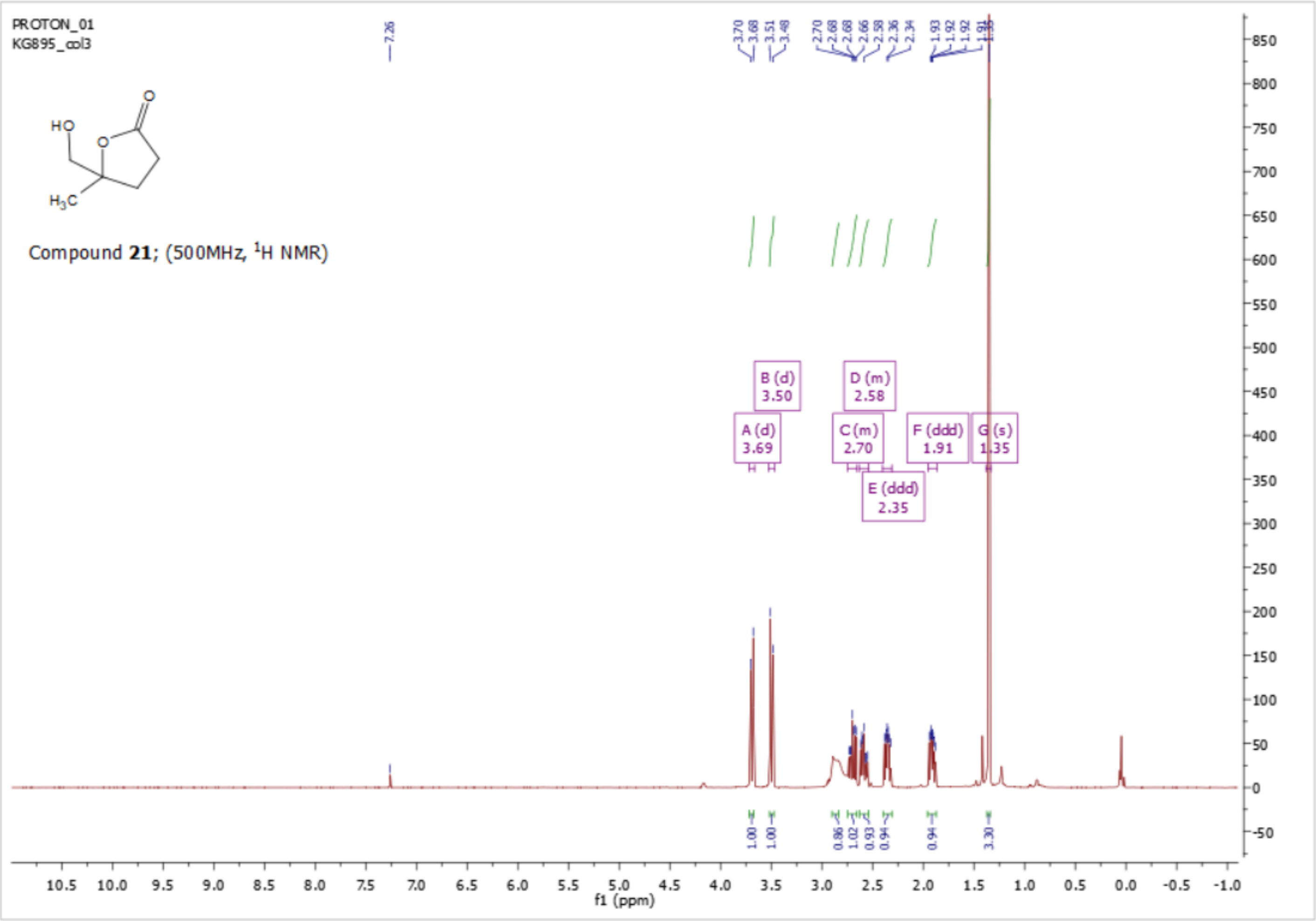

PROTON_01
KG895_cdcl3
Compound 21; (500MHz, 1H NMR)
A (d) 3.69
B (d) 3.50
C (m) 2.70
D (m) 2.58
E (ddd) 2.35
F (ddd) 1.91
G (s) 1.35
f1 (ppm)

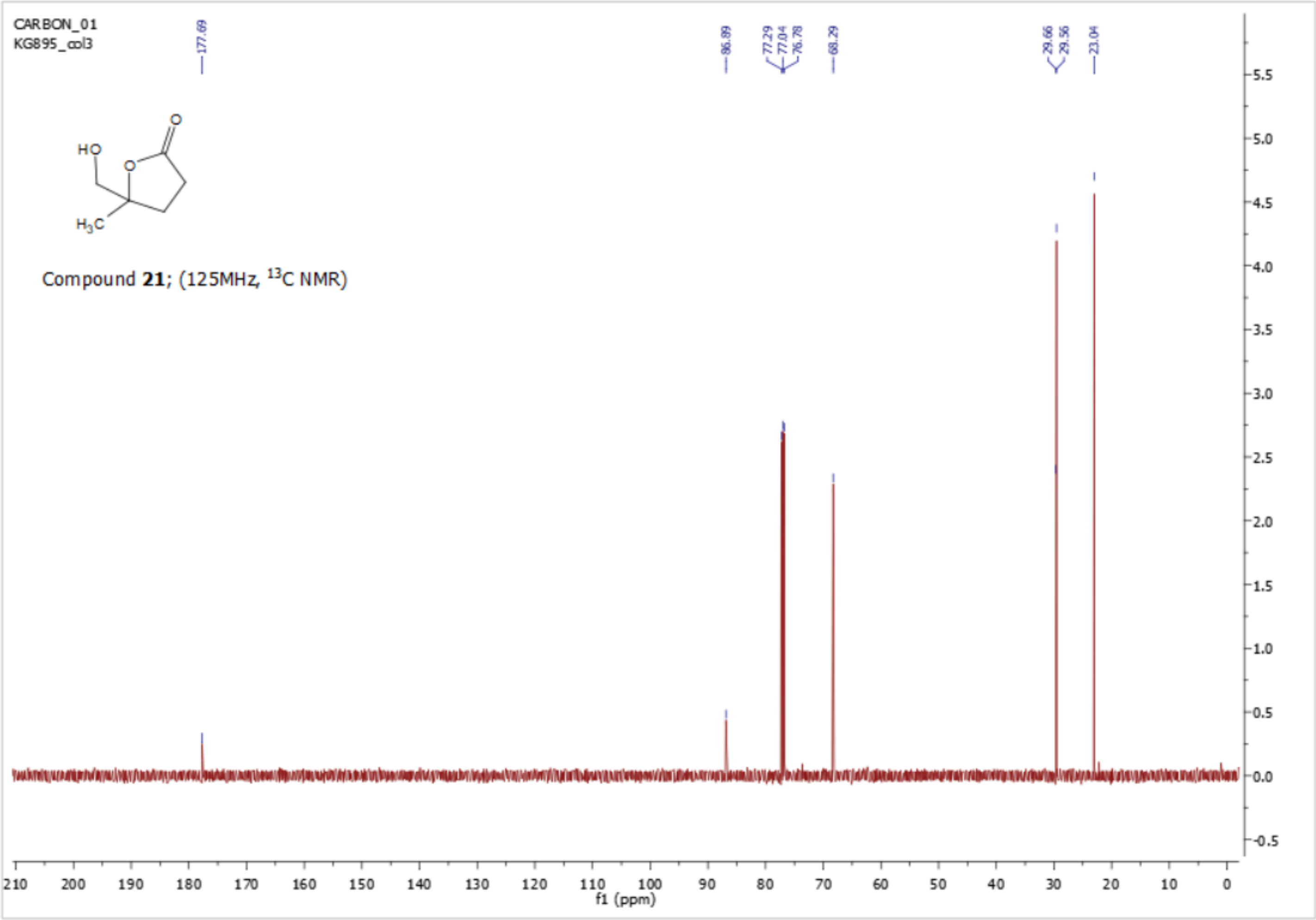

CARBON_01
KG895_cdcl3
Compound 21; (125MHz, 13C NMR)
177.69
86.89
77.29
77.04
76.78
68.29
29.66
29.56
23.04
f1 (ppm)

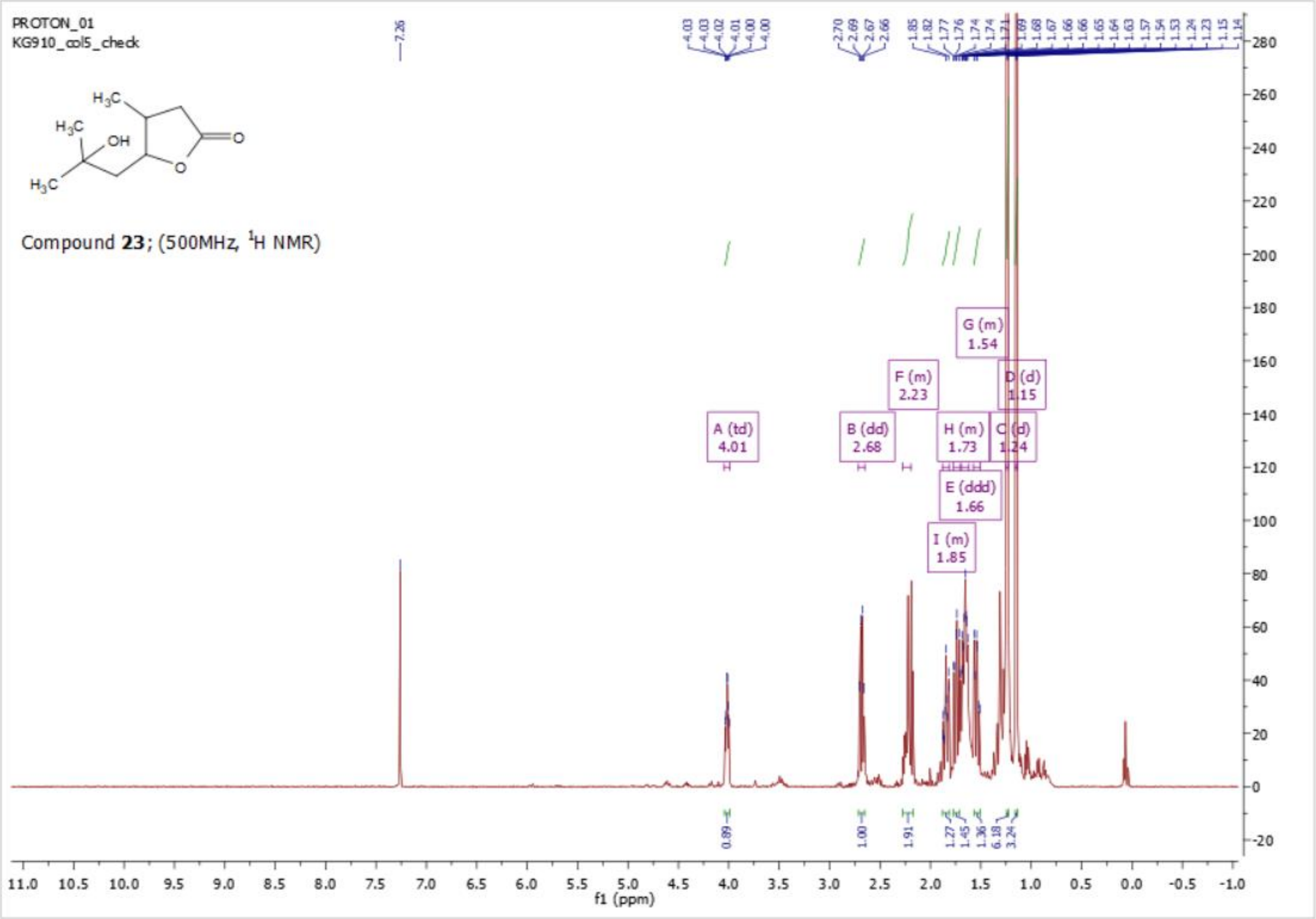
PROTON_01
KG910_col5_check
Compound 23; (500MHz, 1H NMR)
A (td) 4.01
B (dd) 2.68
F (m) 2.23
G (m) 1.54
H (m) 1.73
C (d) 1.24
D (d) 1.15
E (ddd) 1.66
I (m) 1.85
f1 (ppm)

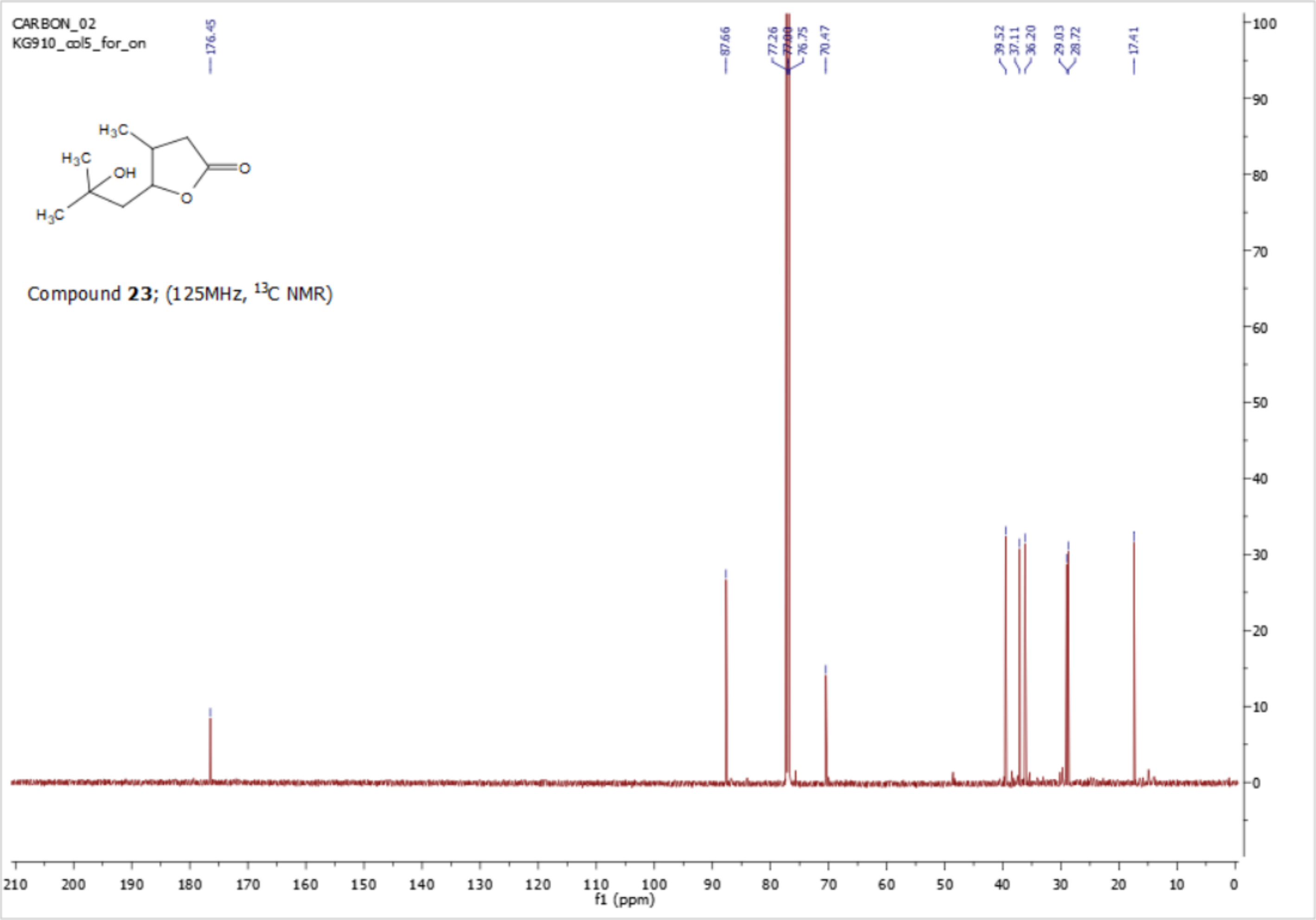
CARBON_02
KG910_col5_for_on
Compound 23; (125MHz, 13C NMR)
176.45
87.66
77.26
77.00
76.75
70.47
39.52
37.11
36.20
29.03
28.72
17.41
f1 (ppm)

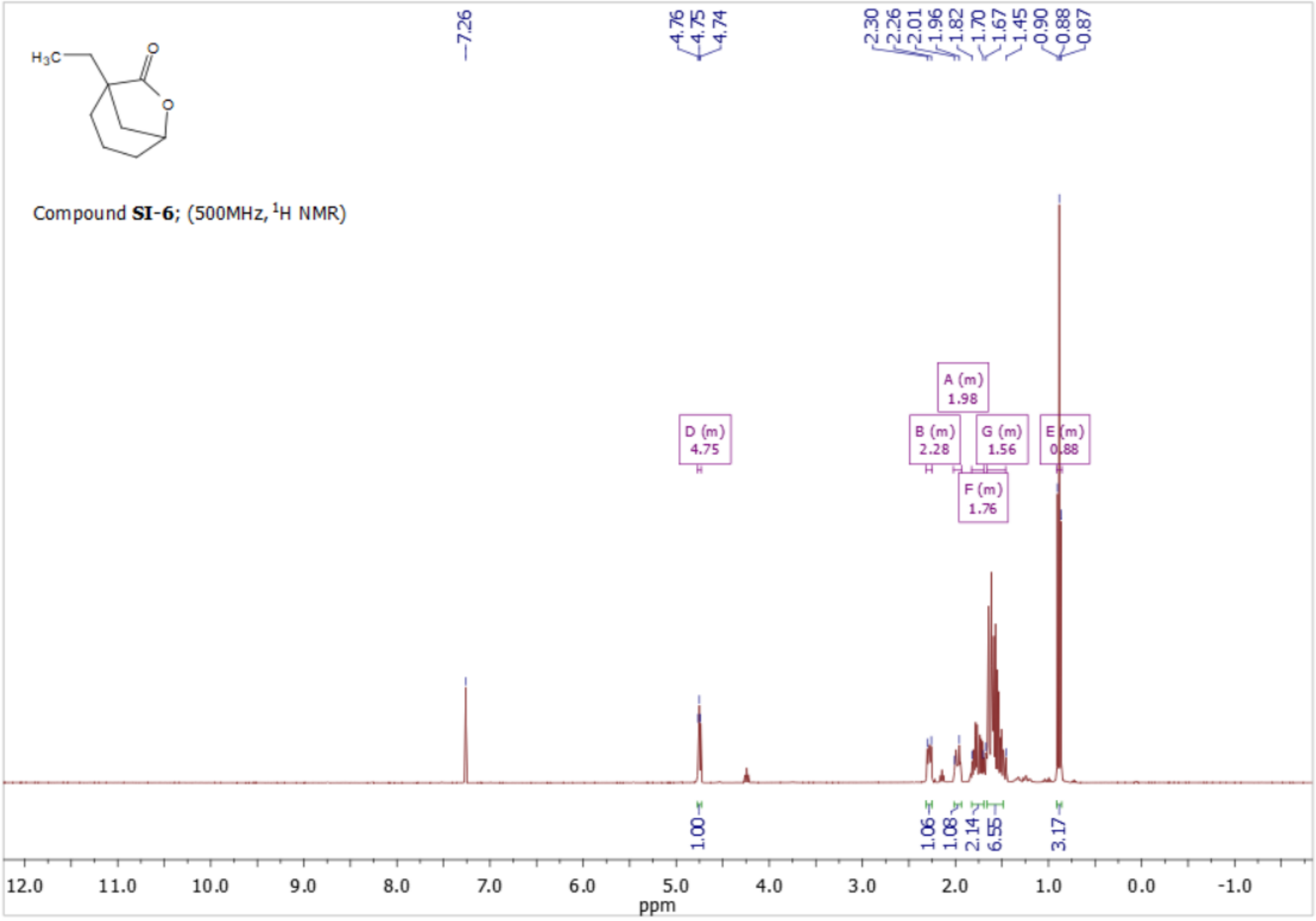
Compound SI-6; (500MHz, 1H NMR)
7.26
4.76 4.75 4.74
2.30 2.26 2.01 1.96 1.82 1.70 1.67 1.45 0.90 0.88 0.87
A (m) 1.98
B (m) 2.28
D (m) 4.75
E (m) 0.88
F (m) 1.76
G (m) 1.56
1.00
1.06
1.08
2.14
6.55
3.17
ppm

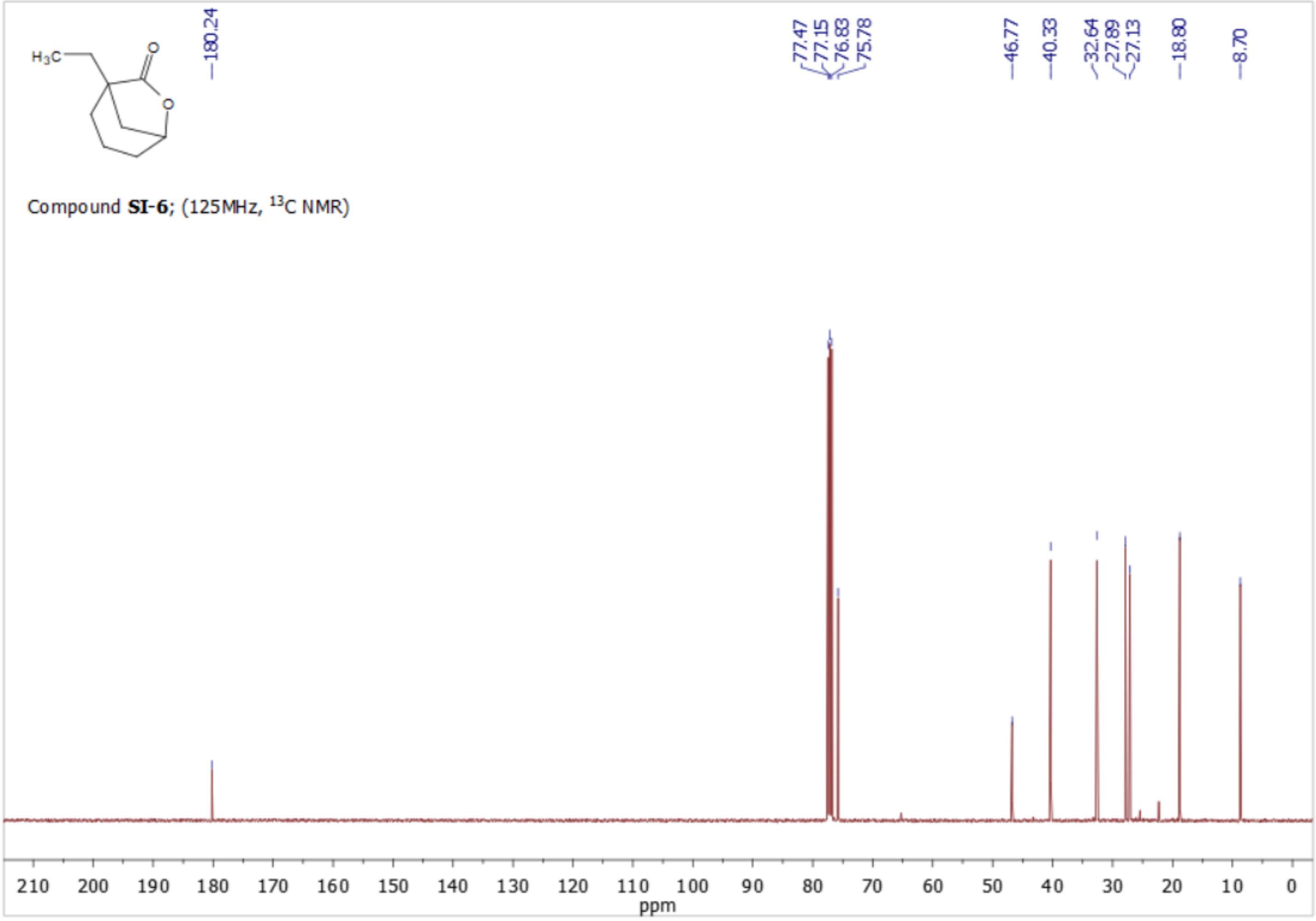
Compound SI-6; (125MHz, 13C NMR)
180.24
77.47 77.15 76.83 75.78
46.77
40.33
32.64 27.89 27.13
18.80
8.70
ppm

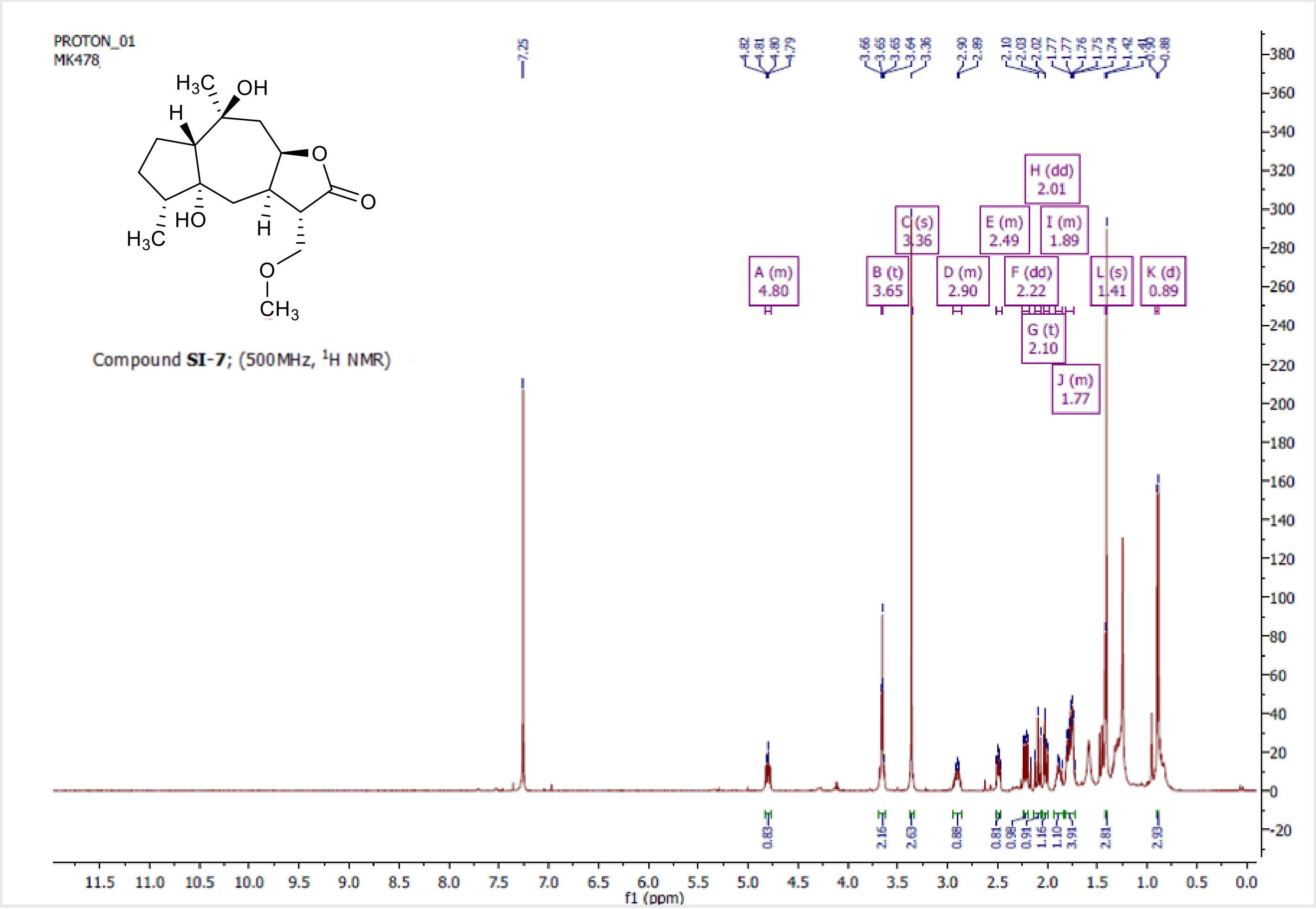

Compound **SI-7**; (500MHz, ^{1}H NMR)

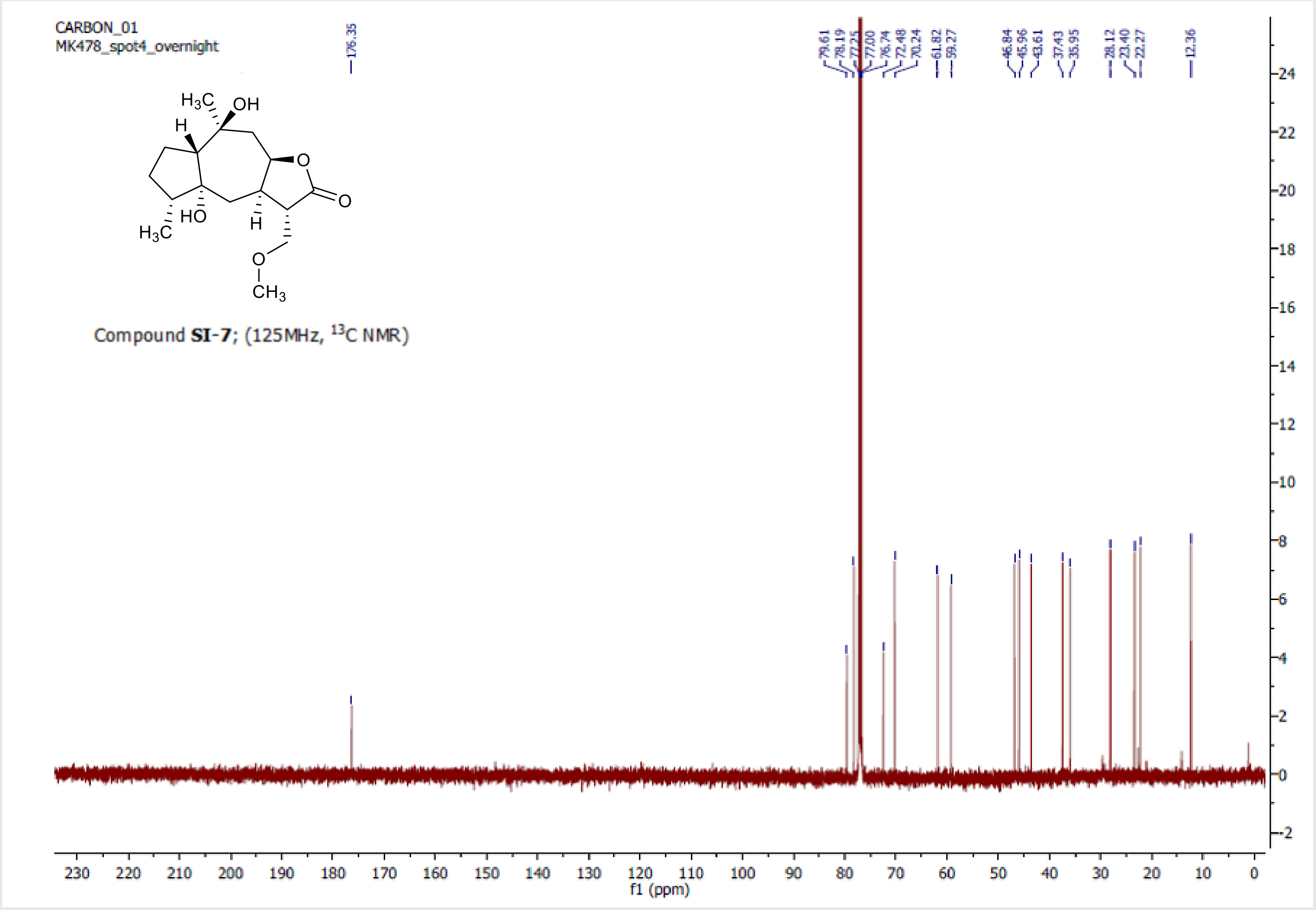

Compound **SI-7**; (125MHz, ^{13}C NMR)

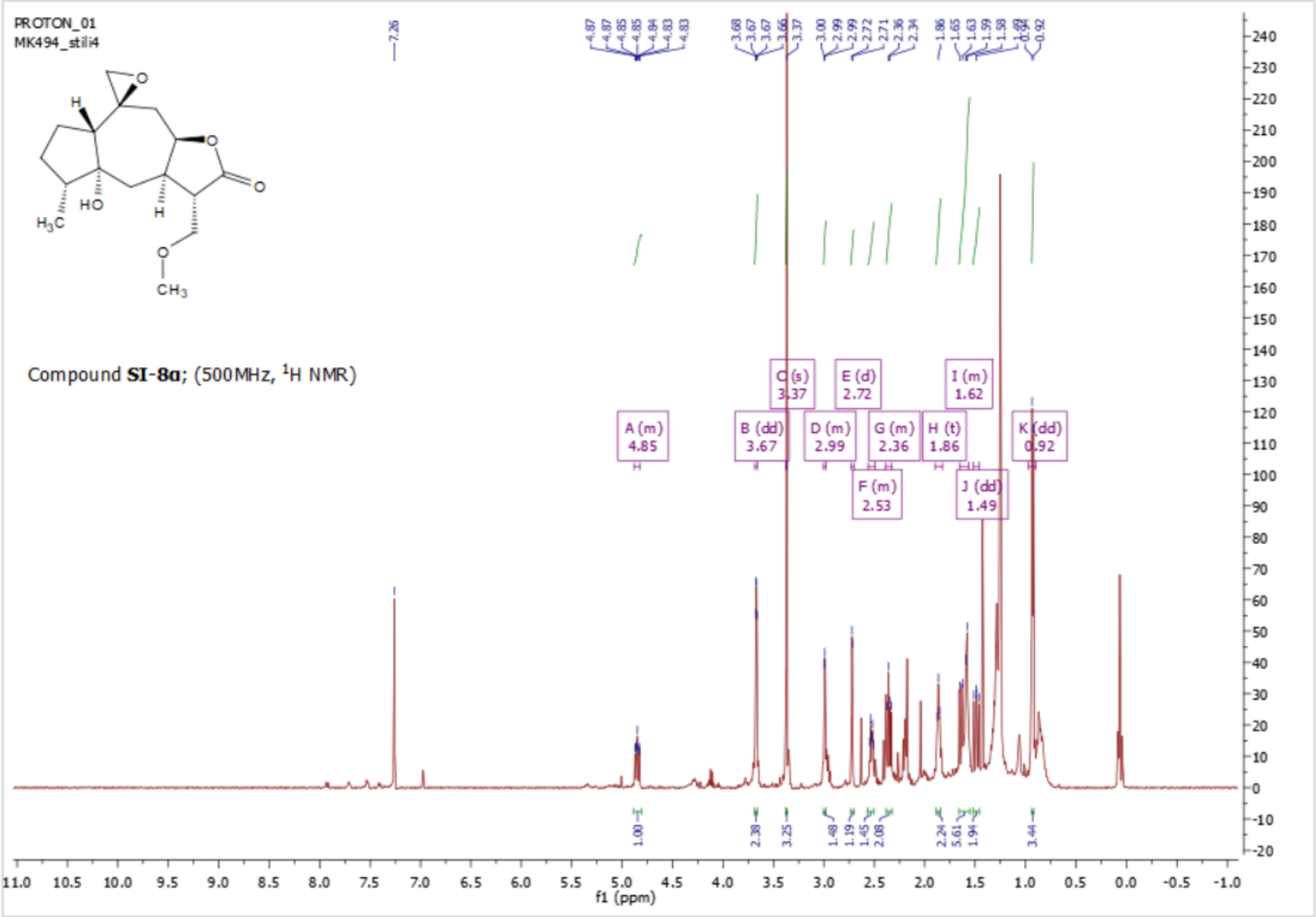
PROTON_01
MK494_stili4
Compound SI-8α; (500MHz, 1H NMR)
A (m) 4.85
B (dd) 3.67
C (s) 3.37
D (m) 2.99
E (d) 2.72
F (m) 2.53
G (m) 2.36
H (t) 1.86
I (m) 1.62
J (dd) 1.49
K (dd) 0.92
f1 (ppm)

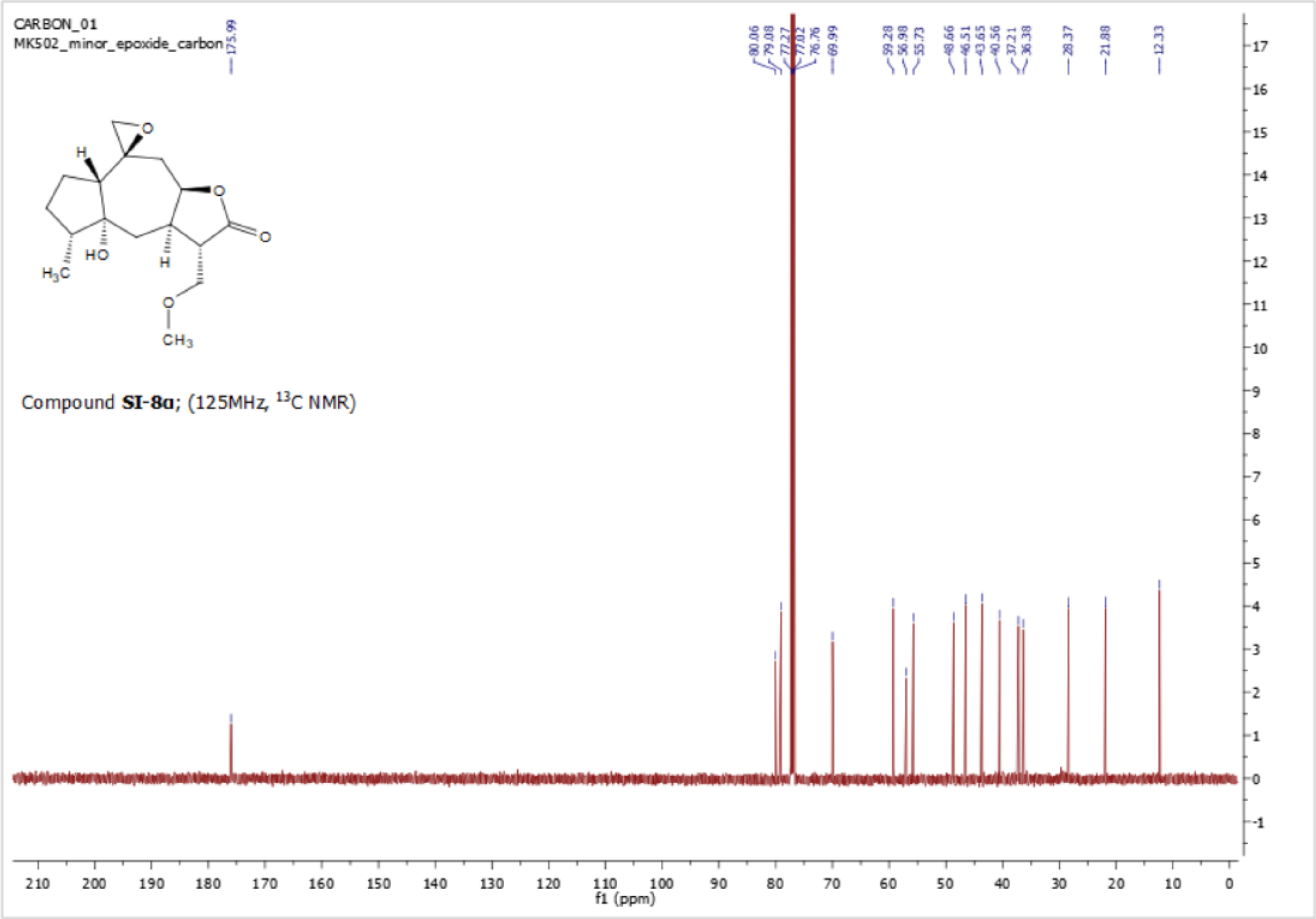
CARBON_01
MK502_minor_epoxide_carbon
Compound SI-8α; (125MHz, 13C NMR)
175.99
80.06
79.08
77.27
77.02
76.76
69.99
59.28
56.98
55.73
48.66
46.51
43.65
40.56
37.21
36.38
28.37
21.88
12.33
f1 (ppm)

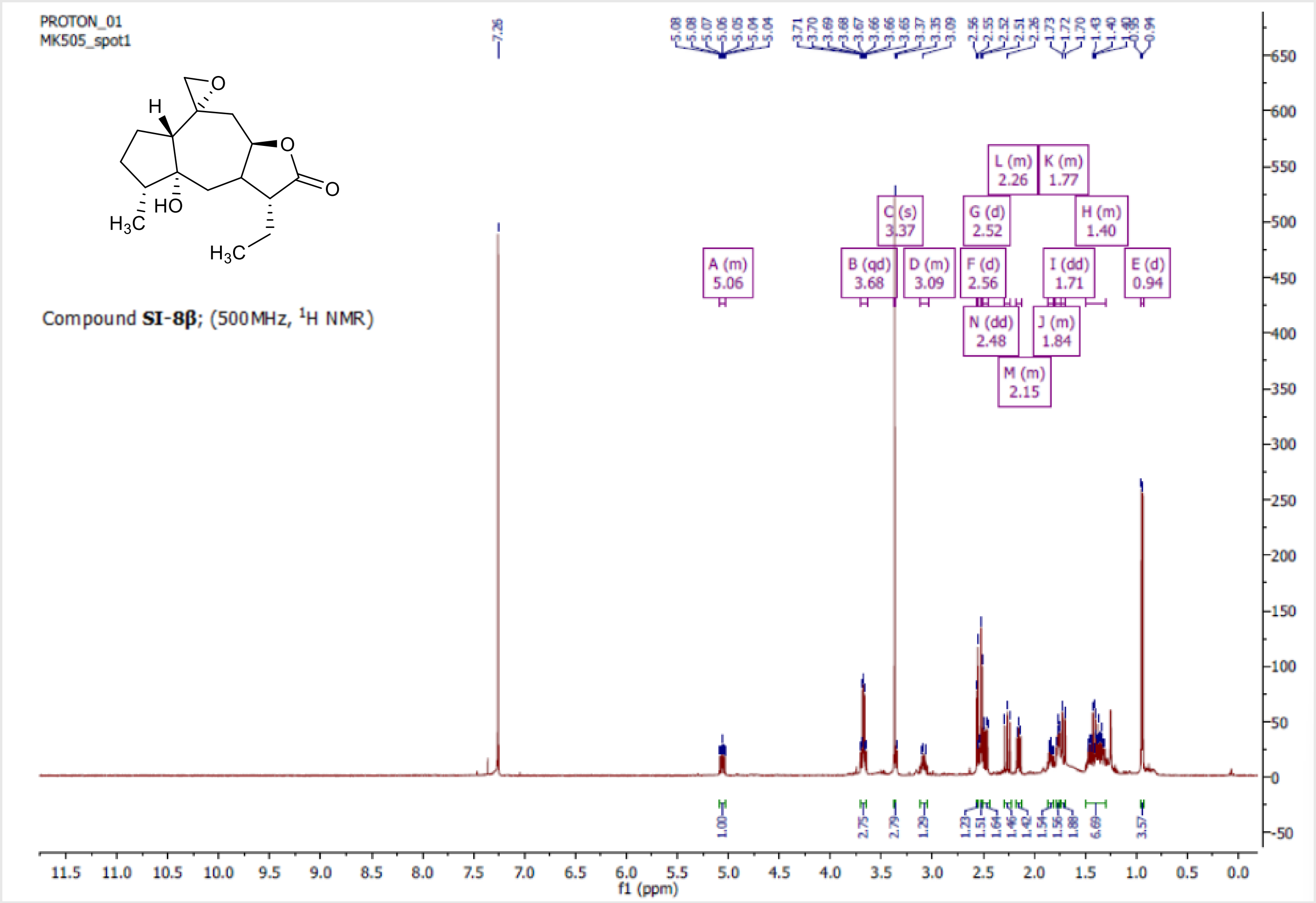
PROTON_01
MK505_spot1
Compound SI-8β; (500MHz, 1H NMR)
A (m) 5.06
B (qd) 3.68
C (s) 3.37
D (m) 3.09
E (d) 0.94
F (d) 2.56
G (d) 2.52
H (m) 1.40
I (dd) 1.71
J (m) 1.84
K (m) 1.77
L (m) 2.26
M (m) 2.15
N (dd) 2.48
f1 (ppm)

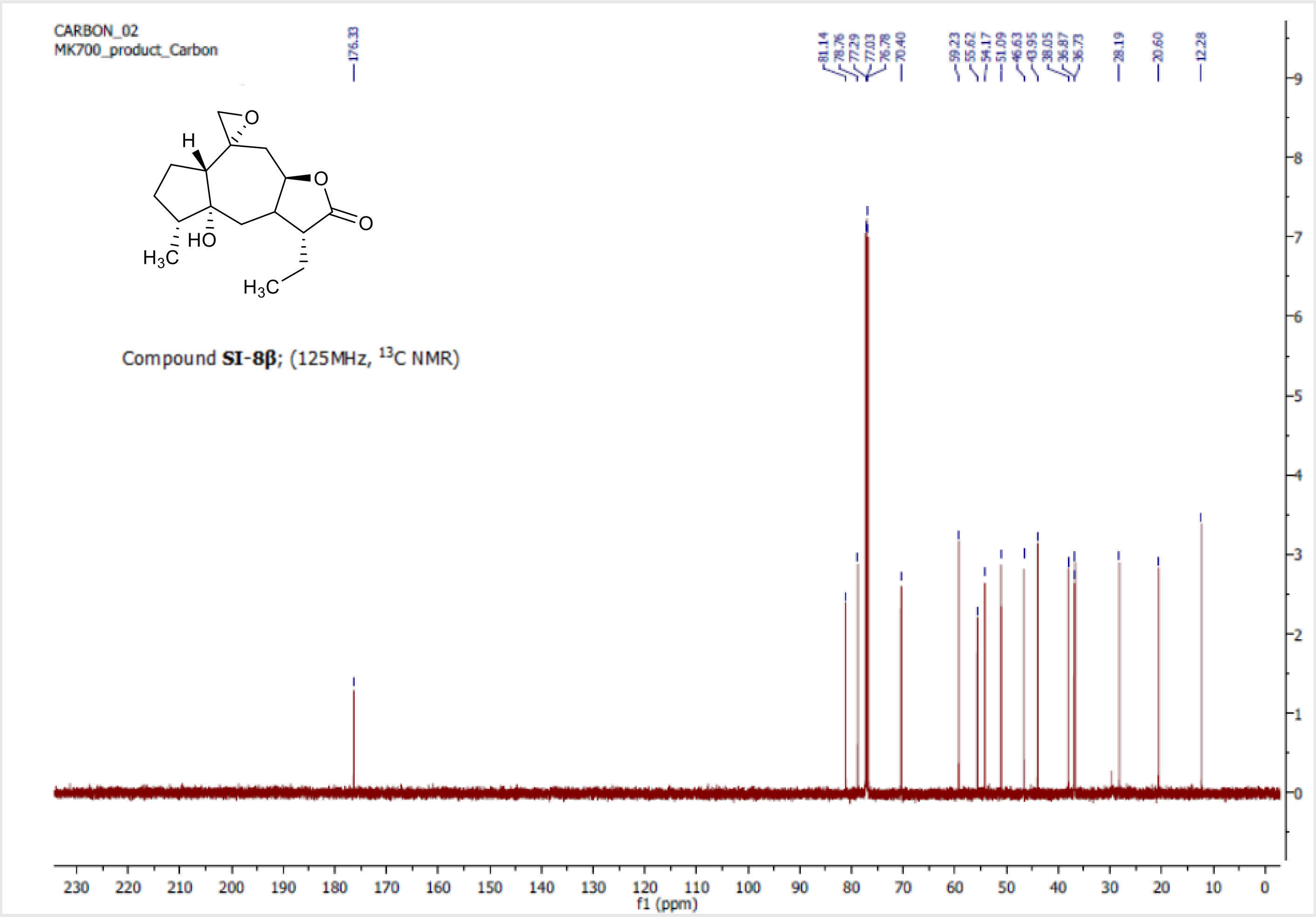
CARBON_02
MK700_product_Carbon
176.33
81.14
78.76
77.29
77.03
76.78
70.40
59.23
55.62
54.17
51.09
46.63
43.95
38.05
36.87
36.73
28.19
20.60
12.28
Compound SI-8β; (125MHz, 13C NMR)
f1 (ppm)

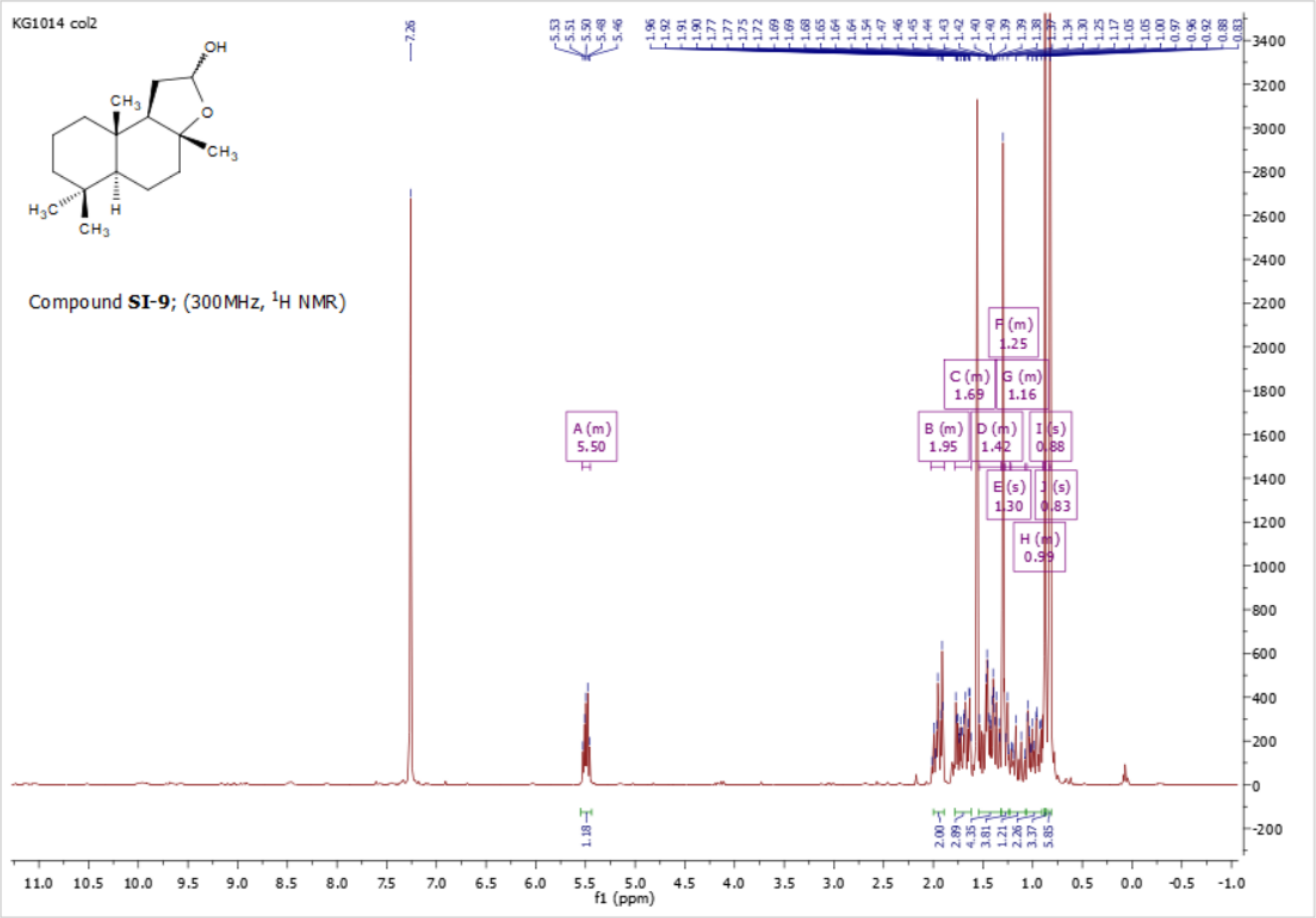
KG1014 col2
Compound **SI-9**; (300MHz, ^{1}H NMR)
f1 (ppm)

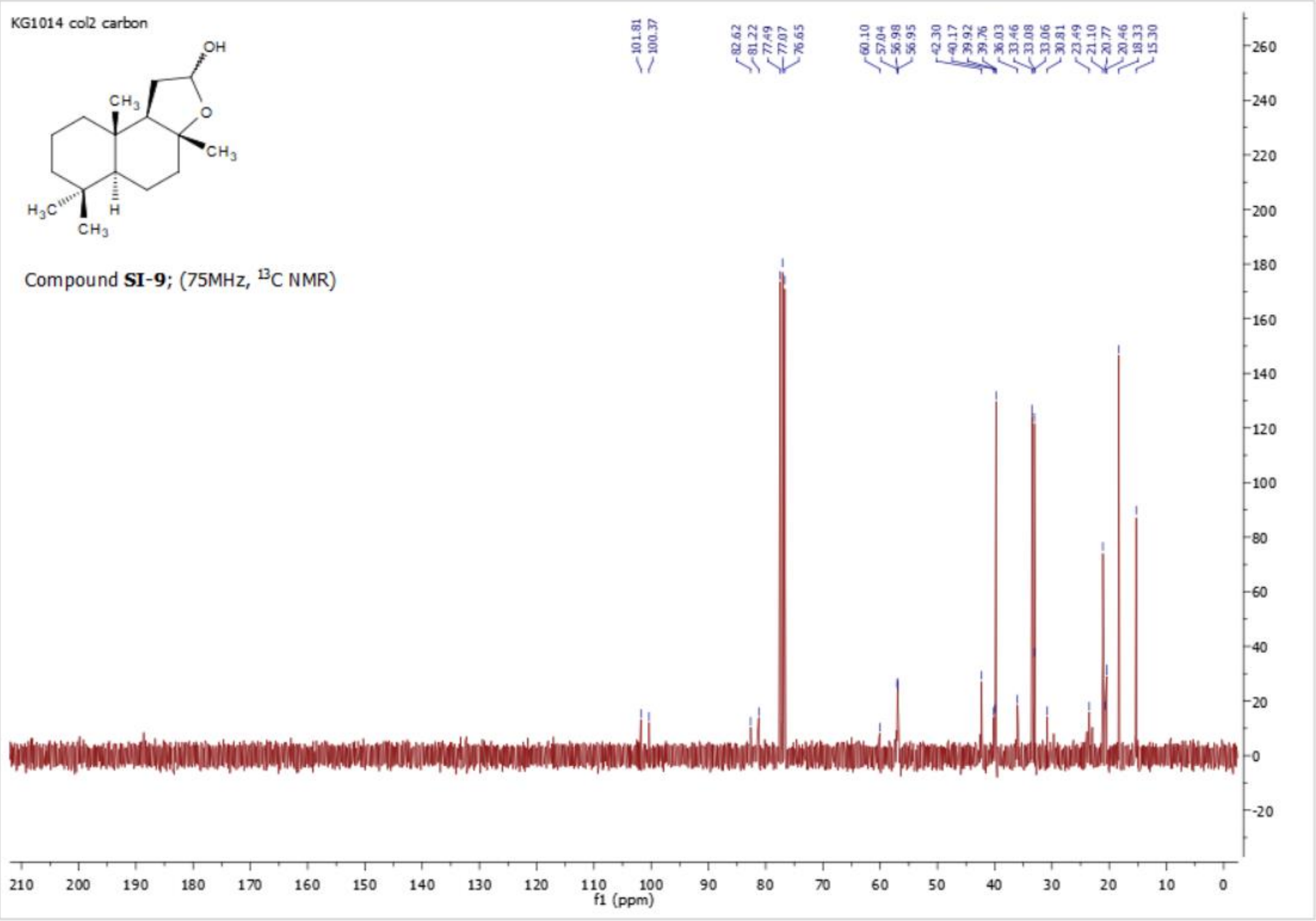
KG1014 col2 carbon
Compound **SI-9**; (75MHz, ^{13}C NMR)
f1 (ppm)

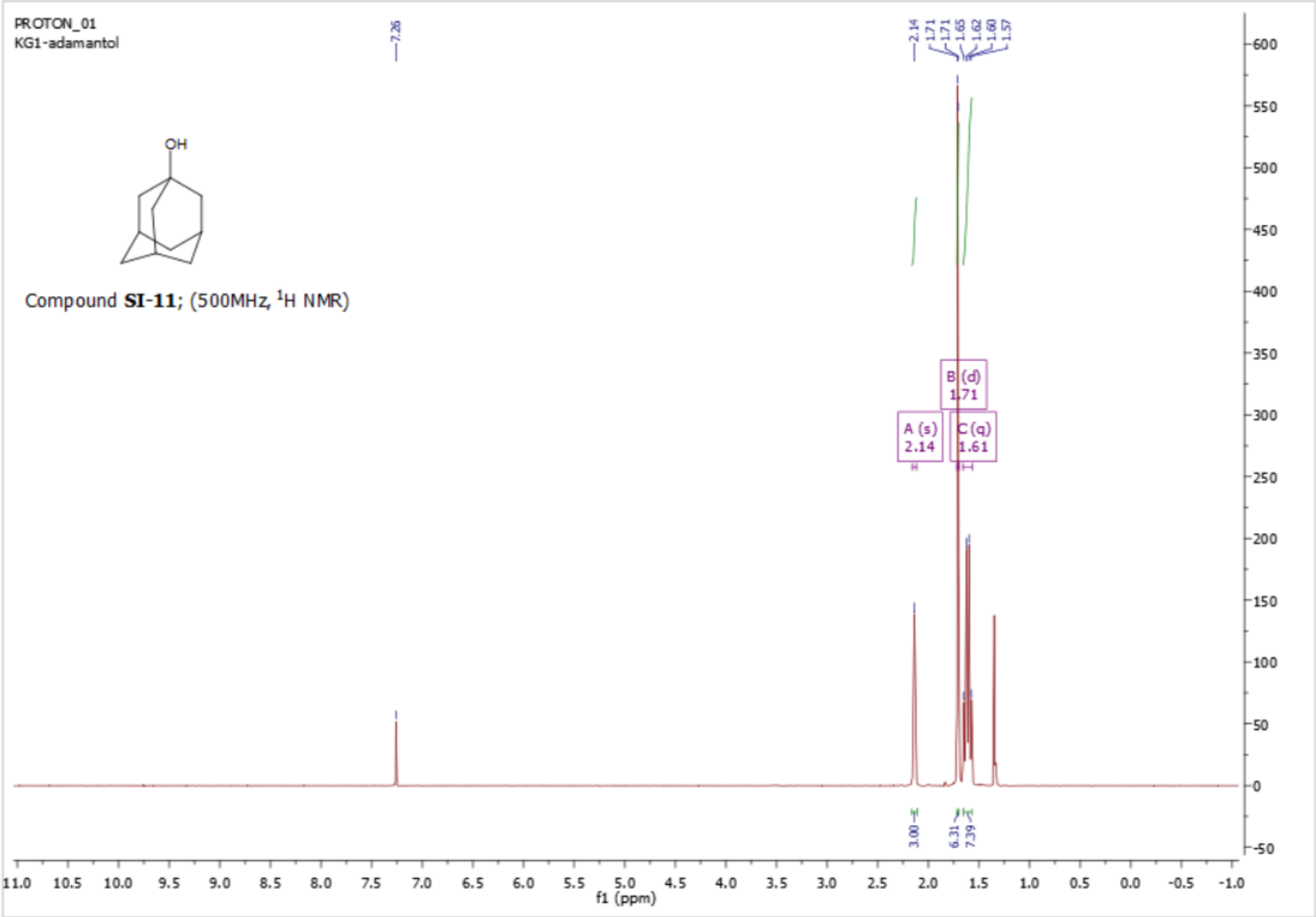
PROTON_01
KG1-adamantol
OH
Compound SI-11; (500MHz, 1H NMR)
7.26
2.14
1.71
1.71
1.65
1.62
1.60
1.57
B (d)
1.71
A (s)
2.14
C (q)
1.61
3.00
6.31
7.39
f1 (ppm)

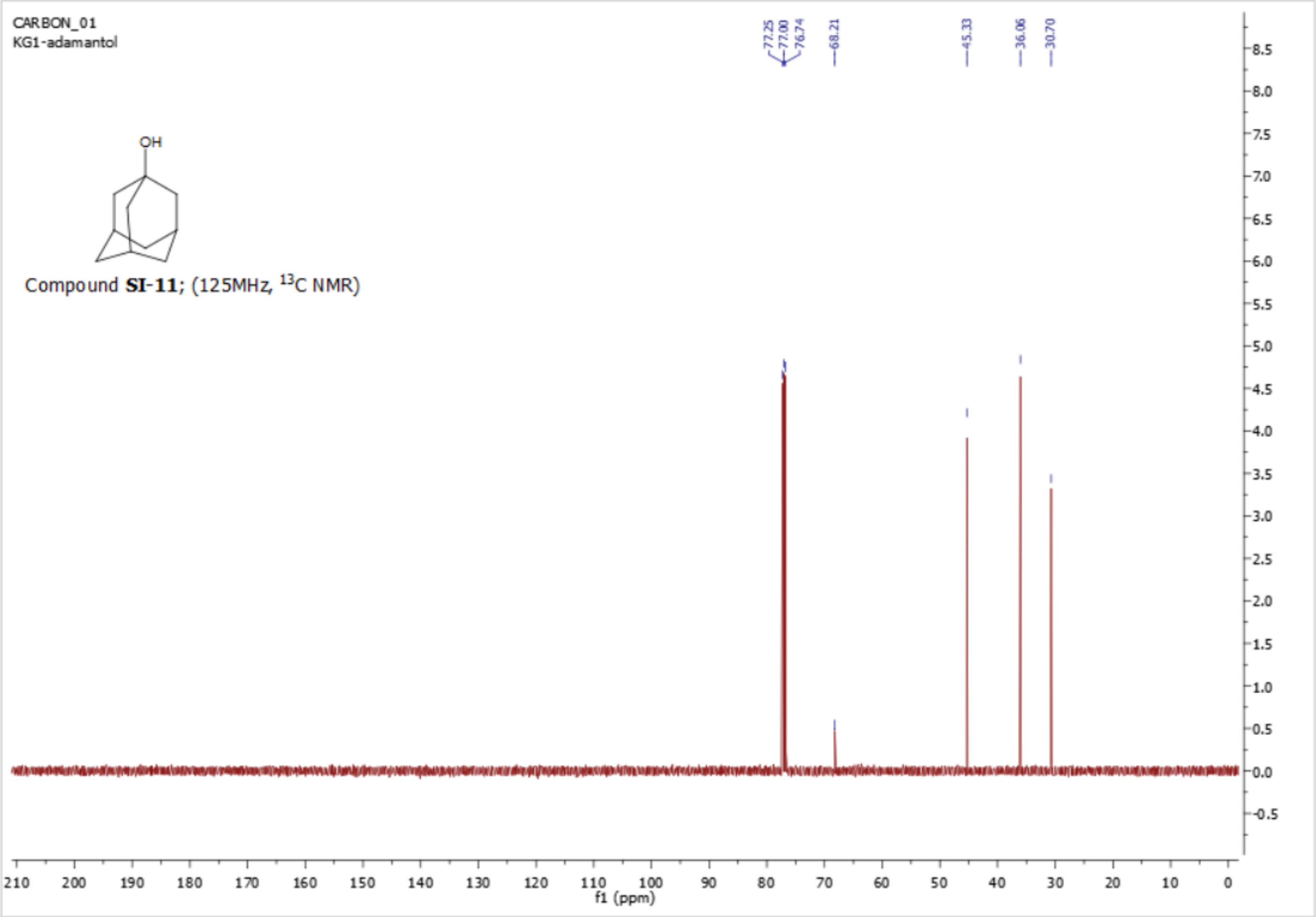
CARBON_01
KG1-adamantol
OH
Compound SI-11; (125MHz, 13C NMR)
77.25
77.00
76.74
68.21
45.33
36.06
30.70
f1 (ppm)

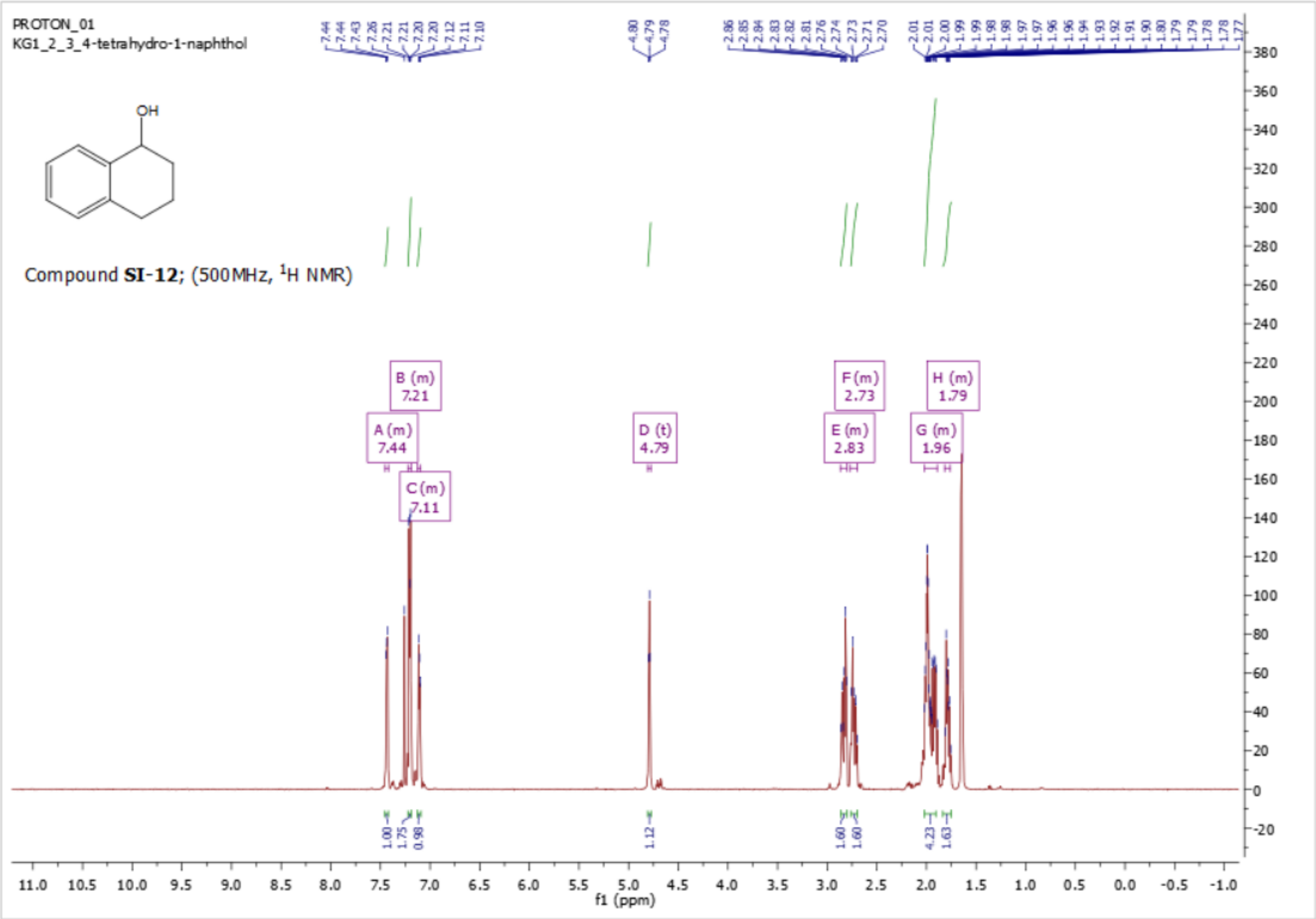
PROTON_01
KG1_2_3_4-tetrahydro-1-naphthol
OH
Compound SI-12; (500MHz, 1H NMR)
A (m) 7.44
B (m) 7.21
C (m) 7.11
D (t) 4.79
E (m) 2.83
F (m) 2.73
G (m) 1.96
H (m) 1.79
f1 (ppm)

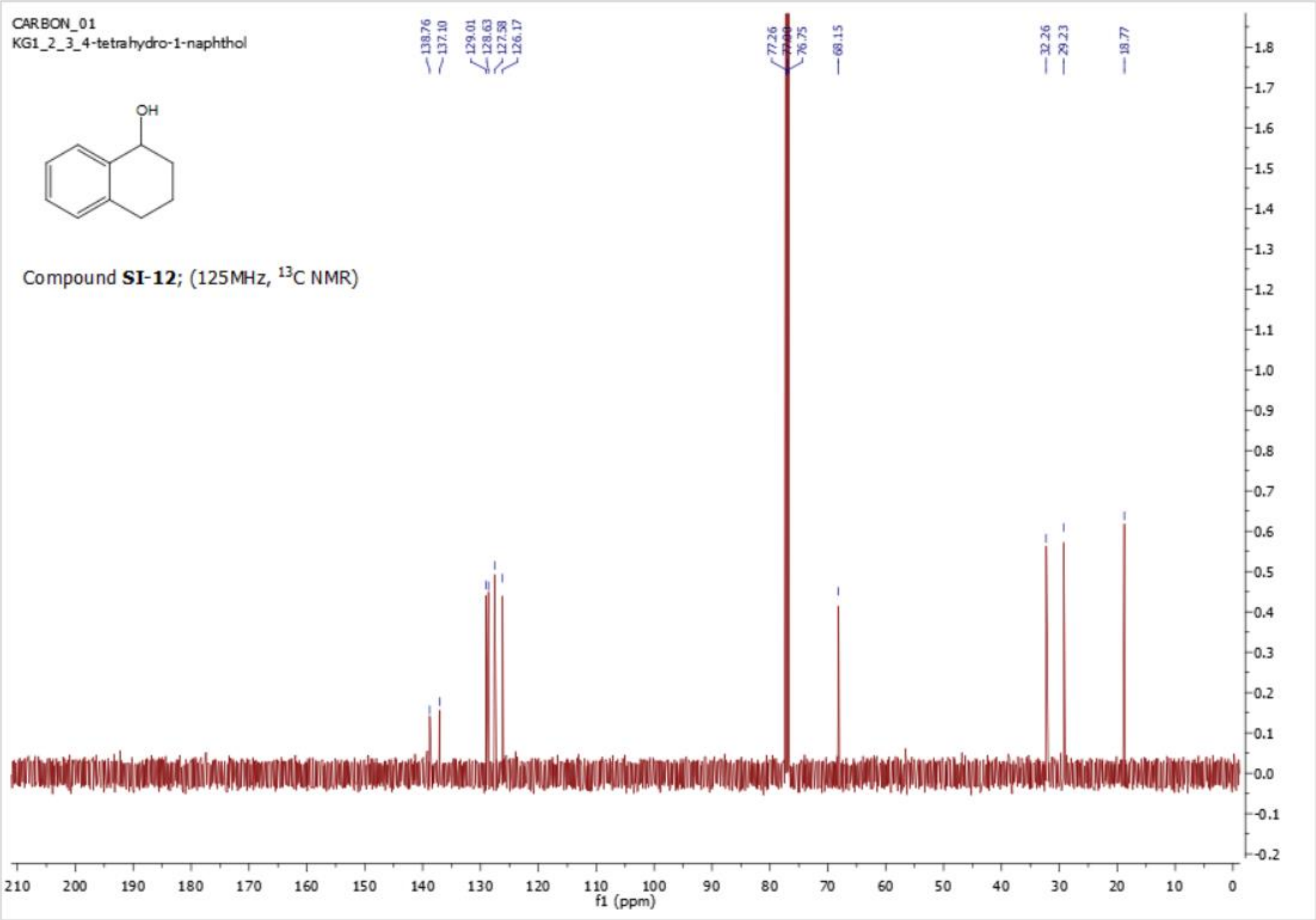
CARBON_01
KG1_2_3_4-tetrahydro-1-naphthol
OH
Compound SI-12; (125MHz, 13C NMR)
138.76
137.10
129.01
128.63
127.58
126.17
77.26
76.75
68.15
32.26
29.23
18.77
f1 (ppm)

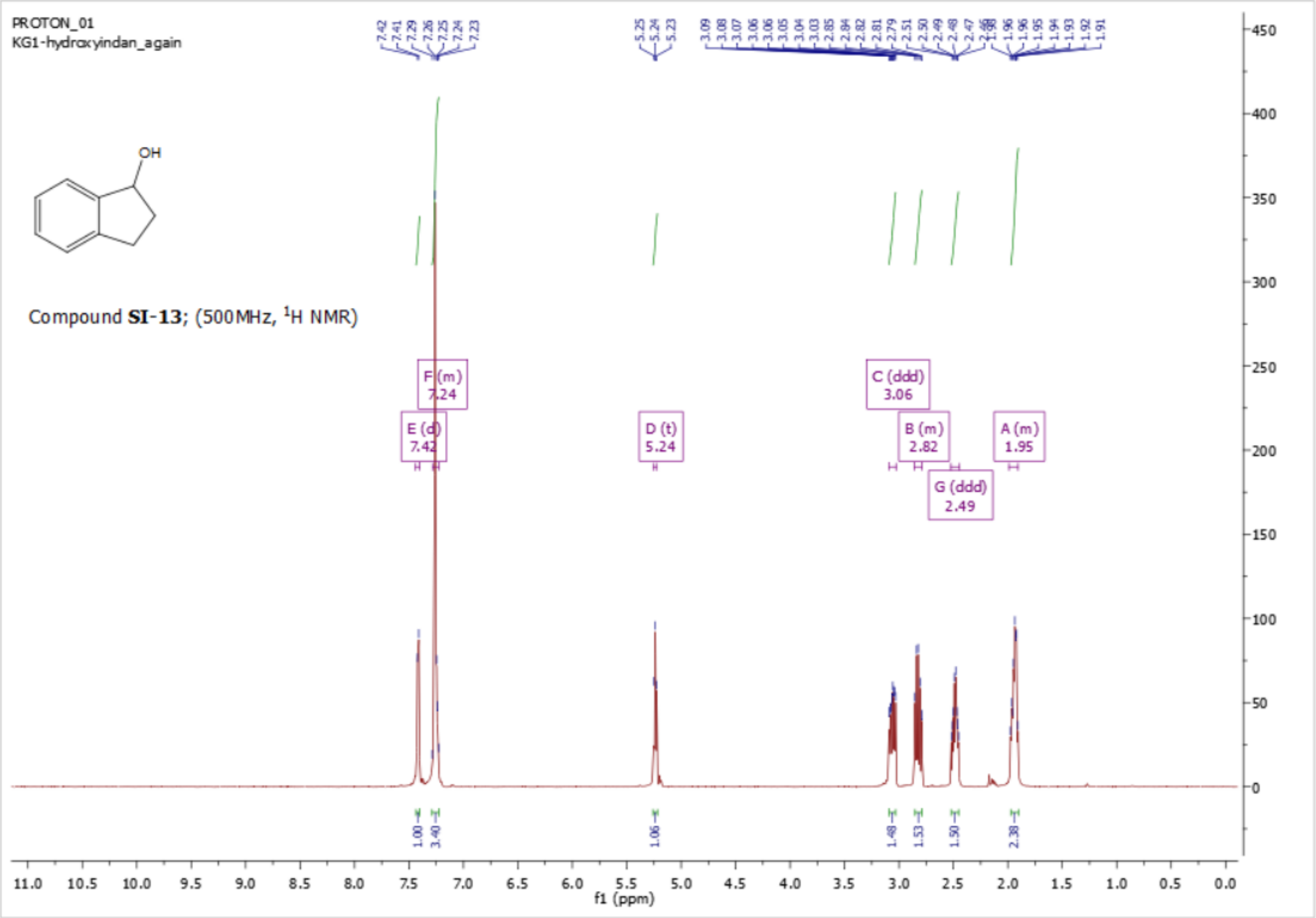
PROTON_01
KG1-hydroxyindan_again
OH
Compound SI-13; (500MHz, 1H NMR)
F (m) 7.24
E (d) 7.42
D (t) 5.24
C (ddd) 3.06
B (m) 2.82
G (ddd) 2.49
A (m) 1.95
f1 (ppm)

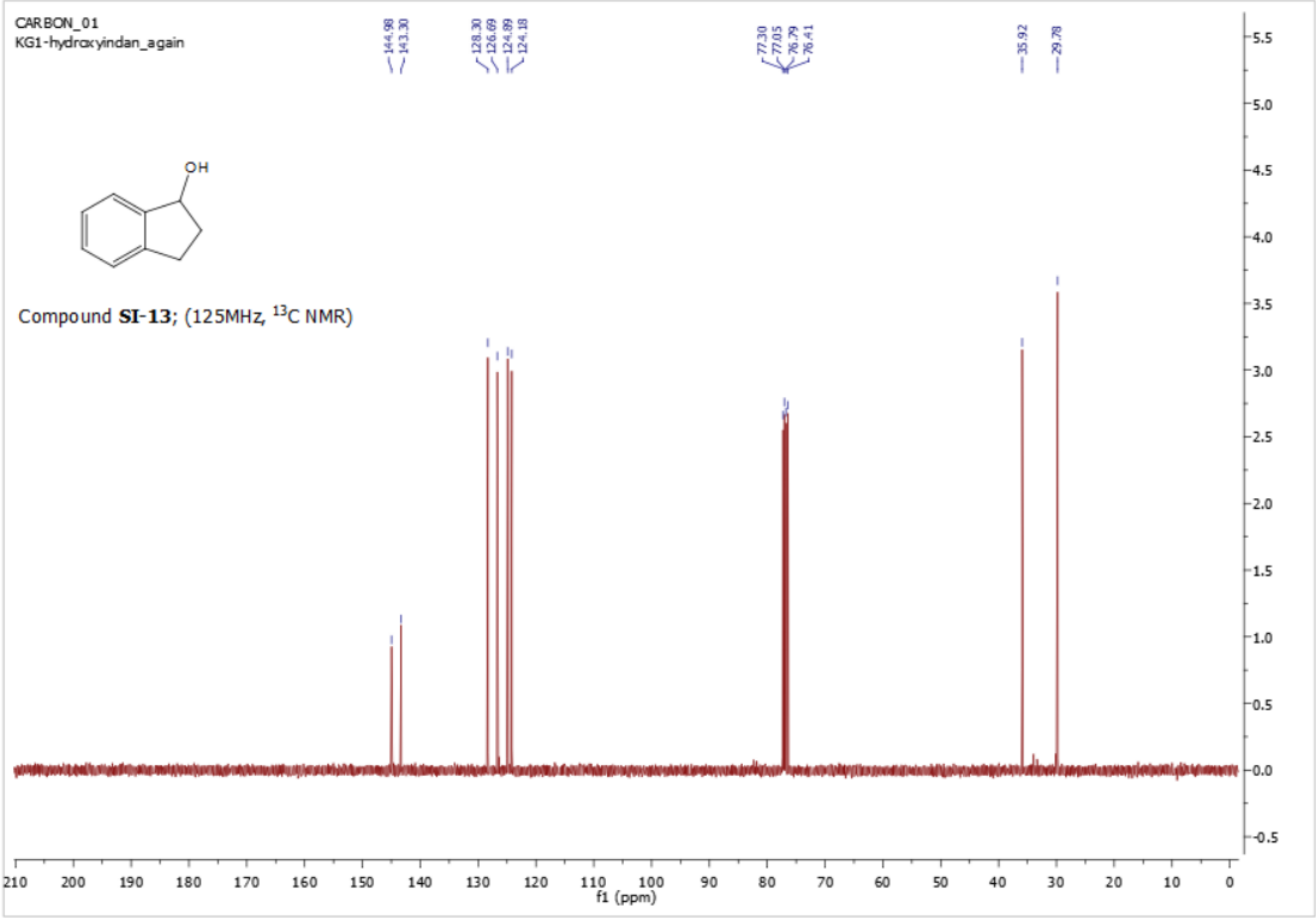
CARBON_01
KG1-hydroxyindan_again
144.98
143.30
128.30
126.69
124.89
124.18
77.30
77.05
76.79
76.41
35.92
29.78
OH
Compound SI-13; (125MHz, 13C NMR)
f1 (ppm)

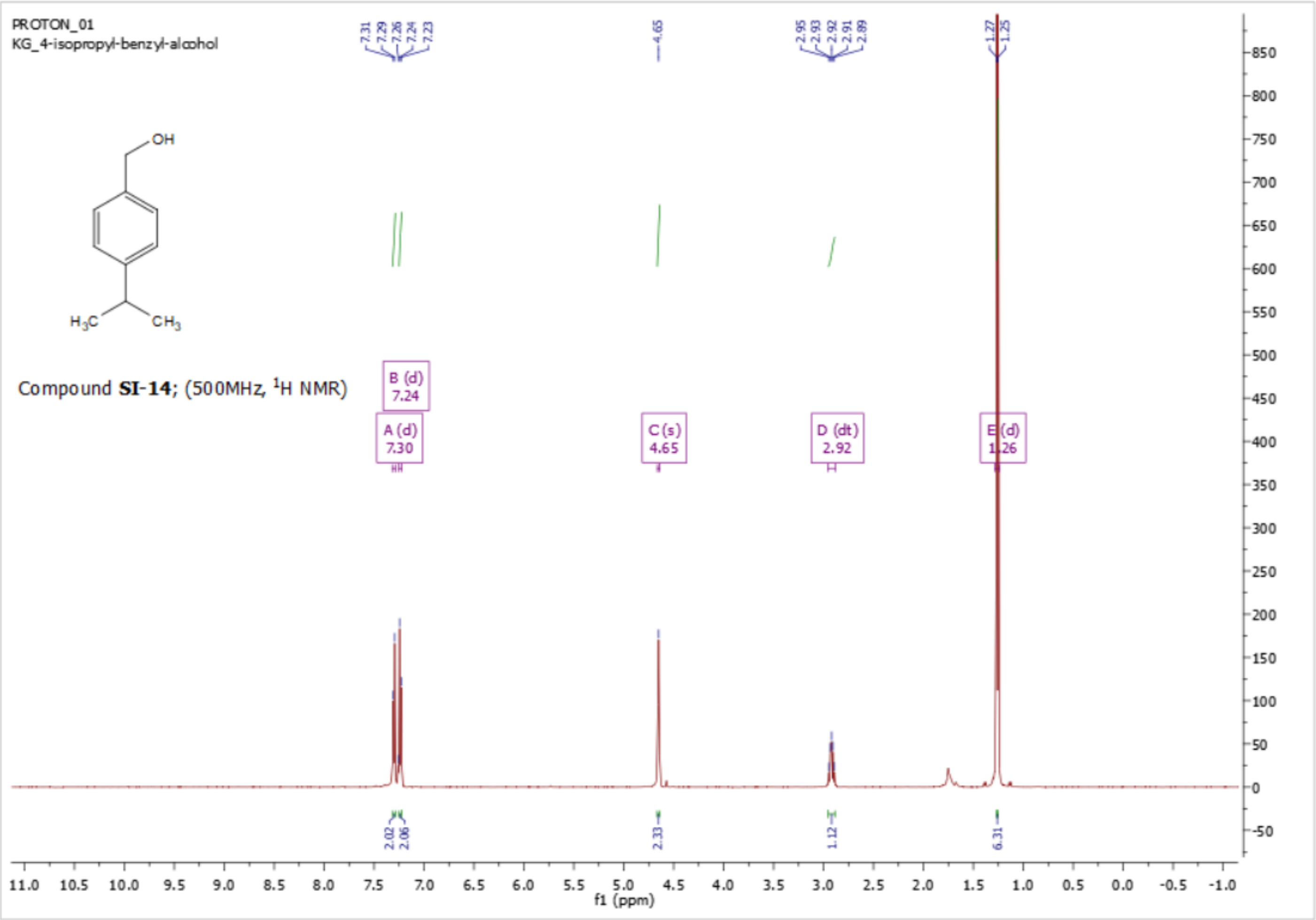
PROTON_01
KG_4-isopropyl-benzyl-alcohol
OH
H3C
CH3
Compound SI-14; (500MHz, 1H NMR)
7.31 7.29 7.26 7.24 7.23
4.65
2.95 2.93 2.92 2.91 2.89
1.27 1.25
A (d) 7.30
B (d) 7.24
C (s) 4.65
D (dt) 2.92
E (d) 1.26
2.02 2.06 2.33 1.12 6.31
f1 (ppm)

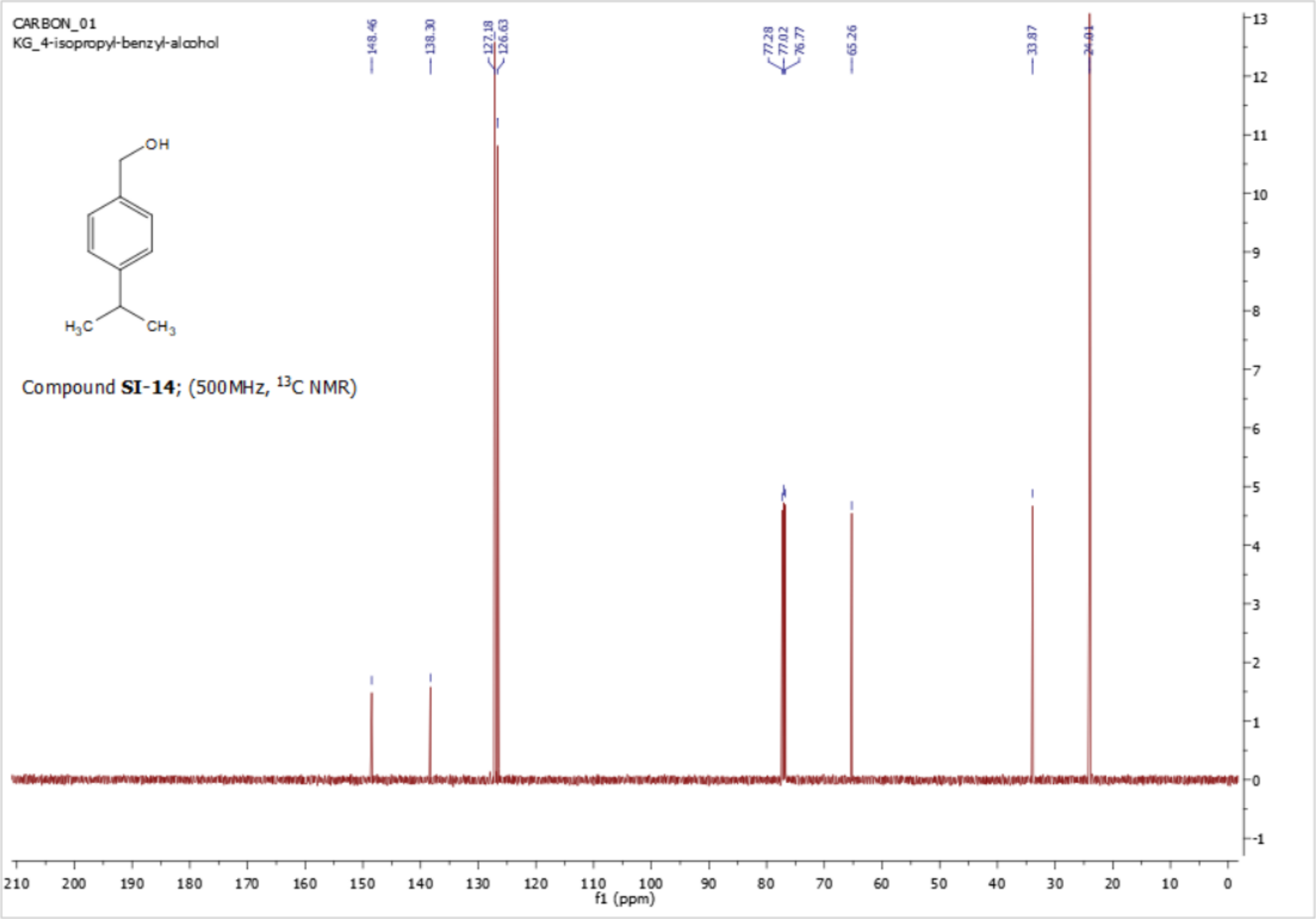
CARBON_01
KG_4-isopropyl-benzyl-alcohol
OH
H3C
CH3
Compound SI-14; (500MHz, 13C NMR)
148.46
138.30
127.18 126.63
77.28 77.02 76.77
65.26
33.87
24.01
f1 (ppm)

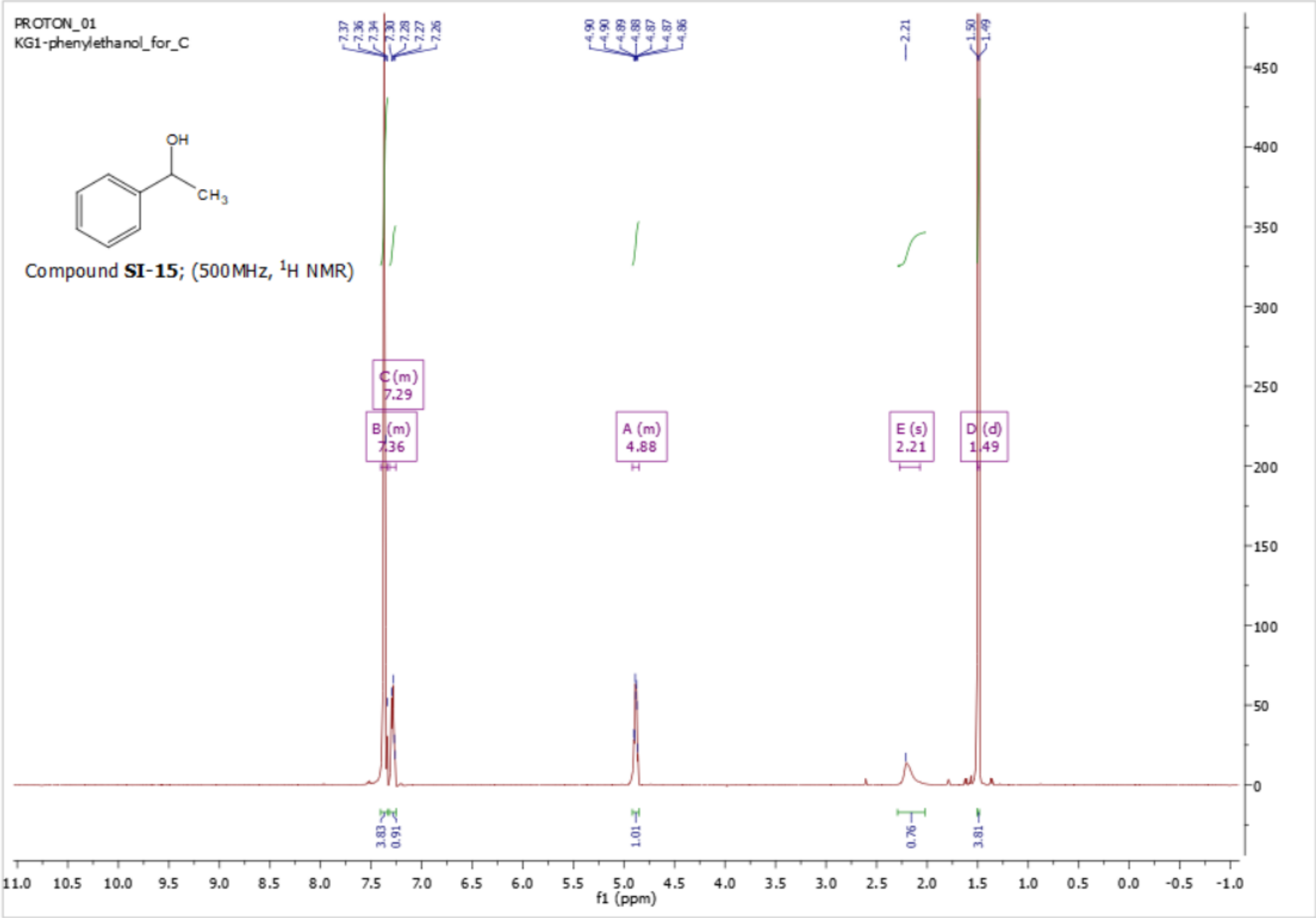
PROTON_01
KG1-phenylethanol_for_C
OH
CH3
Compound SI-15; (500MHz, 1H NMR)
C (m) 7.29
B (m) 7.36
A (m) 4.88
E (s) 2.21
D (d) 1.49
f1 (ppm)

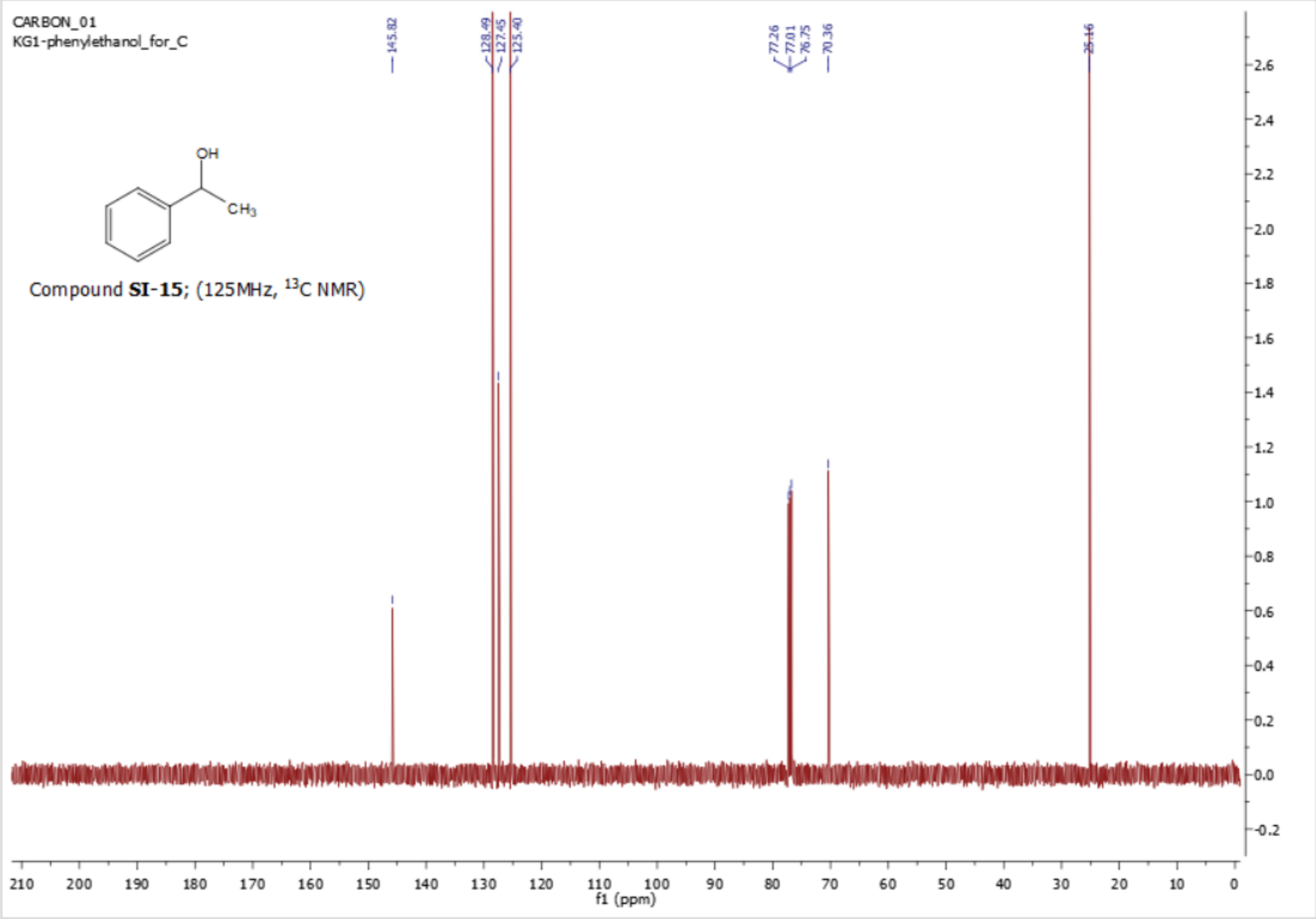
CARBON_01
KG1-phenylethanol_for_C
145.82
128.49
127.45
125.40
77.26
77.01
76.75
70.36
25.16
OH
CH3
Compound SI-15; (125MHz, 13C NMR)
f1 (ppm)

8. References

(1) M. Petsi, A. L. Zografos, *ACS Catal.* **2020**, *10*, 7093–7099.
(2) M. Petsi, M. Orfanidou, A. L. Zografos, *Green Chem.* **2021**, *23*, 9172–9178.
(3) K. Gennaiou, M. Petsi, B. Kakarikas, N. Iordanidis, A. L. Zografos, *Adv. Synth. Catal.* **2022**, *364*, 3059–3065.
(4) K. Gennaiou, A. Kelesidis, A. L. Zografos, *Org. Lett.* **2024**, *26*, 2934–2938.
(5) C. Vergne, J. Appenzeller, C. Ratinaud, M-T. Martin, C. Debitus, A. Zaparucha, A. Al-Mourabit, *Org. Letters* **2008**, *10*, 493-496.
(6) S. Chen, M. S. Hossain, F. Jr W. Foss, *ACS Sustainable Chem. Eng.* **2013**, *1*, 1048-1051.
(7) M. Galeotti, M. Bietti, M. Costas, *J. Am. Chem. Soc.* **2024**, *146*, 8904–8914.
(8) C. Clarasó, L. Vicens, A. Polo, M. Costas, *Org. Lett.* **2019**, *21*, 2430–2435.
(9) A. Call, G. Capocasa, A. Palone, L. Vicens, E. Aparicio, N. Choukairi Afailal, N. Siakavaras, M. E. López Saló, M. Bietti, M. Costas, *J. Am. Chem. Soc.* **2023**, *145*,18094–18103.
(10) M. Milan, M. Bietti, M. Costas, *ACS Cent. Sci.* **2017**, *3*, 196–204.
(11) R. V. Ottenbacher, K. P. Bryliakov, E. P. Talsi, *Adv. Synth. Catal*. **2011**, *353*, 885-889.
(12) O. Cussó, I. Garcia-Bosch, D. Font, X. Ribas, J. Lloret-Fillol, M. Costas, *Org. Lett.* **2013**, *15*, 6158–6161.
(13) L. Gómez, M. Canta, D. Font, I. Prat, X. Ribas, M. Costas, *J. Org. Chem.* **2013**, *78*, 1421–1433.
(14) C. J. Bennett, S. T. Caldwell, D. B. McPhail, P. C. Morrice, G. G. Duthie, R. C. Hartley, *Bioorganic & Medicinal Chemistry* **2004**,*12*, 2079-2098.
(15) D. P. Lubov, M. V. Shashkov, A. A. Nefedov, K. P. Bryliakov, *Org. Lett.* **2023**, *25*,1359–1363.
(16) Yat-Sing Fung, Siu-Cheong Yana, Man-Kin Wong, *Org. Biomol. Chem.*, **2012**,*10*, 3122-3130.
(17) T. V. Le, I. Romero, O. Daugulis, *Chem. Eur. J.* **2023**, *29*, e202301672.
(18) K. Hattori, H. Sajiki, K. Hirota, *Tetrahedron* **2001**, *57*, 2109-2114.
(19) M. M. L. Nieuwenhuizen, T. F. A. de Greef, R. L. J. van der Bruggen, J. M. J. Paulusse, W. P. J. Appel, M. M. J. Smulders, R. P. Sijbesma, E. W. Meijer, *Chem. Eur. J.* **2010**, *16*, 1601 – 1612.
(20) V. P. Demertzidou, M. Kourgiantaki, A. L. Zografos, *Org. Lett.* **2024**, *26*, 4648–4653.
(21) M. Lesieur, C. Battilocchio, R. Labes, J. Jacq, C. Genicot, S. V. Ley, Patrick Pasau, *Chem. Eur. J.* **2019**, *25*, 1203 – 1207.
(22) I. Prat, L. Gómez, M. Canta, X. Ribas, M. Costas, *Chem. Eur. J.* **2013**, *19*, 1908 – 1913.
(23) W. G. Shuler, S. L. Johnson, M. K. Hilinski, *Org. Lett.* **2017**, *19*, 4790–4793.
(24) Z. Zhuang, T. Sheng, J. X. Qiao, K. Yeung, J. Yu, *J. Am. Chem. Soc.* **2024**, *146*, 17311–17317.
(25) M. C. Cabua, V. Velichko, L. Pilia, D. Moi, F. Secci, *Eur. J. Org. Chem*. **2024**, *27*, e202400226.
(26) A. Barbero, D. Blakemore, I. Fleming, R. Wesley, *J. Chem. Soc., Perkin Trans. 1*, **1997**, 1329-1352.
(27) J. Wang, H. Li, M. Wang, J. Wang, Y. Wu, *Tetrahedron Letters* **2018**, *59*, 945–948.
(28) D. K. Ahn, Y. W. Kang, S. K. Woo, *J. Org. Chem.* **2019**, *84*, 3612–3623.
(29) Q. Yu, D. Zhou, Y. Liu, X. Huang, C. Song, J. Ma, J. Li, *Org. Lett.* **2023**, *25*, 47–52.
(30) Z. Tan, T. Chen, J. Zhu, W. Luo, D. Yu, W. Guo, *J. Org. Chem*. **2024**, *89*, 2656−2664.
(31) N. A. Clanton, N. A. Wilson, E. Ortiz, S. T. Blumberg, D. E. Frantz, *Org. Lett.* **2023**, *25*, 277–281.
(32) S. Thiyagarajan, C. Gunanathan, *Org. Lett.* **2019**, *21*, 9774–9778.
